# A Cu^I^_6_L_4_ Cage Dynamically Reconfigures to Form Suit[4]anes and Selectively Bind Fluorinated Steroids

**DOI:** 10.1021/jacs.4c00257

**Published:** 2024-04-05

**Authors:** Natasha M. A. Speakman, Andrew W. Heard, Jonathan R. Nitschke

**Affiliations:** † Yusuf Hamied Department of Chemistry, University of Cambridge, Lensfield Road, Cambridge CB2 1EW, U.K.; ‡ Astex Pharmaceuticals, 436 Cambridge Science Park, Milton Road, Cambridge CB4 0QA, U.K.

## Abstract

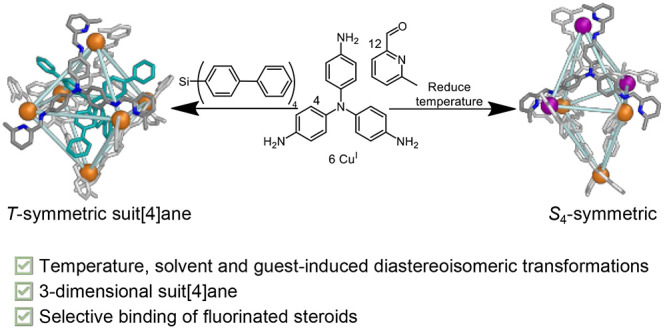

Simple organic ligands can self-assemble with metal ions to generate metal–organic cages, whose cavities bind guests selectively. This binding may enable new methods of chemical separation or sensing, among other useful functions. Here we report the preparation of a Cu^I^_6_L_4_ pseudo-octahedral metal–organic cage, the ligands of which self-assemble from simple organic building blocks. Temperature, solvent, and the presence of different guests governed which structure predominated from a dynamic mixture of cage diastereomers with different arrangements of right- or left-handed metal vertices. Dissolution in dimethyl sulfoxide or the binding of tetrahedral guests led to a chiral tetrahedral *T*-symmetric framework, whereas low temperatures favored the achiral *S*_4_-symmetric diastereomer. Tetrahedral guests with long arms were encapsulated to form mechanically bonded suit[4]anes, with guest arms protruding out through host windows. The cage was also observed to bind fluorinated steroids, an important class of drug molecules, but not non-fluorinated steroids, providing the basis for new separation processes.

The internal cavities of metal–organic cages have found applications in catalysis,^1−3^ drug delivery,^4,5^ sensing,^6^ chemical separations,^7,8^ and the stabilization of reactive species.^9^ A significant number of these metal–organic cages are formed via subcomponent self-assembly.^10^ In this approach, dynamic covalent bonds (often C=N) and coordination bonds (N→metal) allow the formation of the ligand *in situ*.

Whereas metal ions with square-planar^11,12^ and octahedral^13,14^ coordination geometries are widely used in metal–organic cages, tetrahedral copper(I) has seen less use.^15,16^ Although Cu^I^–imine mononuclear complexes^17,18^ and helicates^19,20^ have been reported, fewer examples of Cu^I^–imine coordination cages with internal cavities have been reported.^21^ The dynamic nature of metal–organic coordination cages can allow their transformation in response to stimuli,^22^ including light,^23,24^ concentration,^25,26^ temperature,^27^ guest presence,^28−30^ and solvent.^31,32^ However, fewer cages have been reported to respond to combinations of these stimuli.^33^

The novel class of suit[*n*]anes, introduced by Stoddart’s group,^34^ are a class of interlocked supramolecular assemblies where multiple arms of a guest protrude through the apertures of a polycyclic host, resulting in mechanical bonding^35−37^ between guest and host.^34,38−46^

Here we report the formation of a Cu^I^_6_L_4_ metal–organic cage that transforms between diastereomeric configurations in response to the temperature, solvent, and guest binding. In the presence of tetrahedral guests with elongated arms, a mechanically interlocked suit[4]ane^34^ was formed, with each arm protruding through a pore of the *T*-symmetric cage, representing the first three-dimensional suitane of which we are aware. Our cage also shows selective binding of fluorinated corticosteroids over their non-fluorinated analogues.

The assembly of trianiline (**A**) (2 equiv), 6-methyl-2-formylpyridine (**B**) (6 equiv), and Cu(MeCN)_4_BF_4_ (3 equiv) in acetonitrile produced Cu^I^_6_L_4_ coordination cage **1** as the uniquely observed product (Figure 1a). The Cu^I^_6_L_4_ composition of cage **1** was confirmed by electrospray ionization mass-spectrometry (ESI-MS) (Figures S8 and S9). Its ^1^H NMR spectrum at 298 K was broader and more complex than expected for a single product diastereomer (Figures S1 and S2), with the ^1^H–^13^C HSQC NMR spectrum (Figure S3) showing 11 distinct imine environments, which we infer to correspond to a collection of diastereomers having copper centers with mixed Δ and Λ handedness (Figure S4). Diffusion-ordered ^1^H NMR spectroscopy (DOSY) indicated that all signals had a diffusion coefficient of 5.28 × 10^–10^ m^2^ s^–1^, corresponding to a solvodynamic radius of 10.9 Å (Figure S7).

**Figure 1:**
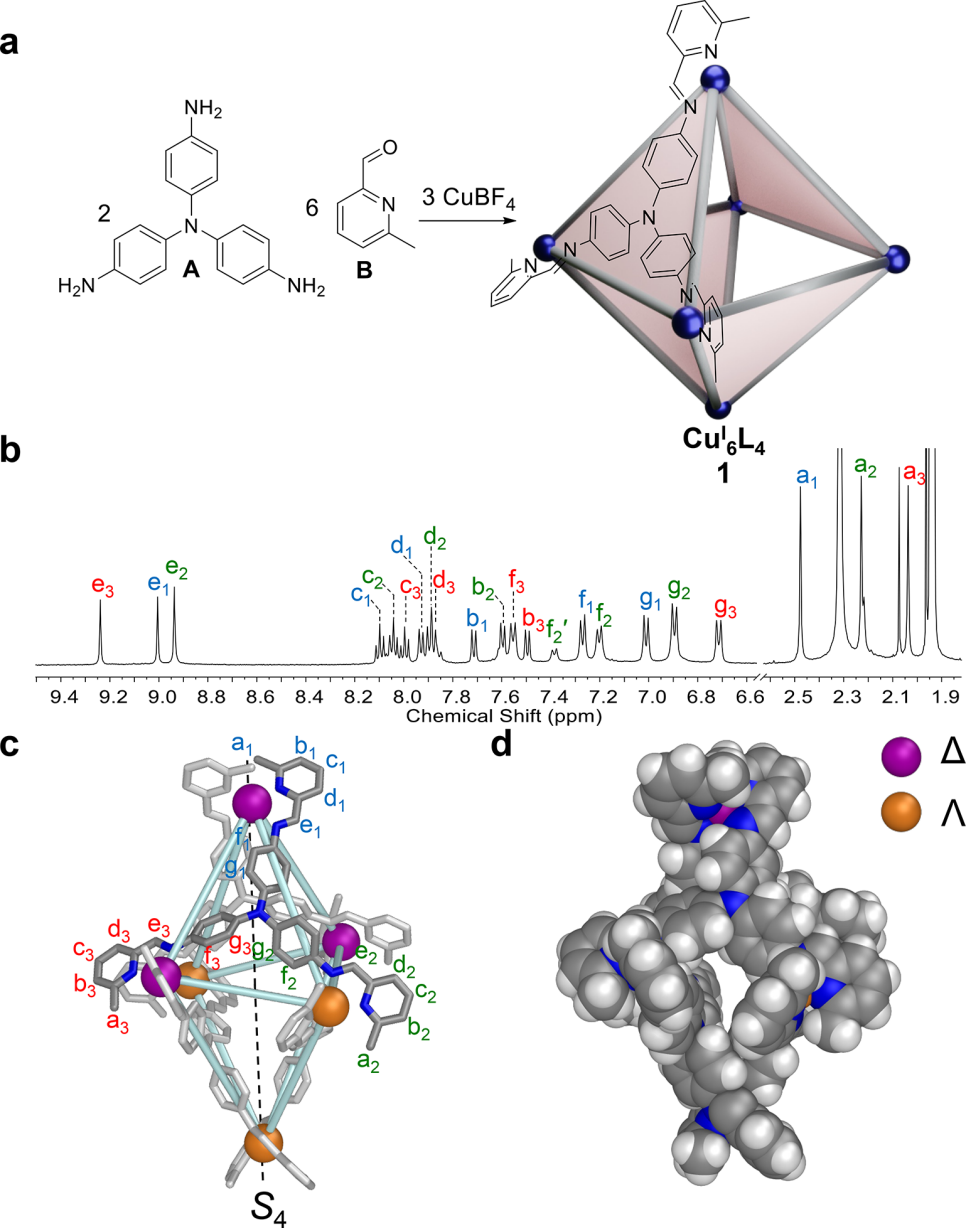
Preparation and characterization of Cu^I^_6_L_4_ cage **1**. (a) The self-assembly of subcomponents **A** and **B** with Cu^I^ in MeCN yielded **1**. The blue spheres in **1** indicate Cu^I^ vertices adopting either Δ or Λ stereochemistry. (b) ^1^H NMR spectrum of *S*_4_-**1** measured at 253 K in CD_3_CN. The signal for each unique ligand arm environment is labeled using a different color; each set could not be conclusively assigned to a specific arm in the structure. (c) Single-crystal X-ray structure of *S*_4_-**1**. (d) Space-filling representation of *S*_4_-**1**.

The solid-state structure of **1** was elucidated via single-crystal X-ray diffraction, as described in Supporting Information (SI) section S5.1. Only the diastereomer of **1** with an approximate *S*_4_ point-group symmetry was observed in the crystal. The *S*_4_ axis passes through antipodal Δ and Λ Cu^I^ vertices (Figure 1c). The cavity of *S*_4_-**1** is elongated, with a Cu^I^(Δ)···Cu^I^(Λ) distance along the *S*_4_ axis of 20.8 Å and Cu^I^(Δ)···Cu^I^(Δ) and Cu^I^(Λ)···Cu^I^(Λ) distances across the cavity of 14.2 and 13.9 Å, respectively (Figure S73).

Upon cooling to 253 K, the ^1^H NMR spectrum of **1** simplified to reflect the presence of only a single species with three distinct ligand environments (Figures 1b, S10, and S12–S20). This signal multiplicity is consistent with **1** adopting either *S*_4_ or *D*_2_ point-group symmetry.

No signal splitting was observed upon addition of the chiral shift reagent Δ-TRISPHAT^47^ (Figure S11), suggesting that the achiral *S*_4_ diastereomer had formed, as opposed to chiral *D*_2_-**1**. This result was consistent with the *S*_4_ conformation adopted by **1** in the crystal (Figures 1c,d and S72–S74).

In room-temperature DMSO-*d*_6_, the ^1^H NMR spectrum of **1** was simpler, showing only two sets of signals in an approximately 4:3 ratio. The major set was assigned to the diastereomer of **1** with *T* point-group symmetry. The minor set showed the same threefold desymmetrization observed for the *S*_4_-**1** isomer observed at 253 K in acetonitrile (Figures S21–S27). All signals shared the same DOSY diffusion coefficient (Figure S28), and ESI-MS (Figures S29 and S30) confirmed a Cu^I^_6_L_4_ composition. We thus infer that **1** exists as an approximately 3:4 mixture of *S*_4_-**1** and *T*-**1** in DMSO, in contrast to the more complex mixture of diastereomers observed in acetonitrile.

Host **1** bound tetrahedral guests **G1**, **G2**, and **G3** (Figure 2b and SI sections S3 and S4). Upon binding one of these guests, the host adopted a *T*-symmetric framework, as evidenced by the presence of only a single set of ligand NMR signals at room temperature (Figures S31, S45, and S58). DOSY NMR studies revealed **G1** to bind in fast exchange (Figure S42), whereas **G2** and **G3** bound in slow exchange (Figures S55 and S69). The ^1^H DOSY NMR spectra of **G2**⊂*T*-**1** and **G3**⊂*T*-**1** (Figures S55 and S69) showed all signals corresponding to host and guest diffusing together, with solvodynamic radii of 13.2 and 12.3 Å, respectively. Correlations observed in ^1^H–^1^H ROESY spectra (Figures S40 and S41, Figures S53 and S54, and Figures S67 and S68) were consistent with internal binding of all three guests.

**Figure 2:**
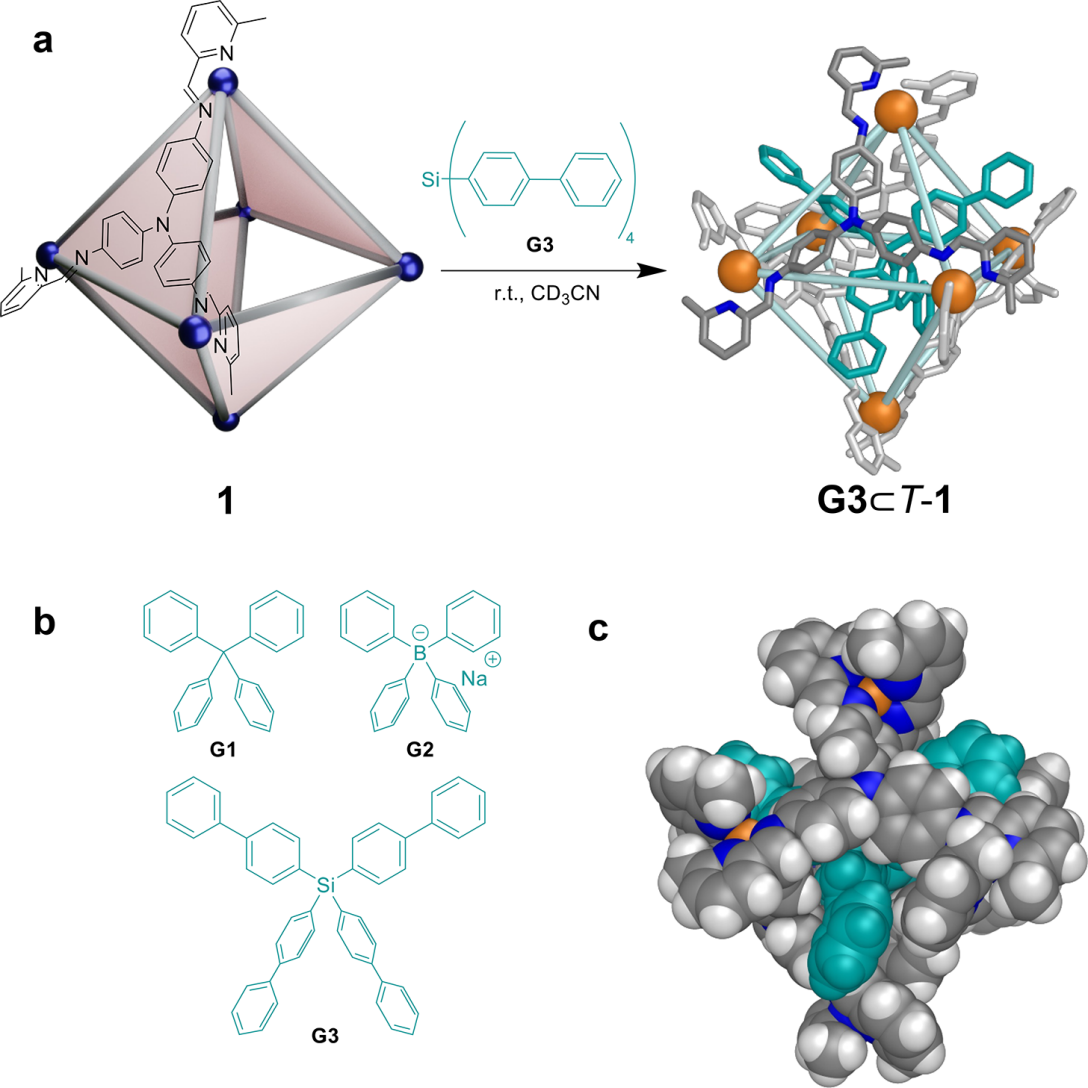
Formation of three-dimensional suit[4]ane **G3**⊂*T*-**1**. (a) Transformation of **1** to mechanically interlocked **G3**⊂*T*-**1** following the addition of **G3**. The blue spheres in **1** indicate Cu^I^ vertices adopting either Δ or Λ stereochemistry. (b) The three tetrahedral guests that induced the *S*_4_-**1** to *T*-**1** transformation upon guest encapsulation. (c) Space-filling representation of the X-ray crystal structure of **G3**⊂*T*-**1**.

The structure of **G3**⊂*T*-**1** was determined via single-crystal X-ray diffraction as described in SI section S5.2. The crystal structure of **G3**⊂*T*-**1** (Figures 2a,c and S75–S77) confirmed the formation of a *T*-symmetric suit[4]ane with an average Cu···Cu separation of 12.1 Å along each edge.

In **G3**⊂*T*-**1**, each of the biphenyl arms of **G3** protrudes through the center of a triangular pore, forming an interlocked suit[4]ane.^38^ Although tetrahedral guest molecules have previously been encapsulated within the cavities of cages,^32^ to the best of our knowledge **G3**⊂*T*-**1** is the first example of a suit[*n*]ane with a three-dimensional central axle component protruding through a nonplanar array of pores in a mechanically bonded configuration.^38^ Its structure thus builds upon those of previously reported suit[*n*]anes, which have rigid planar axles protruding through coplanar macrocyclic pores.^34,38−41^

The change in metal handedness between *S*_4_-**1** (three Δ and three Λ vertices) and *T*-**1** (homochiral vertices) that occurred during the binding of large aromatic tetrahedral guests that cannot fit through the pores of *S*_4_-**1** suggests a mechanism of guest binding that involves disassembly and reassembly of **1**. The likely mechanism involves the disassembly of some vertices of *S*_4_-**1**, the encapsulation of the guest molecule, and the subsequent reassembly of the framework of *T*-**1** around the guest.

Due to their strong binding to **1** and the poor solubilities of **G1** and **G3** in acetonitrile, we were not able to determine association constants for **G1**⊂**1** and **G3**⊂**1**. A binding constant of **G2** for **1** of 5.4(2) × 10^6^ M^–1^ was measured by isothermal titration calorimetry (ITC) (Figure S78).

Anionic **G2** bound in slow exchange on the NMR time scale, likely due to the electrostatic interactions between **G2** and the cationic framework of **1**. **G3** also bound in slow exchange due to its biphenyl arms protruding through the open pores, requiring significant disassembly and reassembly of the cage framework for guest release and re-encapsulation. Conversely, **G1** bound in fast exchange due to a lack of electrostatic interactions and its shorter phenyl arms, allowing for more rapid guest release and encapsulation.

Cage **1** was also observed to bind steroids, a class of molecules with biological and pharmacological relevance.^48^ Eight steroids (**G4**–**G11**; Figures 3b and S79) were screened for interactions with **1** by ^1^H NMR spectroscopy. Changes in the complex ^1^H NMR spectrum of **1** were not apparent upon the addition of any of the steroids shown in Figure 3b at room temperature. However, upon cooling to 253 K, the ^1^H NMR spectrum of a solution of **1** containing excess **G10** or **G11** displayed a set of new cage signals alongside the signals assigned to *S*_4_-**1** (Figures S80 and S81 and Figures S97 and S98). The new signals had multiplicities consistent with the presence of the *T*-**1** diastereomer.

**Figure 3:**
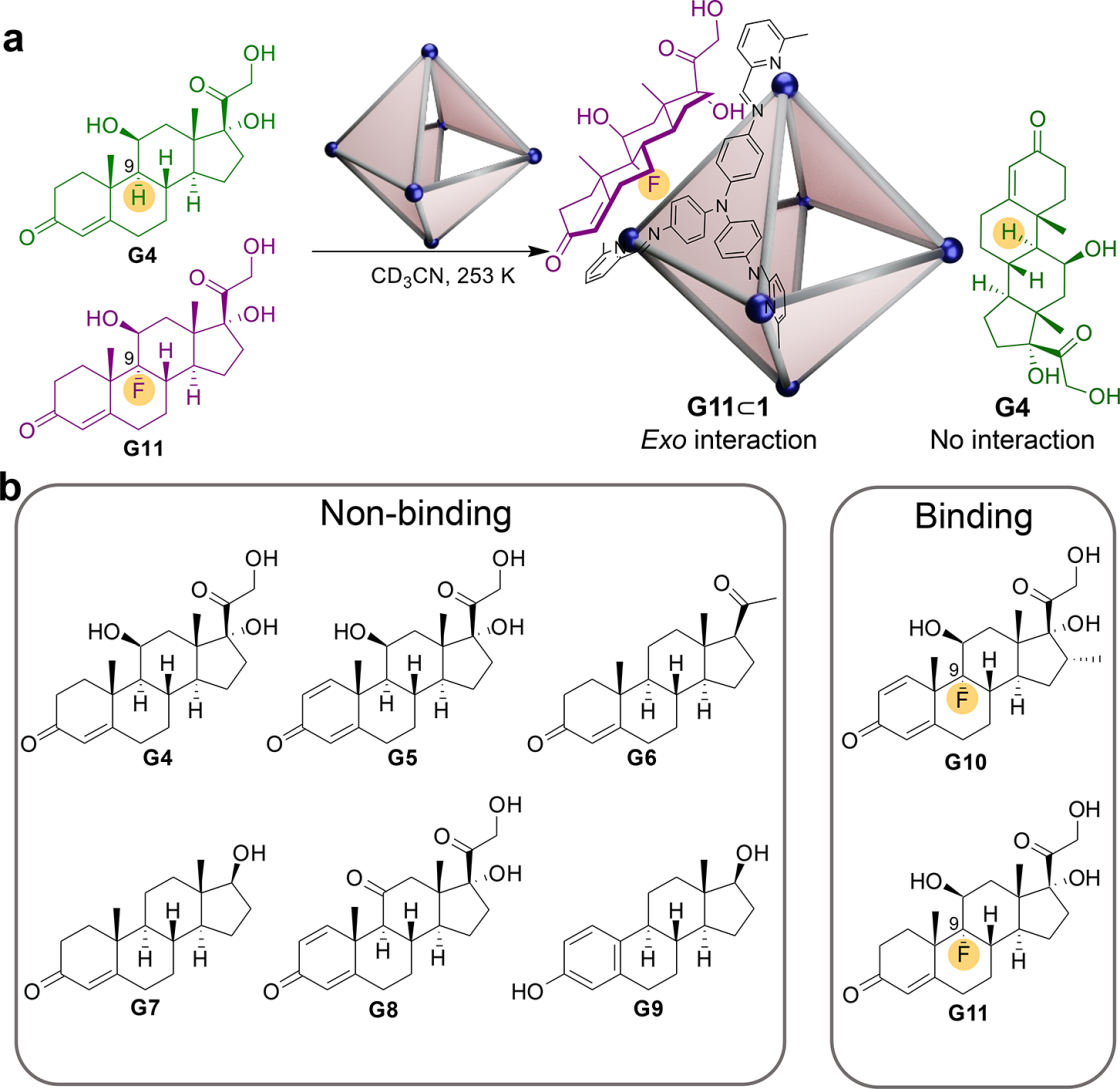
Cage **1** bound fluorinated corticosteroids selectively. (a) Cartoon illustrating the putative *exo* binding mode of fluorinated **G11** by **1** over its non-fluorinated analogue **G4**. (b) The corticosteroids screened, showing those observed to bind and those that did not. The fluorine atoms in **G10** and **G11** are highlighted.

In an ideal system, we would see selective steroid binding closer to room temperature. Work is ongoing to achieve this goal in the future with subsequent iterations of this system. However, the unusual selectivity for the cage toward fluorinated steroids is notable and warranted further investigation of the binding behavior.

^1^H-decoupled ^19^F NMR spectroscopy of **G10**⊂**1**, **G11**⊂**1**, free **G10**, and free **G11** (Figures S83 and S100) showed shifts in the ^19^F signals of **G10** and **G11**, indicating interactions between **1** and **G10** or **G11**. Upon the addition of **1**, shifts in the ^1^H signals of **G10** and **G11** were also observed (Figures S82 and S99), consistent with host–guest interactions in fast exchange on the NMR time scale. In contrast, no changes were observed in the ^1^H NMR spectra for **1** containing **G4**–**G9** (Figures S90–S95).

**G4** and **G11** are homologues, except that **G4** has a hydrogen atom at C9, where **G11** contains fluorine (Figure 3a). To test the selectivity of **1** for binding **G4** over **G11**, **1** was dissolved together with these two steroids in CD_3_CN. The ^1^H and ^19^F NMR spectra (Figures S101 and S102) showed shifts only in those signals assigned to **G11**, consistent with the selective binding of this fluorinated steroid (Figure 3a).

The binding mode of **G11**⊂**1** was investigated using **G3**⊂*T*-**1**, whose cavity is blocked by **G3**, to explore whether the binding of **G11** was *exo* or *endo*. ^1^H and ^1^H-decoupled ^19^F NMR spectroscopy showed very similar shifts in the signals of **G11** for **G11**⊂**1** and **G11**⊂**G3**⊂*T*-**1** (Figures S109 and S110), suggesting an *exo* binding mode.

1D-selective ^1^H–^19^F heteronuclear Overhauser effect spectroscopy (HOESY) showed correlations between the fluorine of **G11** and protons H_f_ and H_g_ of the *T*-**1** diastereomer (Figures S111 and S112), indicating that **G11** bound across the closed faces of **1**.

The stereochemical reconfiguration of Cu^I^_6_L_4_ metal–organic cage **1** thus enables it to bind tetrahedral guests, generating a new class of three-dimensional suit[4]anes. Our work thus paves the way toward the formation of interlocked suit[*n*]anes of higher order, expanding this class of mechanically interlocked materials.^44^ The ability of **1** to selectively bind fluorinated corticosteroids also provides foundations for novel chemical separation processes^7^ that take advantage of the subtle differences in binding between similar molecules. Larger and more complex metal–organic cages that incorporate copper(I) thus represent appealing targets for future studies.

## Supporting Information Available

Synthesis and characterization data for cage **1**, characterization data for the diastereselective transformations of **1**, and characterization data for the host–guest chemistry of **1** (PDF)
