# Supplementary material for: A CuI6L4 Cage Dynamically Reconfigures to Form Suit[4]anes and Selectively Bind Fluorinated Steroids
Source: J Am Chem Soc. 2024 Apr 5;146(15):10234–9. doi: 10.1021/jacs.4c00257 (PMC11027141; doi:10.1021/jacs.4c00257)
Supplement: Supplementary file 1 — ja4c00257_si_001.pdf [file ja4c00257_si_001.pdf]

Supplemental information for

A $Cu^{I}_{6}L_{4}$ cage dynamically reconfigures to form suit[4]anes and selectively bind fluorinated steroids

Natasha M. A. Speakman,[1] Andrew W. Heard,[1,2] and Jonathan R. Nitschke*[1]

[1]Yusuf Hamied Department of Chemistry, University of Cambridge, CB2 1EW, U.K.

[2]Astex Pharmaceuticals, 436 Cambridge Science Park, Milton Road, Cambridge, CB4 0QA, U.K.

*email: jrn34@cam.ac.uk

Table of Contents

S1. General Experimental S3

S2. Synthesis and characterization S4

S2.1 Synthesis of Cage **1** S4

S2.2 Temperature-dependent diastereomeric conversion of **1** S10

S2.3 Solvent-dependent diastereomeric conversion of **1** S17

S3. Diastereoinduction with tetrahedral guests S22

S3.1 Characterization of **G1**⊂*T*-**1** S23

S3.2 Characterization of **G2**⊂*T*-**1** S32

S4. Characterization of Suit[4]ane G3⊂*T*-1 .... S40

S5 X-ray crystallography S50

S5.1 X-ray crystallography of S_4-**1** S50

S5.2 X-ray crystallography of **G3**⊂*T*-**1** S53

S6. Binding constants of tetrahedral guests S57

6.1 Procedure S57

S7. Steroid binding in 1 .... S58

S7.1 Characterization of **G10**⊂**1** S58

S7.2 Characterization of **G11**⊂**1** S67

S7.3 Binding mode of **G11**⊂**1** S75

S7.4 ^{1}H NMR assignments of fluorinated steroids in CD_3CN S79

S8. References S87

S1. General Experimental

Unless otherwise stated, all reagents were purchased from commercial sources (Alfa Aesar, Fisher Scientific, Sigma Aldrich, TCI, FluoroChem) and were used without further purification. All reactions were stirred using PTFE-coated magnetic stir bars. Centrifugation of samples was carried out with a 5804/5804R Benchtop Centrifuge.

^{1}H NMR spectra were recorded using a 500 MHz DCH Cryoprobe spectrometer, a 500 MHz AVIII HD Smart Probe spectrometer or a 700 MHz TXO Cryoprobe spectrometer. ^{19}F NMR spectra were recorded using a 400 MHz Avance III HD Smart Probe Spectrometer or a 500 MHz AVIII HD Smart Probe spectrometer. ^{13}C NMR spectra were recorded on a 500 MHz DCH Cryoprobe Spectrometer or a 700 MHz TXO Cryoprobe spectrometer. Chemical shifts (δ) are reported in parts per million (ppm) from low to high field and are referenced to residual solvent. ^{19}F NMR spectra were referenced to an internal standard of hexafluorobenzene (-164.9 ppm, CD_3CN). Coupling constants (*J*) are reported in Hertz (Hz). The following abbreviations are used to describe signal multiplicity in ^{1}H, ^{13}C and ^{19}F NMR spectra: s, singlet; d, doublet; t, triplet; q, quartet; m, multiplet; br, broad. Signal assignment was carried out using 2D NMR methods (DQF-COSY, HSQC, HMBC, ROESY) where necessary. ^{1}H DOSY NMR spectra were recorded using a 400 MHz Avance III HD Smart Probe Spectrometer. Maximum gradient strength was 6.57 G/cm A. The standard Bruker pulse program, ledbpgp2s, employing a stimulated echo and longitudinal eddy-current delay (LED) using bipolar gradient pulses for diffusion using 2 spoil gradients was utilized. Rectangular gradients were used with a total duration of 1.5 ms. Gradient recovery delays were 1250-1500 µs. DOSY NMR spectra were processed in the *Dynamics Center* software. Signal deconvolution was carried out using the 'DCON' workflow package in Topspin. 1D selective ^{1}H-^{19}F HOESY NMR spectra were recorded using a 500 MHz AVIII HD Smart Probe spectrometer. The pulse programme, selhoesyhfgpqn1d, was employed and the fluorine signal of interest was selectively excited with a pulse of bandwidth 5 ppm.

High-Resolution Electrospray Ionization Mass Spectrometry (HR-ESI-MS) was performed on a Waters Synapt G2-Si spectrometer (cone voltage 20 eV, capillary voltage 2.50 kV, desolvation temperature 100 ºC, ionization temperature 60 ºC) infused from a Harvard syringe pump at a rate of 4-10 µL min^{-1} or a Waters Xevo G2-S QTOF spectrometer direct injection bypassing UPLC (cone voltage 20 eV, capillary voltage 2.50 kV, desolvation temperature 100 ºC, ionization temperature 60 ºC).

Low-Resolution Electrospray Ionization Mass Spectrometry (LR-ESI-MS) was performed on a Waters Xevo TQD mass spectrometer (cone voltage 20 eV, capillary voltage 3.00 kV,

desolvation temperature 100 °C) infused from a Harvard syringe pump at a rate of 17 µL min^{-1}.

Isothermal titration calorimetry (ITC) was carried out on an ITC 200 manufactured by Malvern.

S2. Synthesis and characterization

S2.1 Synthesis of Cage **1**

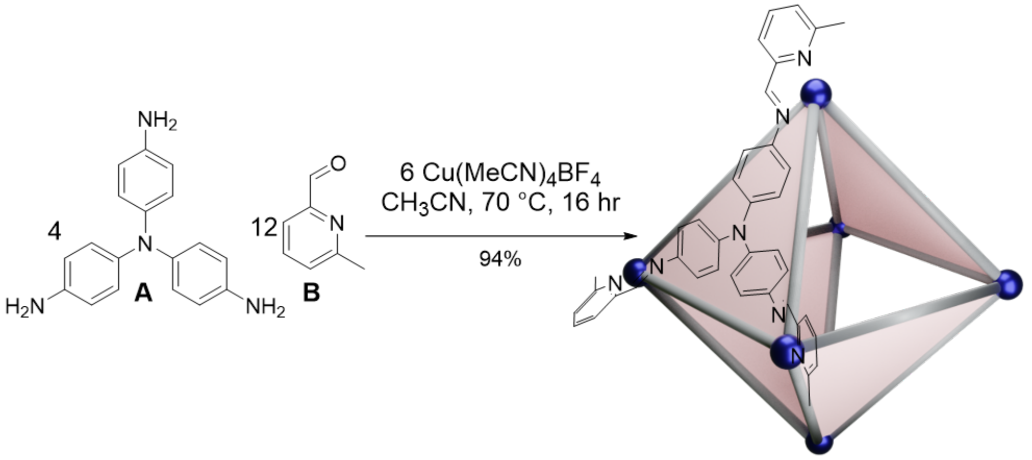

1

Tris(4-aminophenyl)amine **A** (57 mg, 0.20 mmol, 1.0 equiv.), 6-methyl-2-pyridinecarboxaldehyde **B** (71 mg, 0.59 mmol, 3.0 equiv.) and $Cu(MeCN)_4BF_4$ (92.0 mg, 0.292 mmol, 1.5 equiv.) were dissolved in CH_3CN (2.5 mL). The mixture was degassed *via* three cycles of freeze-pump-thaw, then heated at 70 °C for 16 hours. The reaction was cooled to room temperature and precipitated with Et_2O (10 mL), the resulting solid was washed with Et_2O (3 × 10 mL) to yield cage **1** as a brown solid (153 mg, 46.3 µmol, 94%). Cage **1** was synthesized as a mixture of diastereoisomers.

After washing with Et_2O, no signal corresponding to free aldehyde **B** was observed in the ^1H NMR spectrum of cage **1** (Figs. S1 and S2), which suggests partial formation of cage **1** is not occurring. We did not observe any incomplete assemblies of alternative composition in the LR mass spectrum (Fig. S8), further confirming that the sole products correspond to $Cu^I_6L_4$ metal-organic cage diastereomers. Additionally, we did not observe smaller species on the DOSY NMR spectrum (Fig. S7), supporting the formation of $Cu^I_6L_4$ architectures only.

Full characterization of S_4-**1** (Figure S12-Figure S20) and *T*-**1** (Section 3) are below.

LR-ESI-MS [POS] ([**1**] = $C_{156}H_{132}N_{28}Cu_6(BF_4)_6$) *m/z* = 463.4 $[M-6BF_4]^{6+}$ (calc. 463.4), 573.4 $[M-5BF_4]^{5+}$ (calc. 573.4), 738.5 $[M-4BF_4]^{4+}$ (calc. 738.4), 1013.5 $[M-3BF_4]^{3+}$ (calc. 1013.5).

HR-ESI-MS [POS] ([**1**] = $C_{156}H_{132}N_{28}Cu_6(BF_4)_6$) *m/z* = 573.3396 $[M-5BF_4]^{5+}$ (calc. 573.3400), 738.1749 $[M-4BF_4]^{4+}$ (calc. 738.1759), 1013.2335 $[M-3BF_4]^{3+}$ (calc. 1013.3259), 1563.3484 $[M-2BF_4]^{2+}$ (calc. 1563.3563).

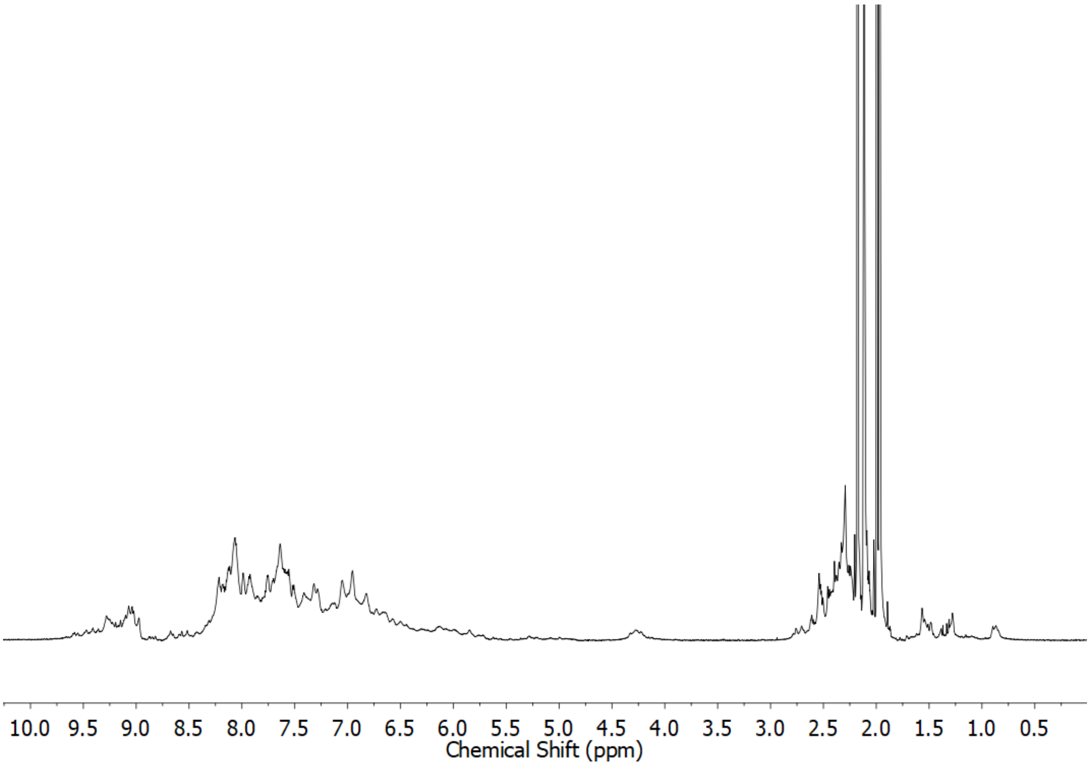

Figure S1: ^{1}H NMR spectrum of **1** (500 MHz, CD_3CN, 298 K). The complexity and broadness of the spectrum is associated with a range of diastereomers being present at 298 K.

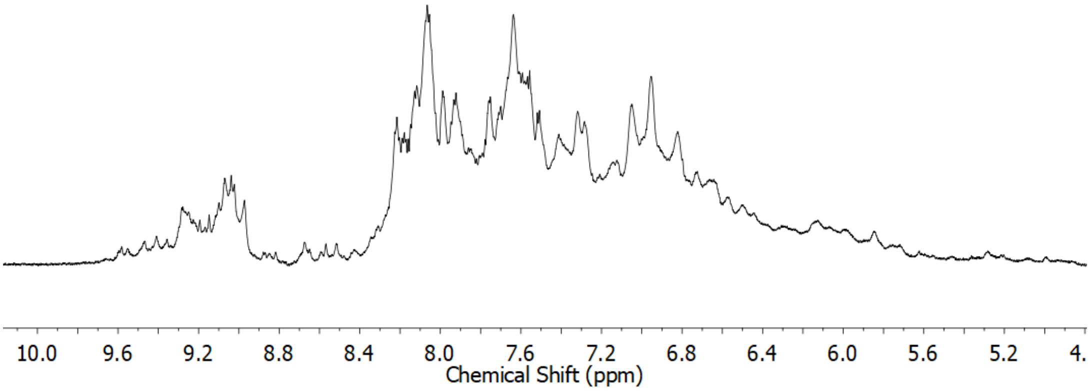

Figure S2: ^{1}H NMR spectrum of the aromatic region of **1** (500 MHz, CD_3CN, 298 K). The range of signals in the region 9.55–8.78 ppm, attributed to imine protons, suggest multiple diastereomers are present.

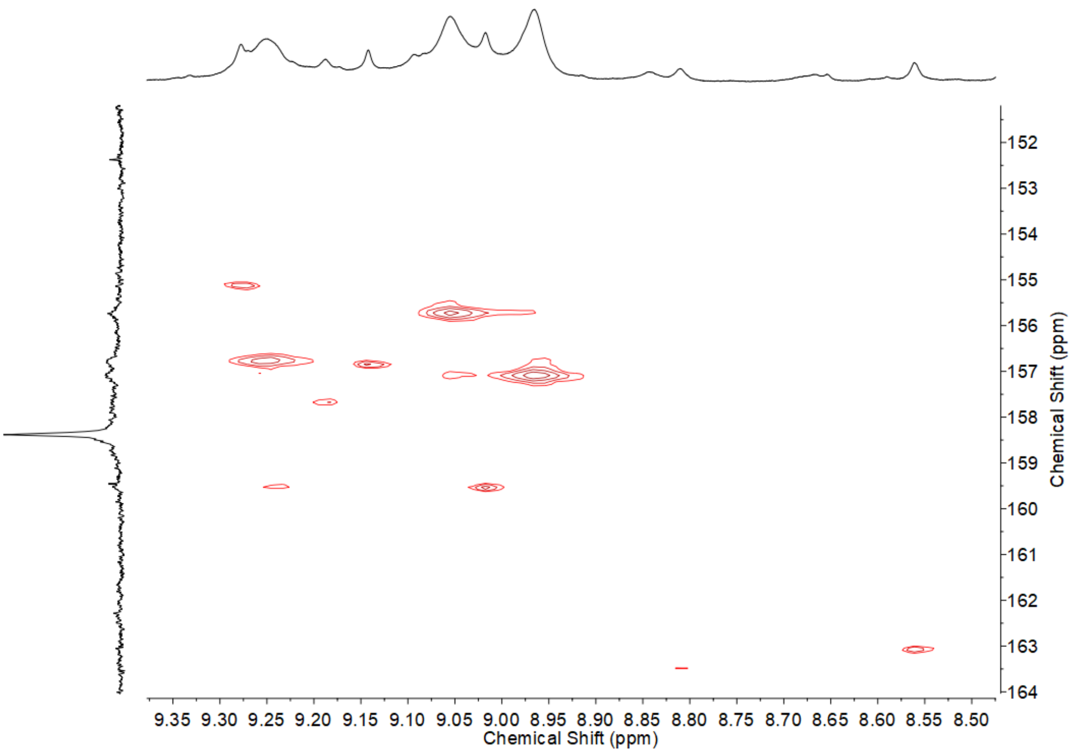

Figure S3: ^{1}H-^{13}C HSQC NMR spectrum of the imine region of **1** (700 MHz, CD_3CN, 298 K). The spectrum shows 11 imine environments, but the actual number may be higher due to the low concentration of some of the diastereomers present.

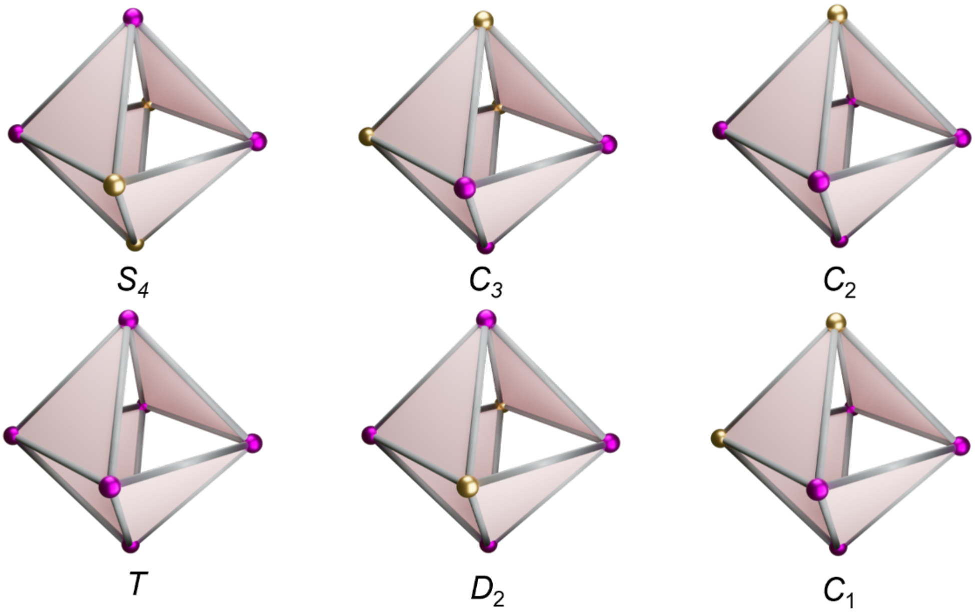

Figure S4: Cartoon representation of the six possible diastereomers of **1**. Purple and orange spheres correspond to different metal handednesses (Δ *vs.* Λ).

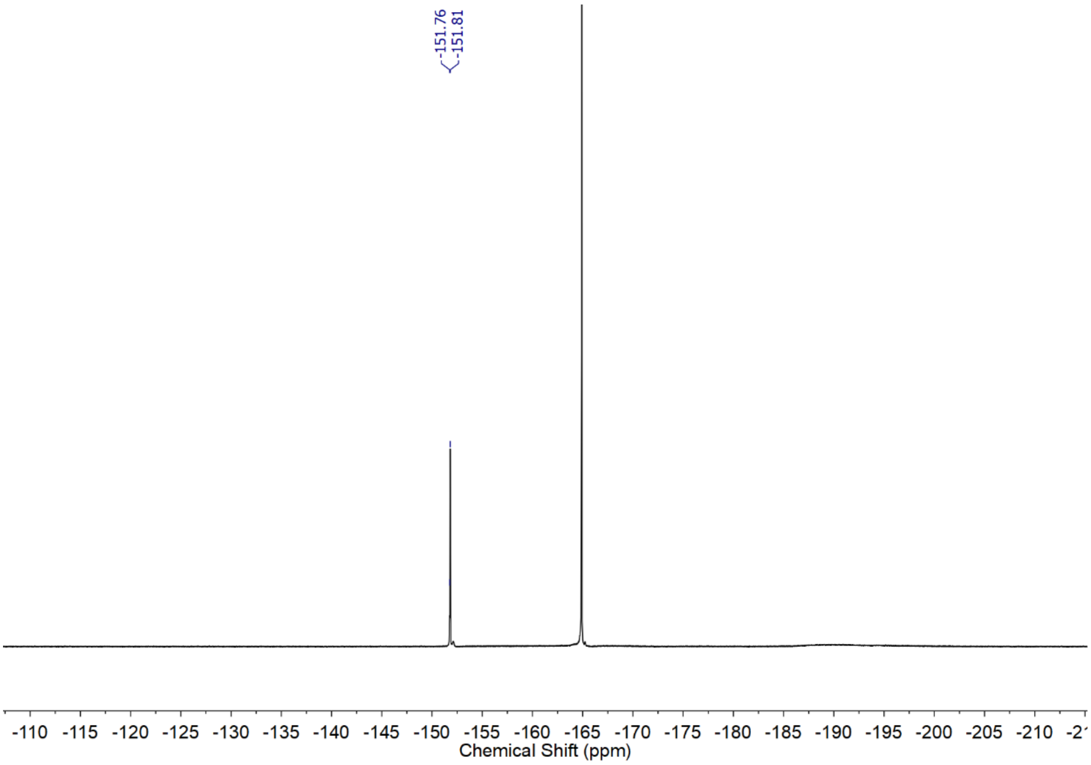

Figure S5: ^{19}F NMR spectrum of **1** (376 MHz, CD_3CN, 298 K, referenced to hexafluorobenzene).

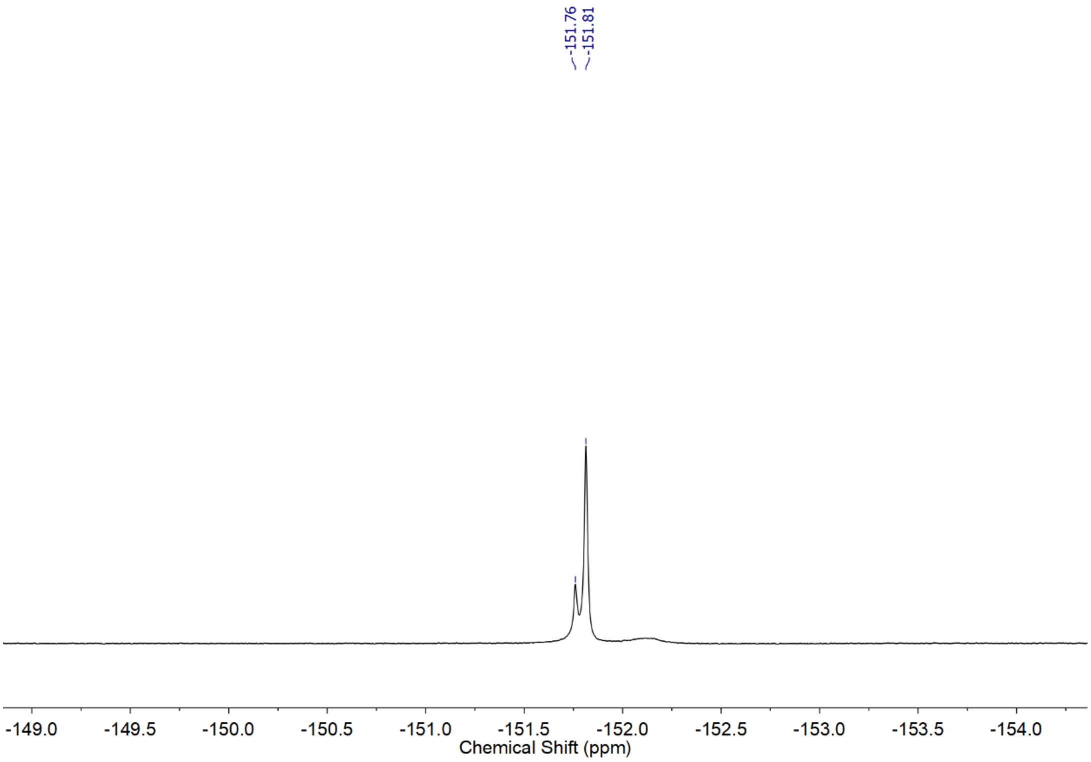

Figure S6: Partial ^{19}F NMR spectrum of **1** showing two peaks due to ^{10}B and ^{11}B isotopomers (376 MHz, CD_3CN, 298 K, referenced to hexafluorobenzene).

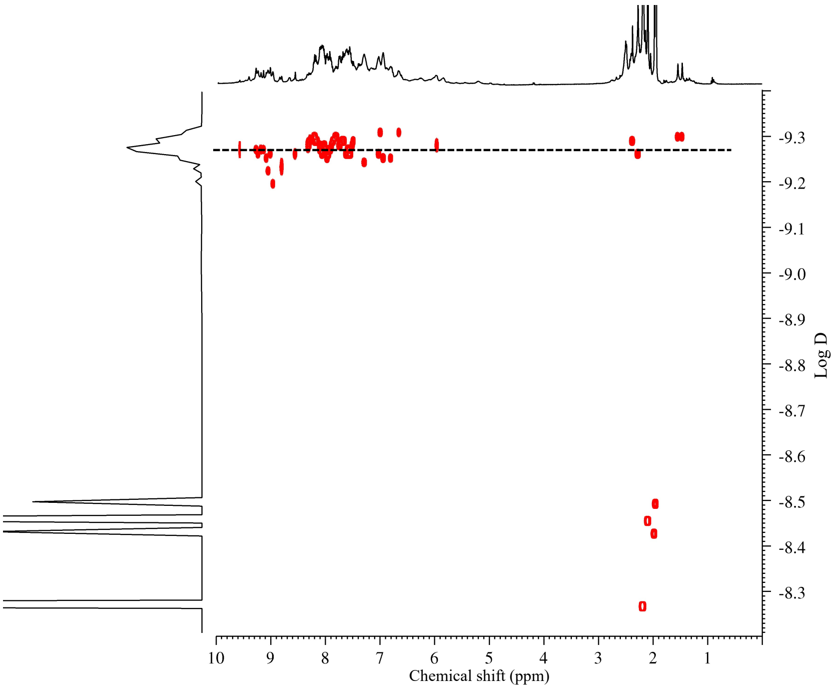

Figure S7: ^{1}H DOSY NMR spectrum of **1** (400 MHz, CD_3CN, 298 K). The diffusion coefficient of **1** was measured to be 5.28×10^{-10} m^2s^{-1}, corresponding to a solvodynamic radius of 10.9 Å. The peaks around 2 ppm are assigned to residual MeCN, H_2O and acetone.

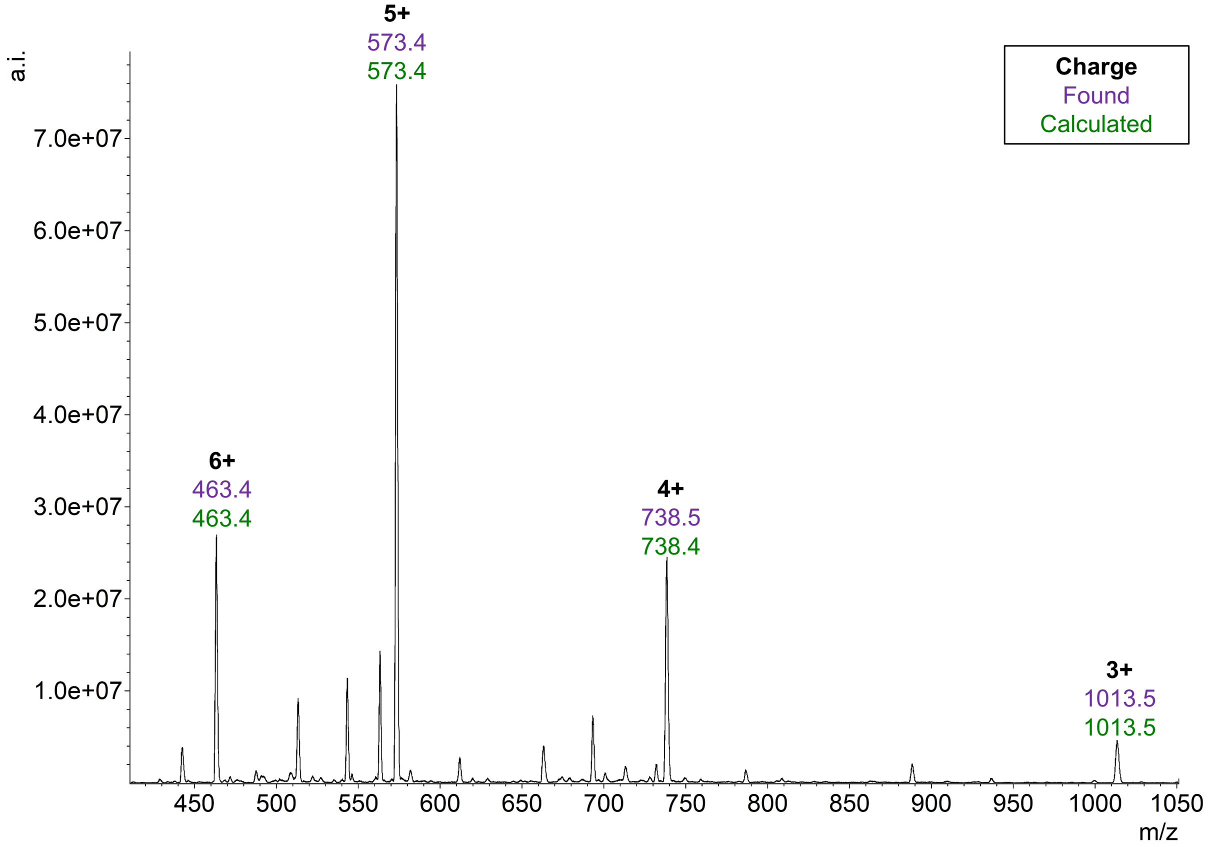

Figure S8: Low-resolution ESI-mass spectrum (CH_3CN) of **1** $[Cu_6L_4](BF_4)_6$ $[M\text{-}nBF_4]^{n+}$.

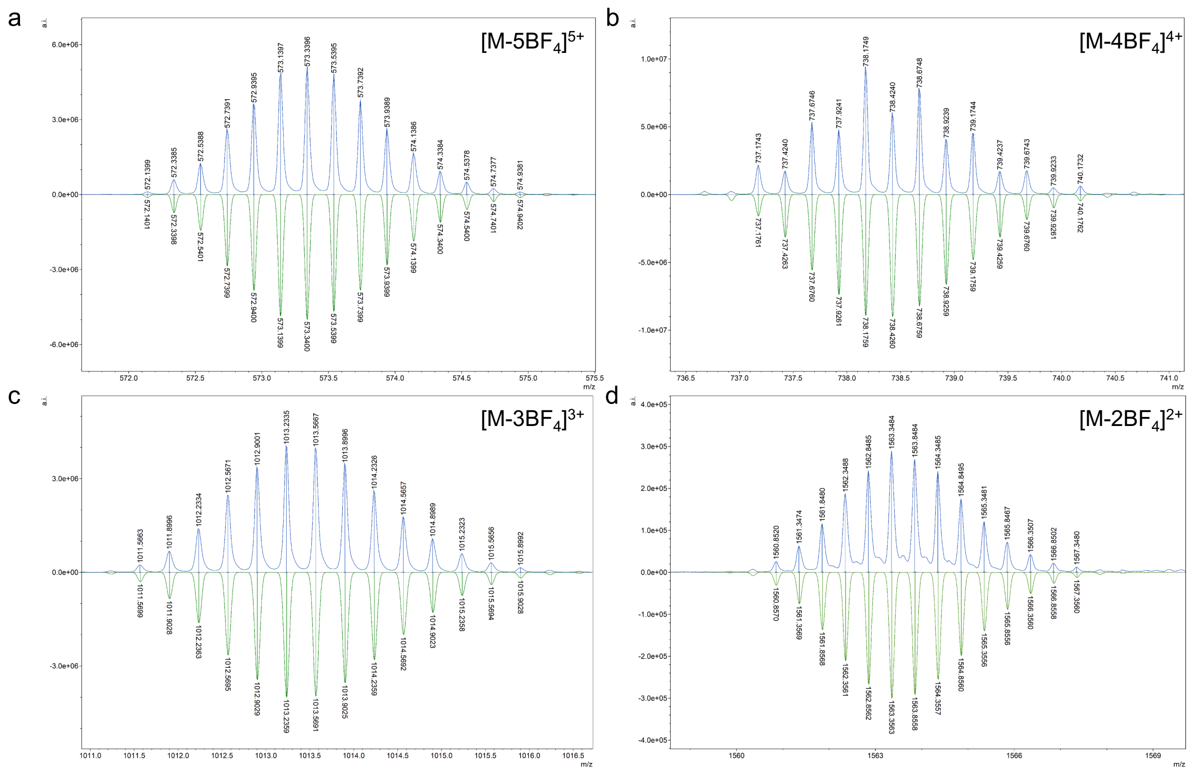

Figure S9: High resolution ESI-mass spectrum peaks of **1**. Experimental (blue) and theoretical (green) peaks are shown for (a) $[M\text{-}5BF_4]^{5+}$; (b) $[M\text{-}4BF_4]^{4+}$; (c) $[M\text{-}3BF_4]^{3+}$; (d) $[M\text{-}2BF_4]^{2+}$.

S2.2 Temperature-dependent diastereomeric conversion of **1**

The ^{1}H NMR spectra of **1** at different temperatures are shown in Fig. S10. As the temperature is decreased the formation of one diastereomer, S_4-**1**, is favored exclusively.

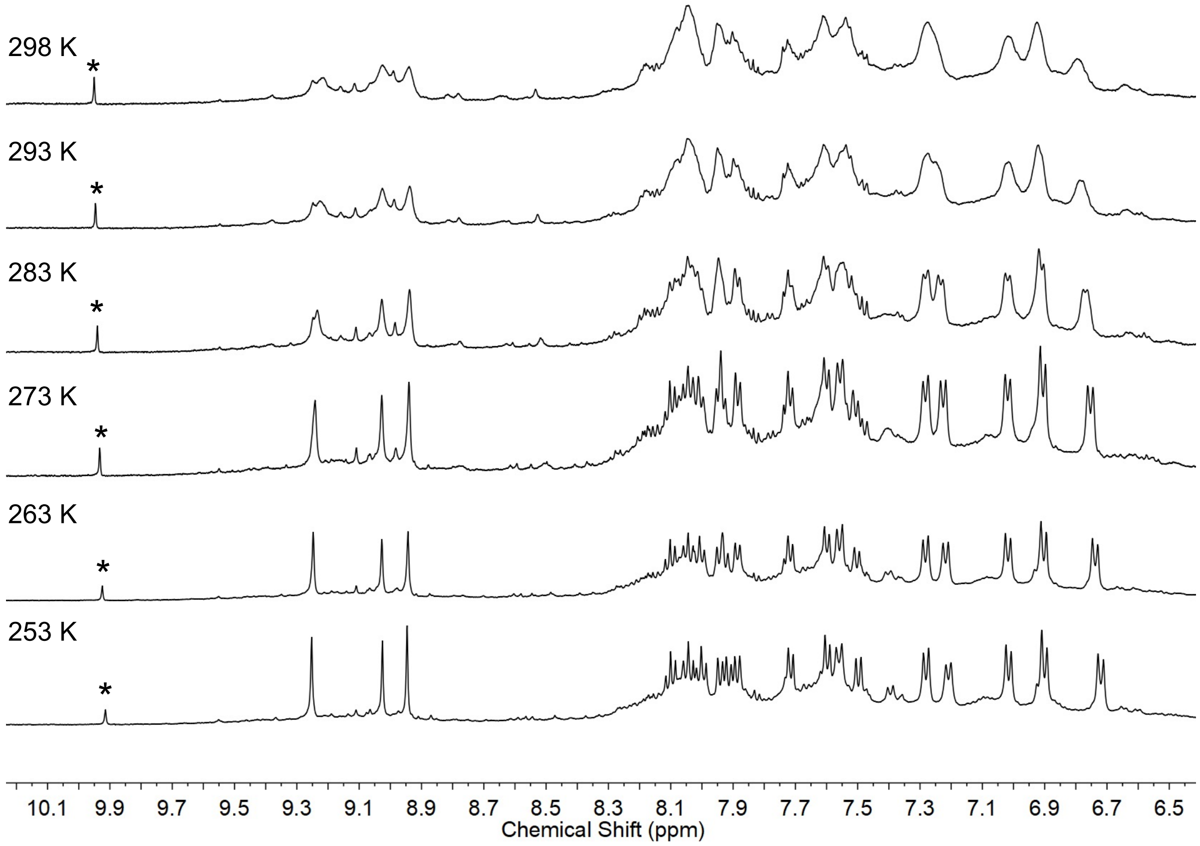

Figure S10: Stacked ^{1}H NMR spectra of the aromatic region of **1** recorded at different temperatures (500 MHz, CD_3CN). The conversion from a range of diastereomers to S_4-**1** diastereomer at 253 K is shown. The aldehyde signals corresponding to excess **B** are marked by asterisks.

S2.2.1 NMR Characterization of S_4-**1**

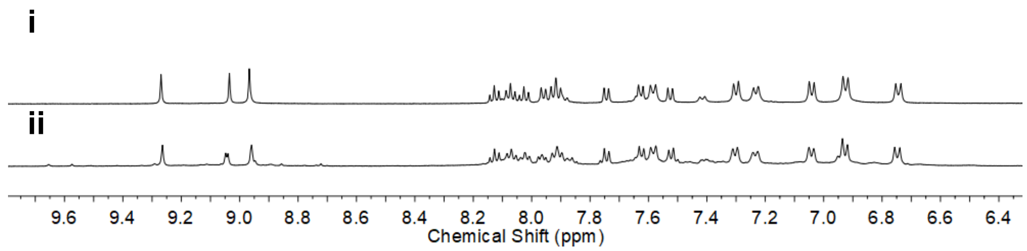

Figure S11: Stacked partial ^{1}H NMR spectra of **i** Cu_6L_4 and **ii** Cu_6L_4 + 8.0 equiv. tetra-*n*-butylammonium Δ-TRISPHAT (500 MHz, CD_3CN, 253 K).

At 253 K, the mixture of diastereroisomers was converted to a diastereoisomerically pure sample of S_4-**1**. The subscripts 1, 2, and 3 refer to the distinct NMR signals corresponding to each distinct ligand environment.

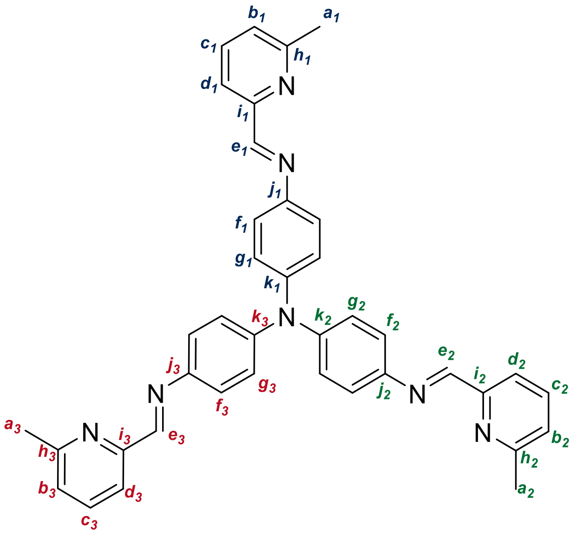

δ_H (CD_3CN, 500 MHz, 253 K) 9.24 (4H, s, H_{e3}), 9.01 (4H, s, H_{e1}), 8.94 (4H, s, H_{e2}), 8.11-7.98 (12H, m, H_{c1}, H_{c2}, H_{c3}), 7.94-7.85 (12H, m, H_{d1}, H_{d2}, H_{d3}), 7.72 (4H, d, J = 7.8 Hz, H_{b1}), 7.60 (4H, d, J = 7.7 Hz, H_{b2}), 7.56 (8H, d, J = 8.9 Hz, H_{f3}), 7.50 (4H, d, J = 7.9 Hz, H_{b3}), 7.27 (8H, d, J = 8.4 Hz, H_{f1}), 7.20 (8H, d, J = 8.3 Hz, H_{f2}), 7.01 (8H, d, J = 8.5 Hz, H_{g1}), 6.90 (8H, d, J = 8.6 Hz, H_{g2}), 6.72 (8H, d, J = 8.9 Hz, H_{g3}), 2.48 (12H, s, H_{a1}), 2.23 (12H, s, H_{a2}), 2.04 (12H, s, H_{a3}).

δ_C (CD_3CN, 126 MHz, 253 K) 159.1 (C_{h1}, C_{h2}, C_{h3}), 157.8 (C_{e2}), 157.3 (C_{e3}), 156.4 (C_{e1}), 151.6 (C_{i1}), 151.3 (C_{i2}), 150.6 (C_{i3}),148.2 (C_{k3}), 147.7 (C_{k1}), 147.5 (C_{k2}), 143.2 (C_{j1}), 142.9 (C_{j2}), 142.3 (C_{j3}), 139.5 (C_{c1}, C_{c2}, C_{c3}), 129.0 (C_{b1}, C_{b2}, C_{b3}), 126.8 (C_{g1}), 126.4 (C_{d1}, C_{d2}, C_{d3}), 125.5 (C_{g3}), 125.3 (C_{f3}), 125.1 (C_{f1}), 124.3 (C_{f2}), 123.3 (C_{g2}), 25.9 (C_{a1}), 25.1 (C_{a2}), 24.4 (C_{a3}).

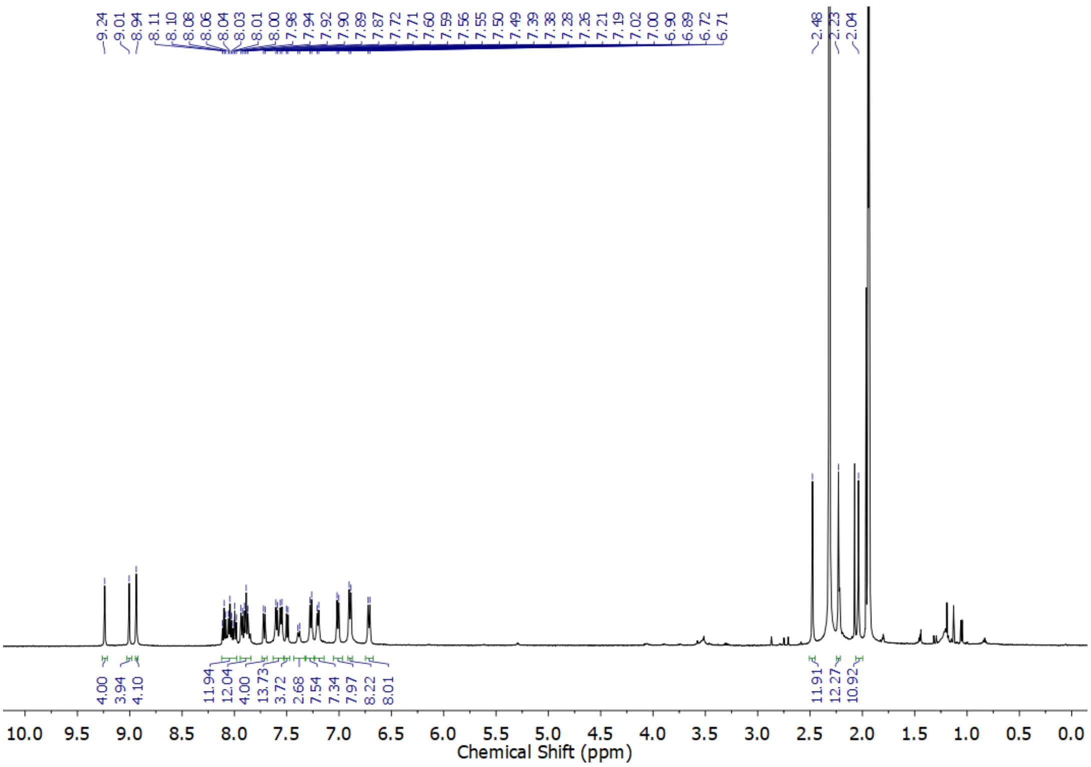

Figure S12: ^{1}H NMR spectrum of S_4-**1** (500 MHz, CD_3CN, 253 K).

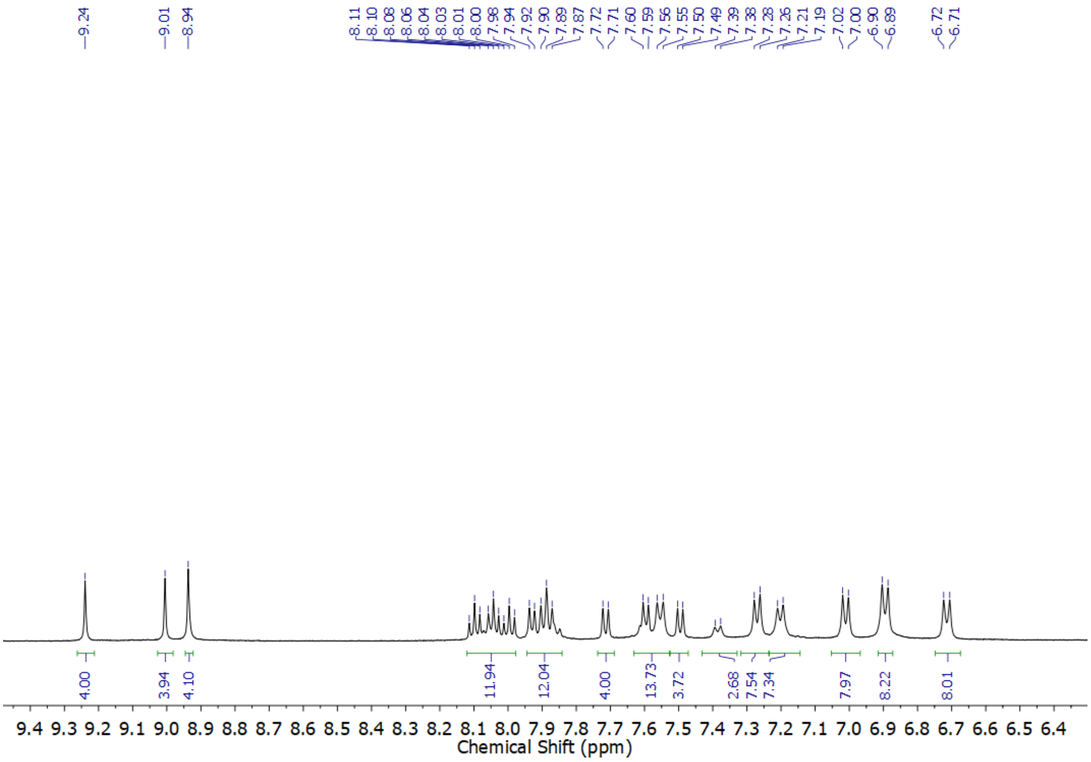

Figure S13: Aromatic region of the ^{1}H NMR spectrum of S_4-**1** (500 MHz, CD_3CN, 253 K).

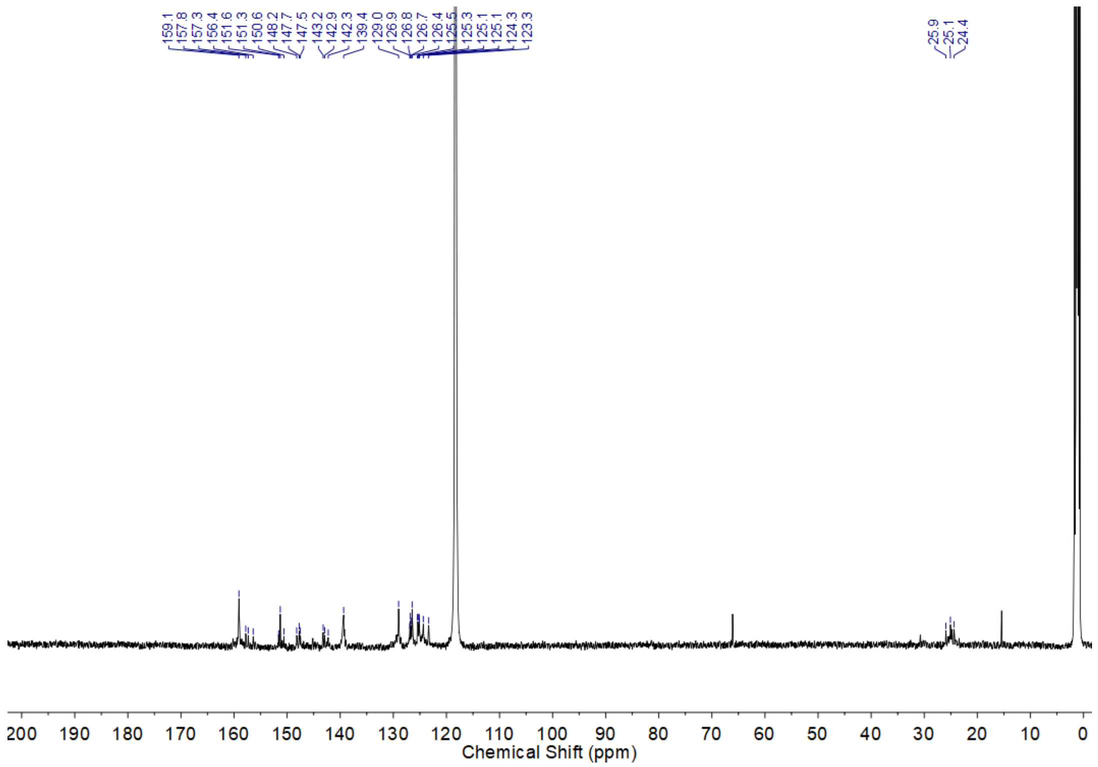

Figure S14: ^{13}C NMR spectrum of S_4-**1** (126 MHz, CD_3CN, 278 K). The peaks at 66.3 and 15.6 ppm are assigned to residual Et_2O.

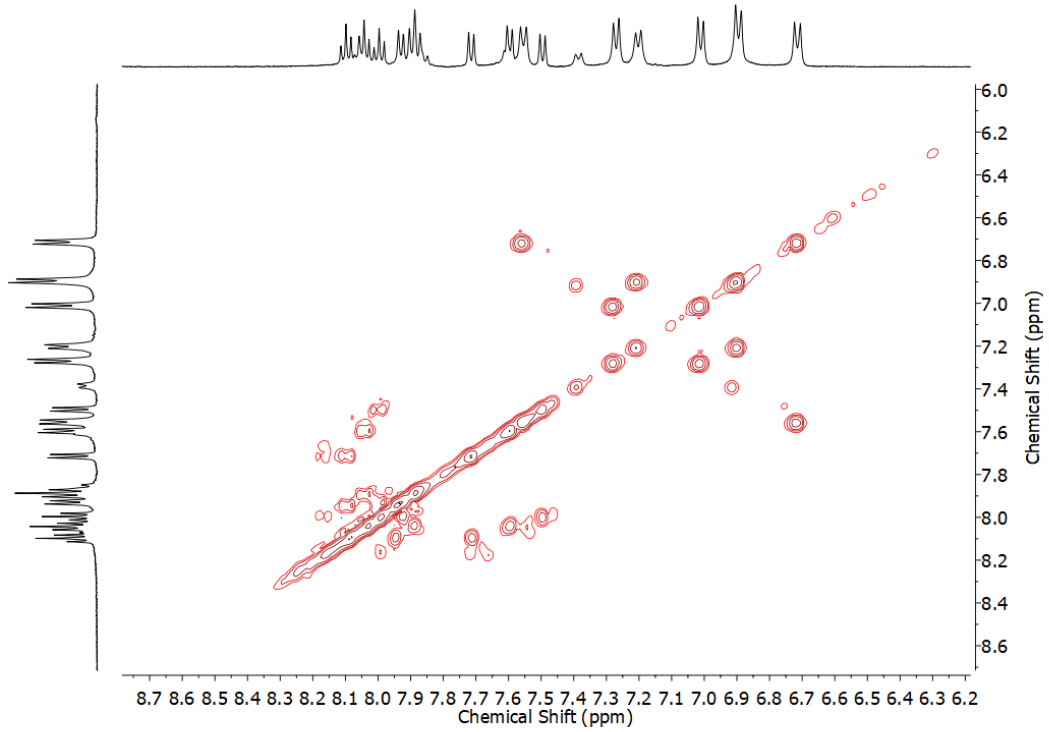

Figure S15: Aromatic region of ^{1}H-^{1}H DQF-COSY NMR spectrum of S_4-**1** (500 MHz, CD_3CN, 253 K).

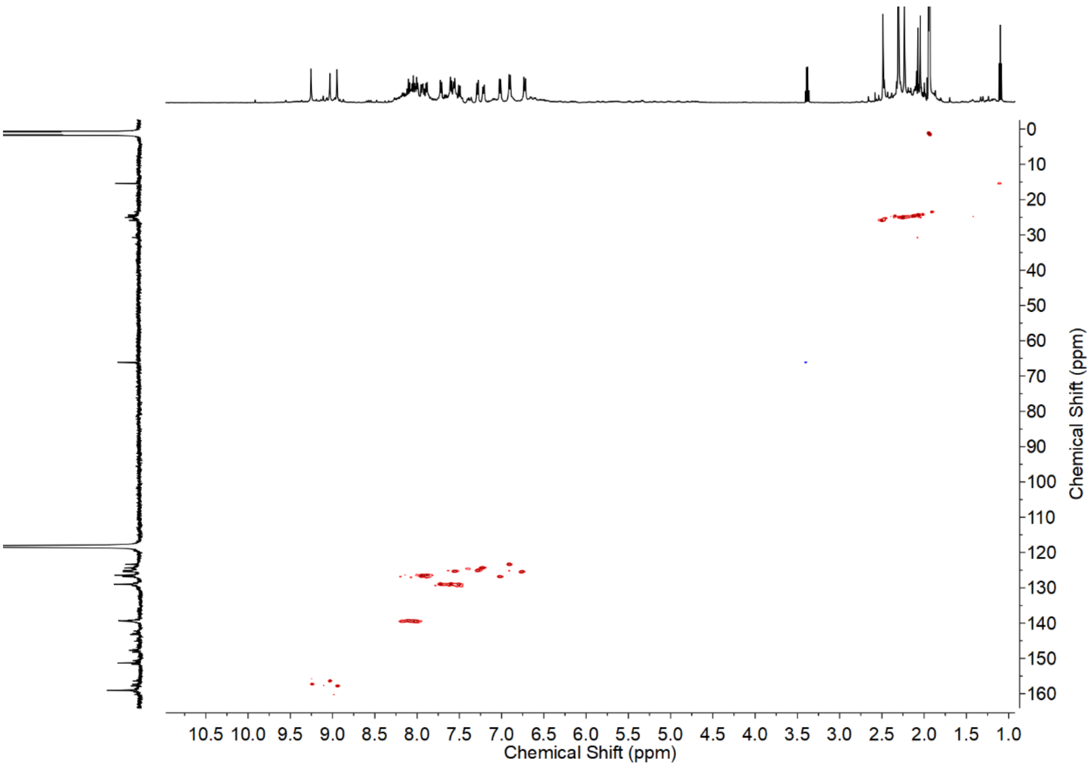

Figure S16: ^{1}H-^{13}C HSQC NMR spectrum of S_4-**1** (500 MHz, CD_3CN, 278 K).

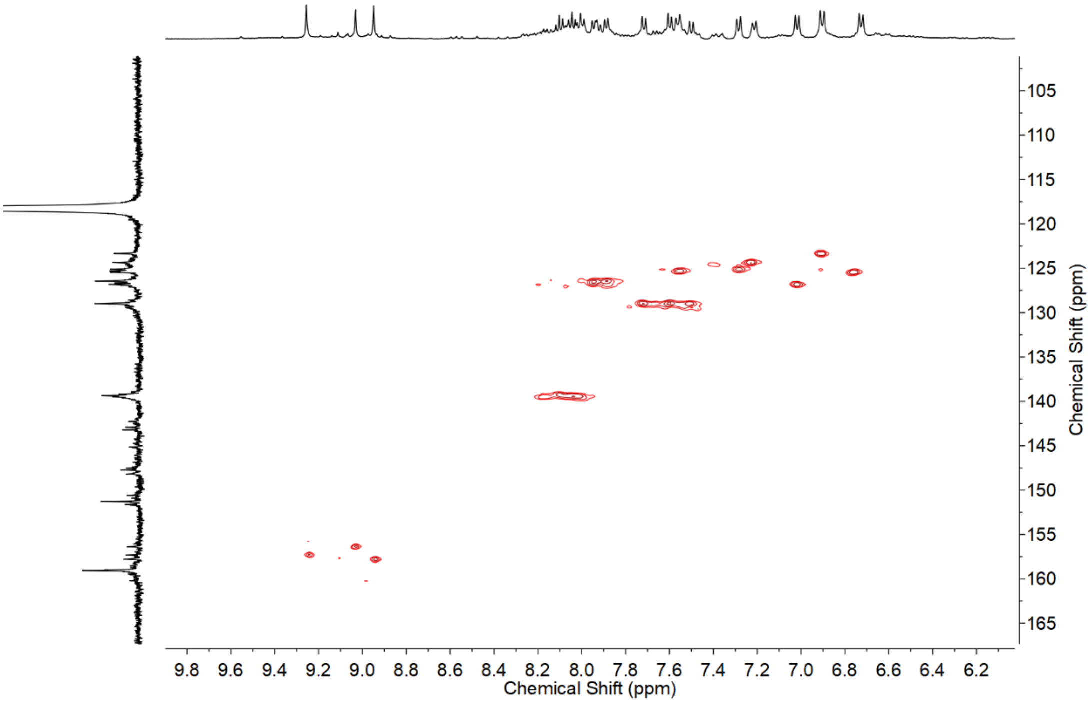

Figure S17: Aromatic region of ^{1}H-^{13}C HSQC NMR spectrum of S_4-**1** (500 MHz, CD_3CN, 278 K).

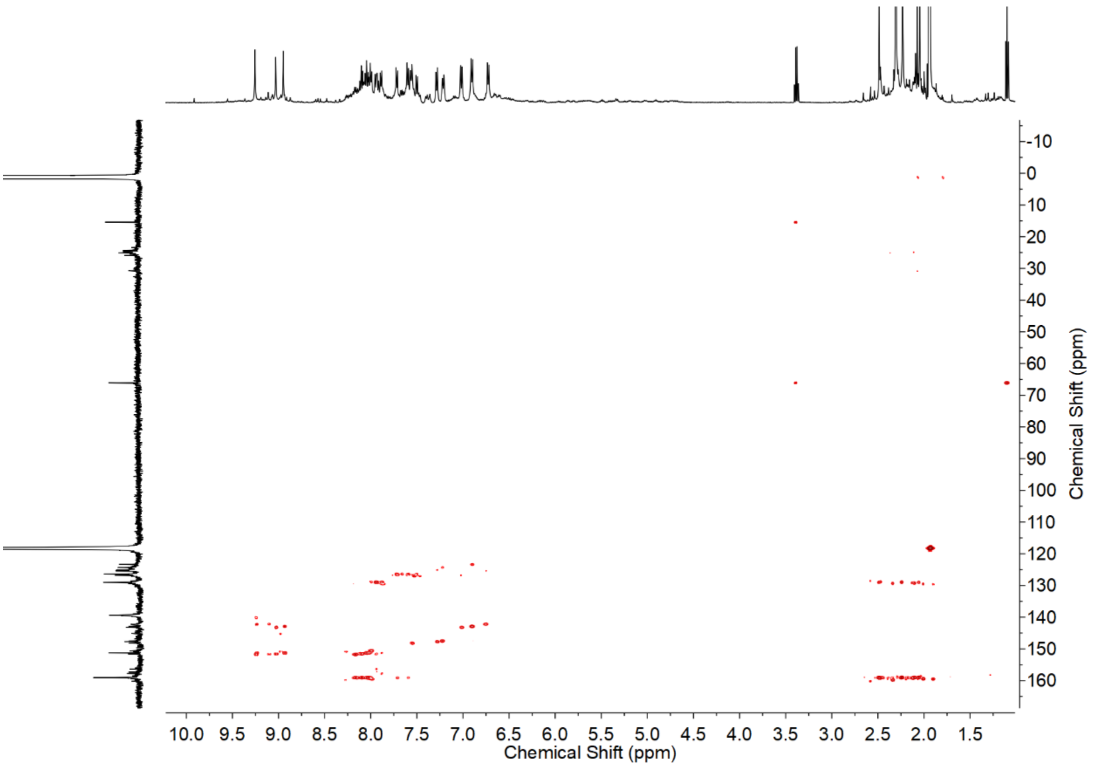

Figure S18: ^{1}H-^{13}C HMBC NMR spectrum of S_4-**1** (500 MHz, CD_3CN, 278 K). The peaks at 1.12 and 3.42 ppm are assigned to residual Et_2O.

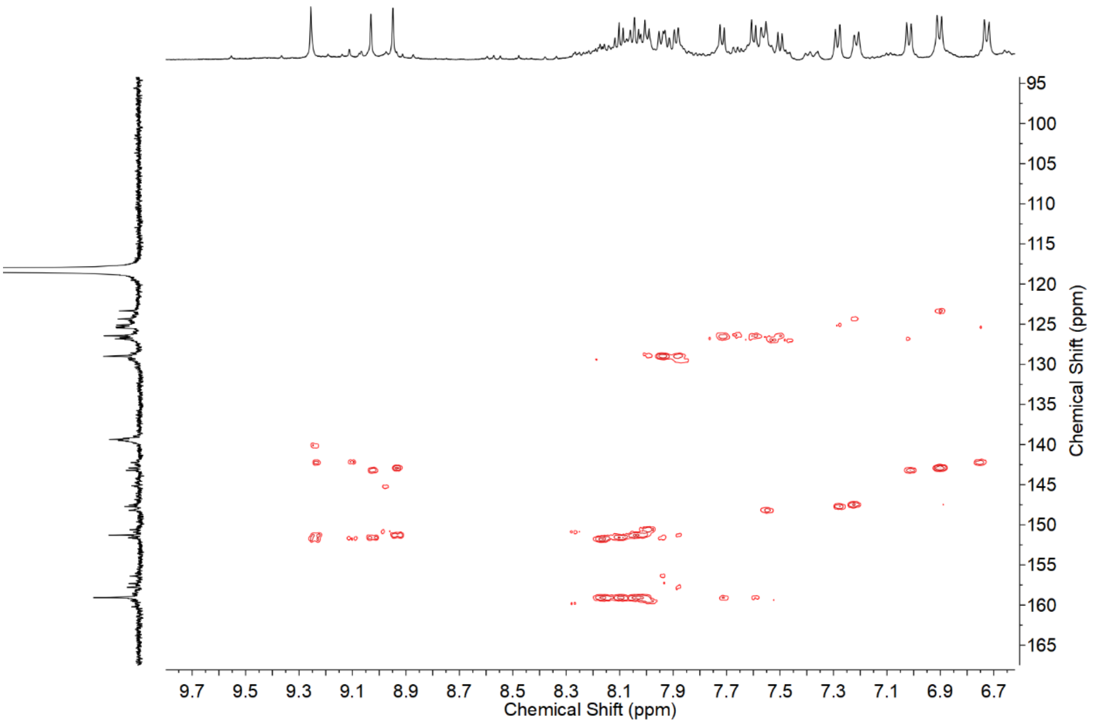

Figure S19: Aromatic region of ^{1}H-^{13}C HMBC NMR spectrum of S_4-**1** (500 MHz, CD_3CN, 278 K).

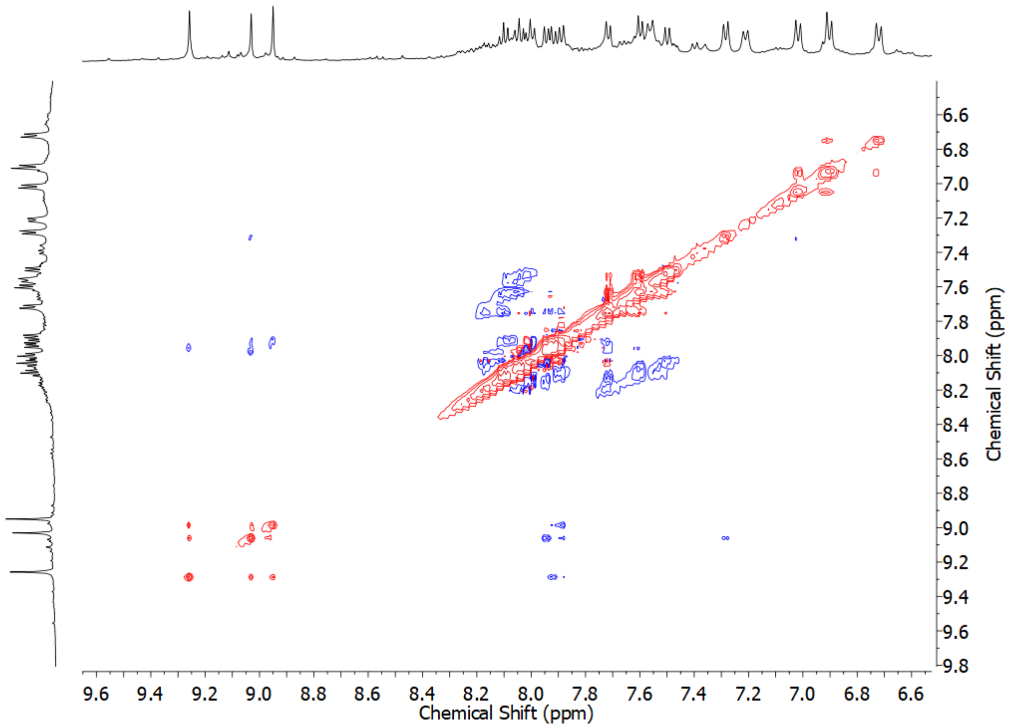

Figure S20: ${}^{1}H$-${}^{1}H$ ROESY NMR spectrum of the aromatic region of S_4-**1** (500 MHz, CD_3CN, 253 K).

S2.3 Solvent-dependent diastereomeric conversion of **1**

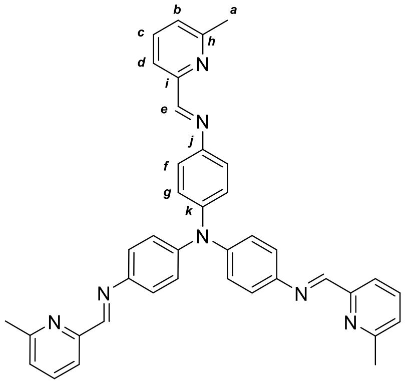

To explore the effect of solvent on the diastereoselectivity of **1**, **1** (20 mg in 1 mL MeCN) was purified through precipitation by the addition of Et_2O (10 mL) and the precipitate was dissolved in DMSO. The equilibration of the system at room temperature was monitored by ^{1}H NMR spectroscopy. The ^{1}H NMR spectrum of **1** was observed to evolve over 9 days at room temperature following its dissolution in DMSO. Beyond 9 days there were no changes in the ^{1}H NMR spectrum, suggesting that the system was approaching equilibrium at a *T*-**1** : S_4-**1** ratio of 4 : 3. The ^{1}H and ^{13}C signals corresponding to the major *T*-**1** diastereomer are assigned below.

δ_H (DMSO-d_6, 700 MHz, 298 K) 9.22 (12H, s, H_e), 8.18 (12H, t, *J* = 7.6 Hz, H_c), 7.97 (12H, d, *J* = 7.7 Hz, H_d), 7.74 (12H, d, *J* = 7.8 Hz, H_b), 7.52 (24H, d, *J* = 8.61 Hz, H_f), 7.01 (24H, d, *J* = 8.7 Hz, H_g), 2.18 (36H, s, H_a).

δ_C (DMSO-d_6, 176 MHz, 298 K) 158.1 (C_h), 157.6 (C_e), 150.1 (C_i), 146.4 (C_k), 142.3 (C_j), 138.9 (C_c), 128.4 (C_b), 126.0 (C_d), 124.3 (C_g), 123.8 (C_f), 24.1 (C_a).

LR-ESI-MS (DMSO) [POS] ([**1**] = $C_{156}H_{132}N_{28}Cu_6(BF_4)_6$) *m/z* = 463.4 $[M\text{-}6BF_4]^{6+}$ (calc. 463.4), 573.5 $[M\text{-}5BF_4]^{5+}$ (calc. 573.4), 738.6 $[M\text{-}4BF_4]^{4+}$ (calc. 738.4), 1013.6 $[M\text{-}3BF_4]^{3+}$ (calc. 1013.5).

HR-ESI-MS (DMSO) [POS] ([**1**] = $C_{156}H_{132}N_{28}Cu_6(BF_4)_6$) *m/z* = 1013.2378 $[M\text{-}3BF_4]^{3+}$ (calc. 1013.2359), 1563.3578 $[M\text{-}2BF_4]^{2+}$ (calc. 1563.3560).

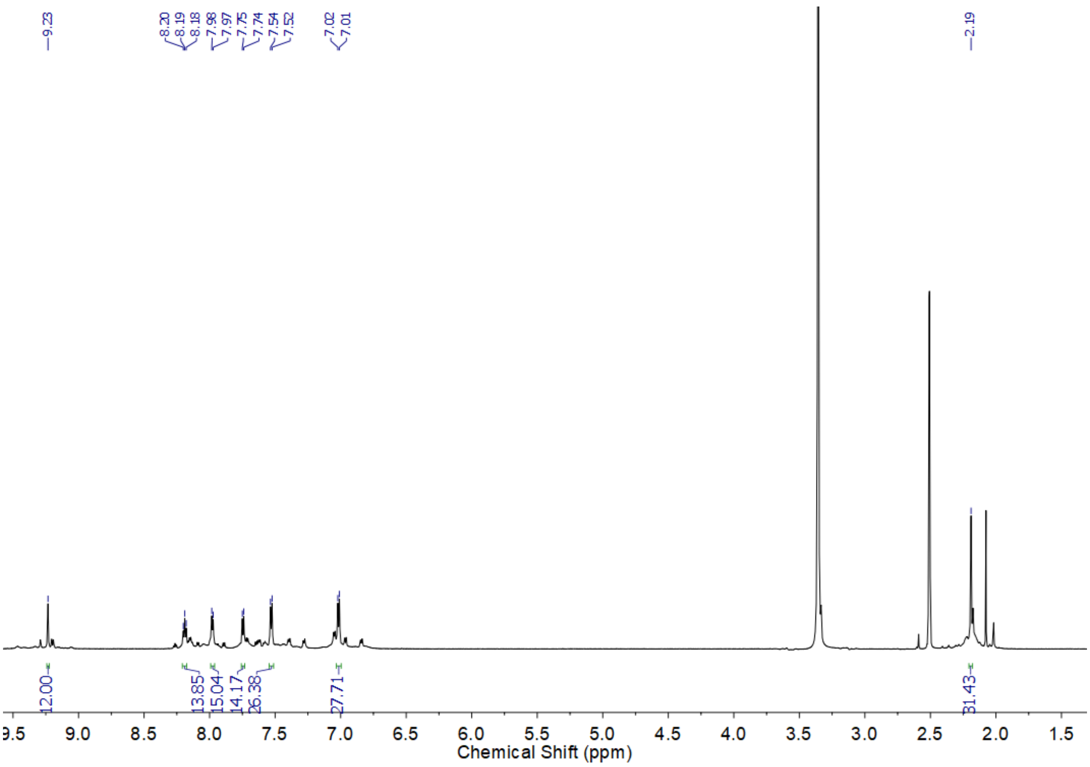

Figure S21: ^{1}H NMR spectrum of 1 (700 MHz, DMSO-d$_6$, 298 K). The major species is assigned to the *T*-**1** diastereomer, with the minor species corresponding to the S_4-**1** diastereomer. For clarity, integrals are only shown for the *T*-**1** diastereomer.

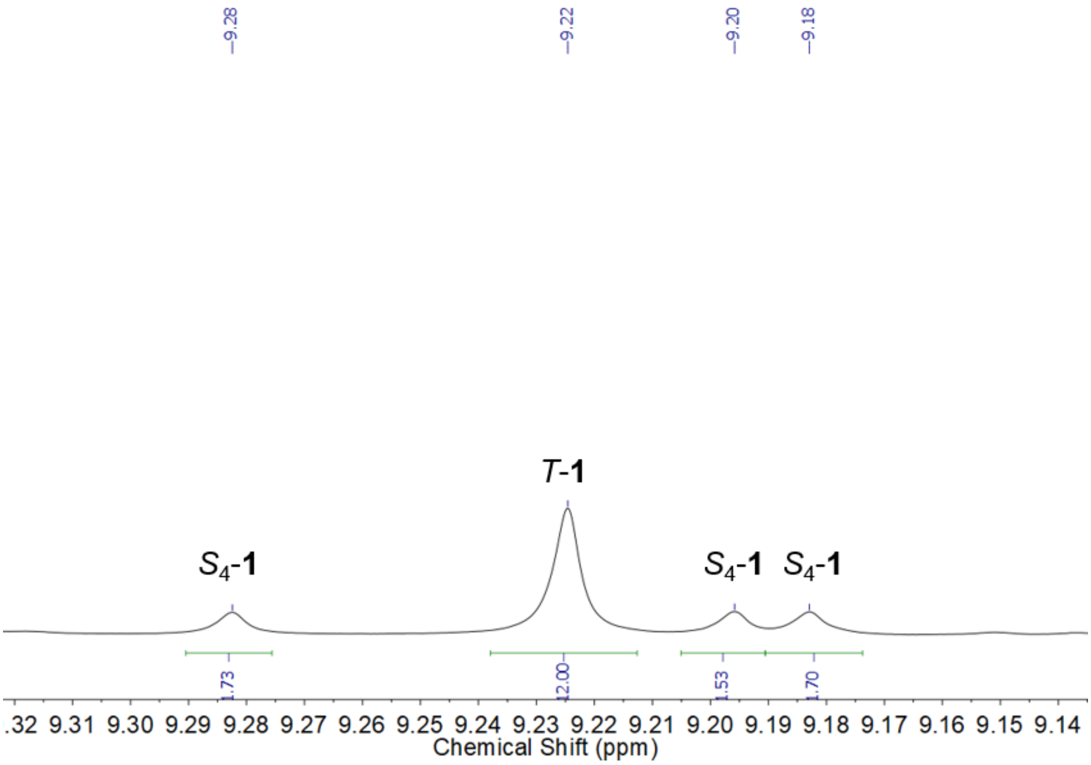

Figure S22: ^{1}H NMR spectrum of the imine region of **1** (700 MHz, DMSO-d$_6$, 298 K). The integrals of the *T*-**1** (major) and S_4-**1** (minor) species are shown. Deconvolution of the imine signals gives the equilibrium proportion of *T*-**1** : S_4-**1** as 4 : 3.

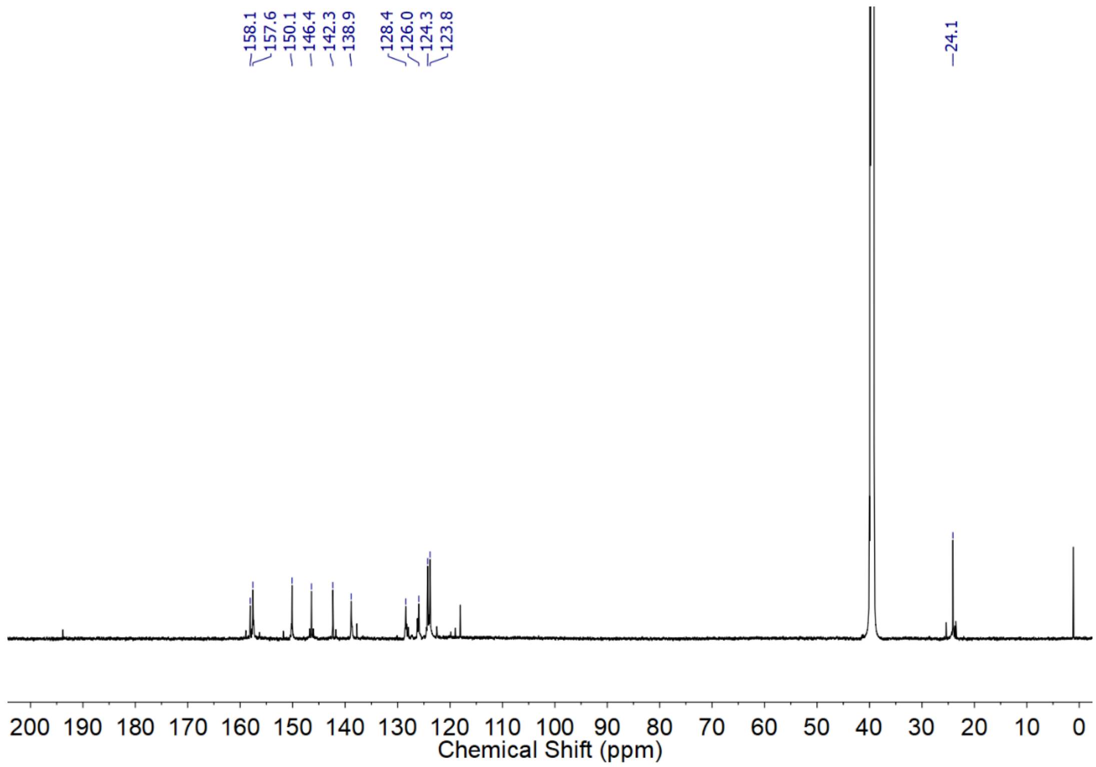

Figure S23: ^{13}C NMR spectrum of **1** (176 MHz, DMSO-d_6, 298 K).

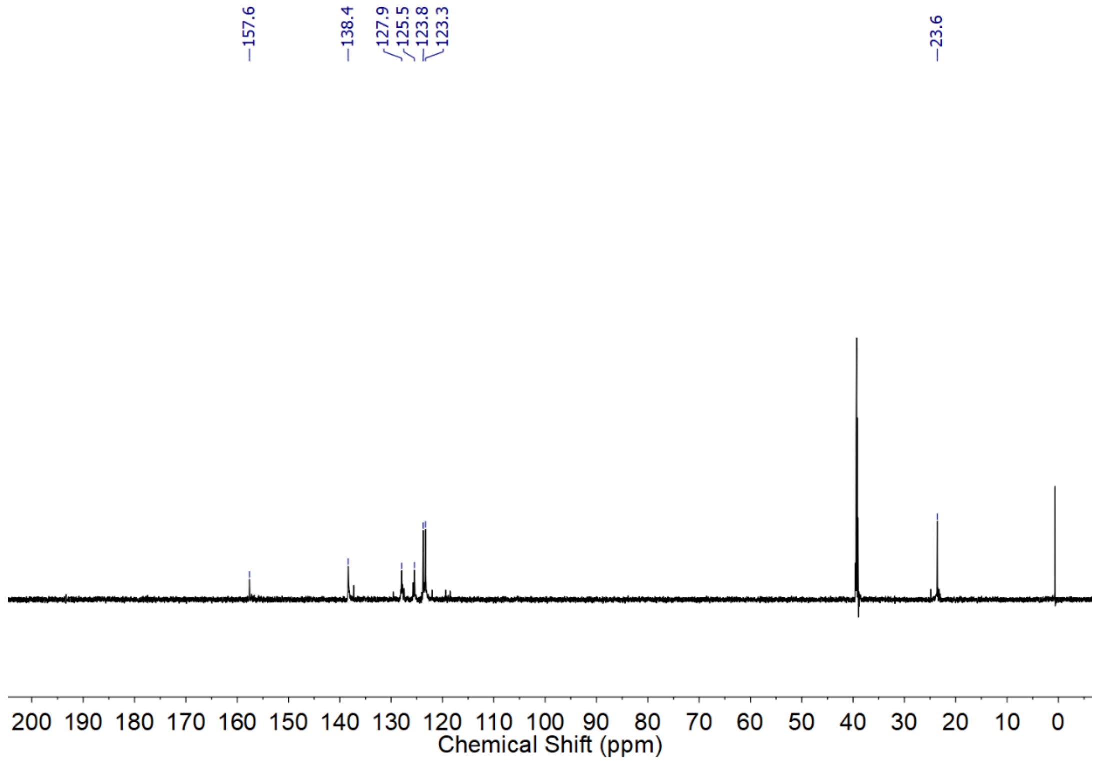

Figure S24: DEPT-135 spectrum of the aromatic region of **1** (176 MHz, DMSO-d_6, 298 K).

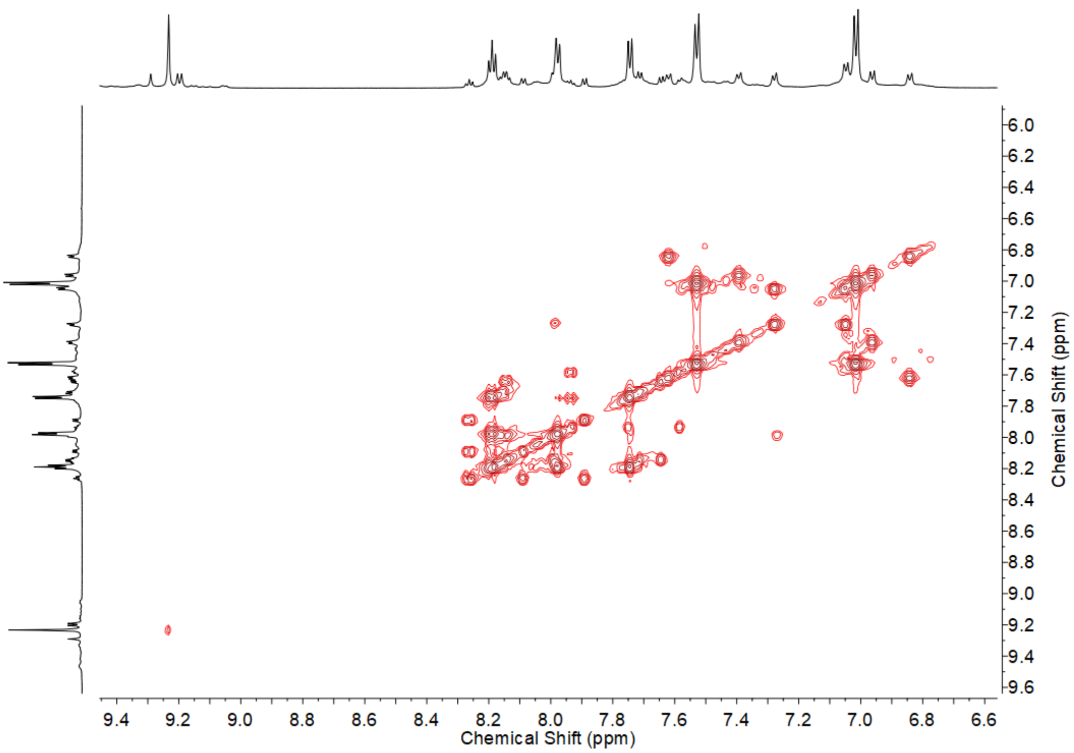

Figure S25: ^{1}H-^{1}H DQF-COSY NMR spectrum of the aromatic region of **1** (700 MHz, DMSO-d_6, 298 K).

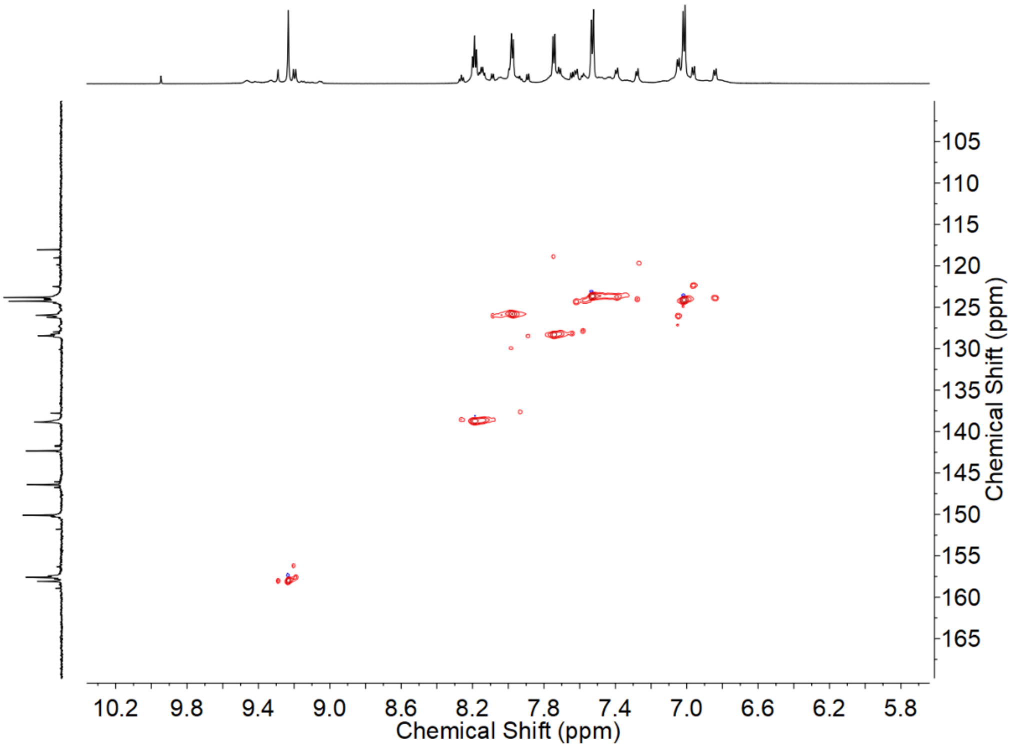

Figure S26: ^{1}H-^{13}C HSQC NMR spectrum of the aromatic region of **1** (700 MHz, DMSO-d_6, 298 K).

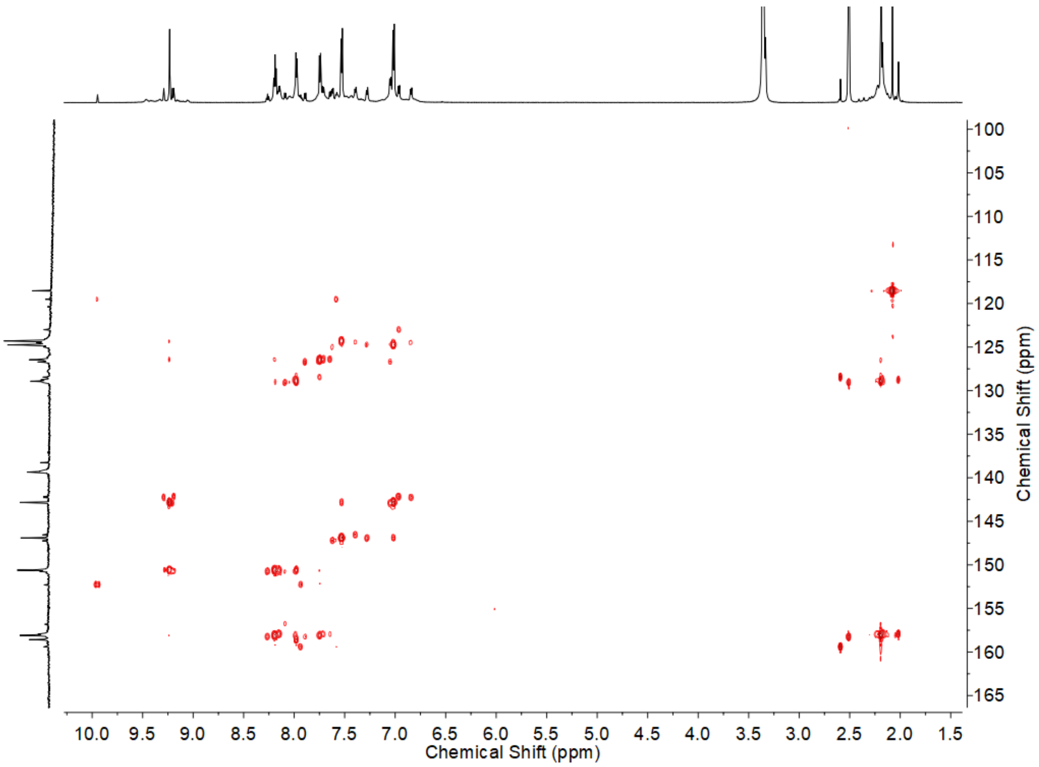

Figure S27: ^{1}H-^{13}C HMBC NMR spectrum of **1** (700 MHz, DMSO-d_6, 298 K).

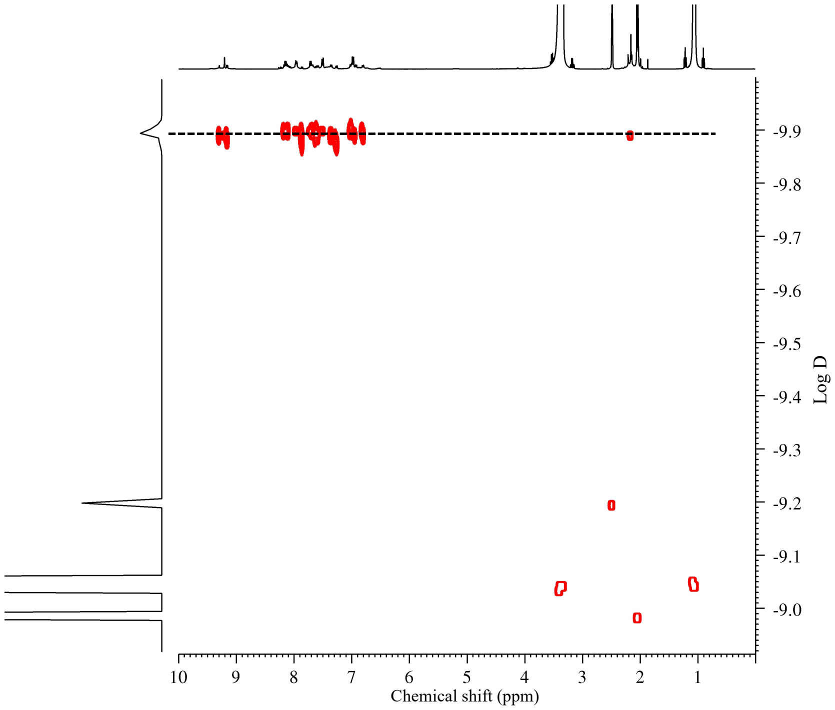

Figure S28: ^{1}H DOSY NMR spectrum of **1** (400 MHz, DMSO-d_6, 298 K). A diffusion coefficient of 1.30×10^{-10} m^2s^{-1} was obtained, corresponding to a solvodynamic radius of 8.4 Å.

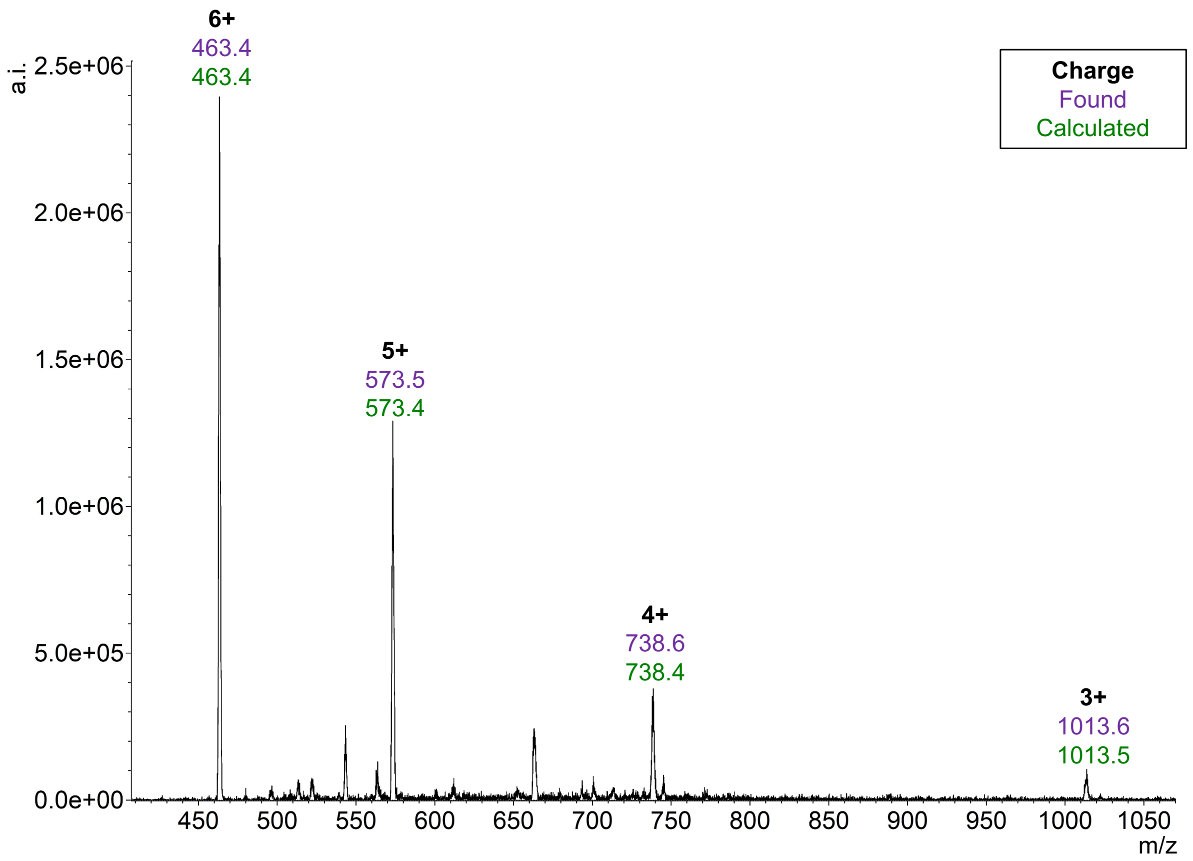

Figure S29: Low-resolution ESI-mass spectrum (DMSO) of **1** $[Cu_6L_4](BF_4)_6$ $[M-nBF_4]^{n+}$.

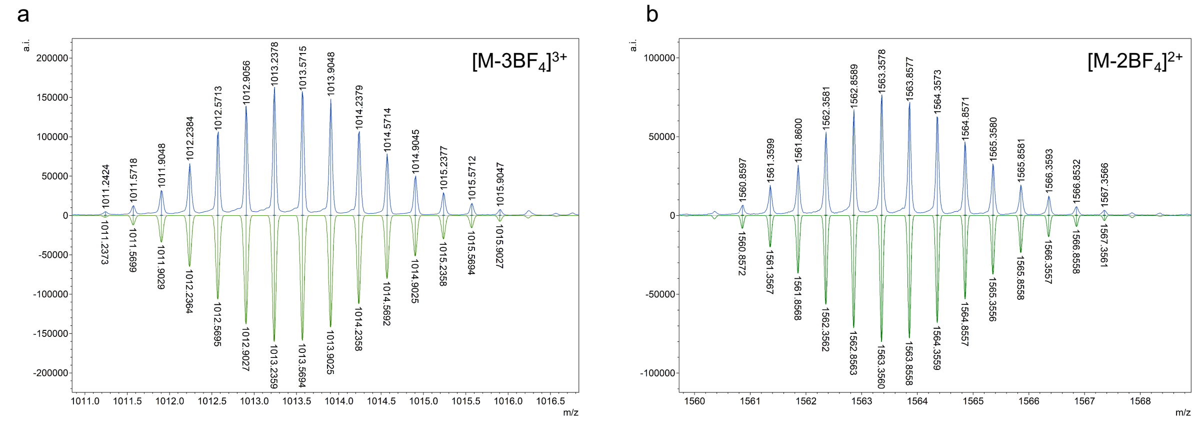

Figure S30: High-resolution ESI mass spectrum of **1**. Experimental (blue) and theoretical (green) peaks for (a) $[M-3BF_4]^{3+}$; (b) $[M-2BF_4]^{2+}$.

S3. Diastereoinduction with tetrahedral guests

Host **1** bound tetrahedral guests **G1**, **G2** and **G3**. ESI-MS spectra showed a series of signals corresponding to **1** binding a single equivalent of **G1** or **G3** (Figs. S43–S44 and S70–S71). NMR and isothermal titration calorimetry (ITC) titrations show an initial strong, internal binding event for tetrahedral anionic guest **G2**, and further weaker binding attributed to external electrostatic interactions. This is corroborated by ESI-MS for **G2⊂1** (Figs. S56–S57), where **1** is observed to associate with 2 or 3 equivalents of **G2** in the gas phase.

S3.1 Characterization of **G1**⊂*T*-**1**

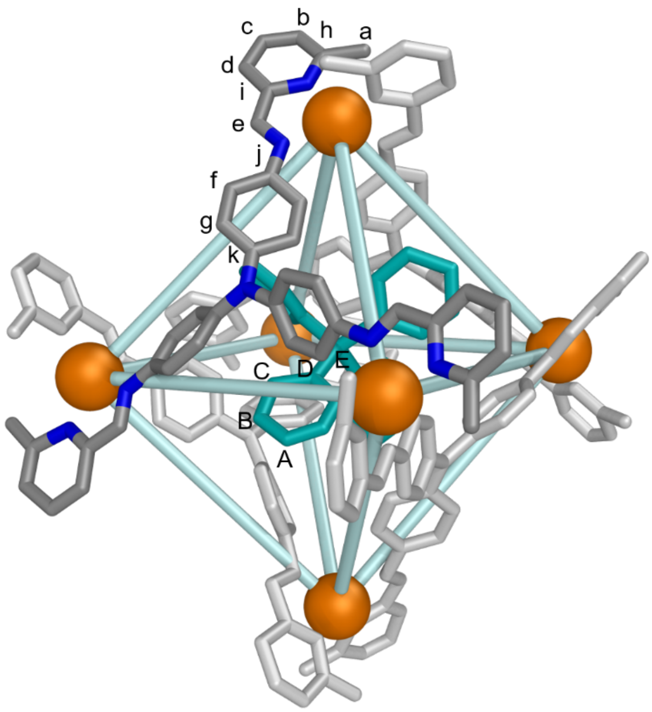

G1⊂*T*-**1**

δ_H (500 MHz, CD_3CN, 298 K) 9.00 (s, 12H, H_e), 8.10 (t, 12H, *J* = 7.8 Hz, H_c), 7.93 (d, 12H, *J* = 7.6 Hz, H_d), 7.64 (d, 12H, *J* = 7.8 Hz, H_b), 7.27 (d, 24H, *J* = 8.8 Hz, H_f), 6.78 (t, 4H, *J* = 7.3 Hz, H_A), 6.52 (d, 24H, *J* = 8.8 Hz, H_g), 6.38 (d, 8H, *J* = 7.7 Hz, H_C), 5.96 (t, 8H, *J* = 7.8 Hz, H_B), 2.26 (s, 36H, H_a).

δ_c (176 MHz, CD_3CN, 298 K) 159.2 (C_h), 158.7 (C_e), 151.5 (C_i), 147.6 (C_j), 146.8 (C_D), 143.5 (C_k), 139.6 (C_c), 132.5 (C_C), 129.2 (C_b), 127.5 (C_B), 127.1 (C_A), 126.7 (C_d), 125.3 (C_g), 124.5 (C_f), 65.4 (C_E), 25.1 (C_a).

LR-ESI-MS [POS] ([**G1**⊂**1**] = $C_{156}H_{132}N_{28}Cu_6(C_{25}H_{20})(BF_4)_6$) *m/z* = 516.8 $[M\text{-}6BF_4]^{6+}$ (calc. 516.8) 637.5 $[M\text{-}5BF_4]^{5+}$ (calc. 637.5), 818.5 $[M\text{-}4BF_4]^{4+}$ (calc. 818.6), 1120.3 $[M\text{-}3BF_4]^{3+}$ (calc. 1120.3).

HR-ESI-MS [POS] ([**G1**⊂**1**] = $C_{156}H_{132}N_{28}Cu_6(C_{25}H_{20})(BF_4)_6$) *m/z* = 637.3677 $[M\text{-}5BF_4]^{5+}$ (calc. 637.3714), 818.4600 $[M\text{-}4BF_4]^{4+}$ (calc. 818.4652), 1120.2847 $[M\text{-}3BF_4]^{3+}$ (calc. 1120.2883).

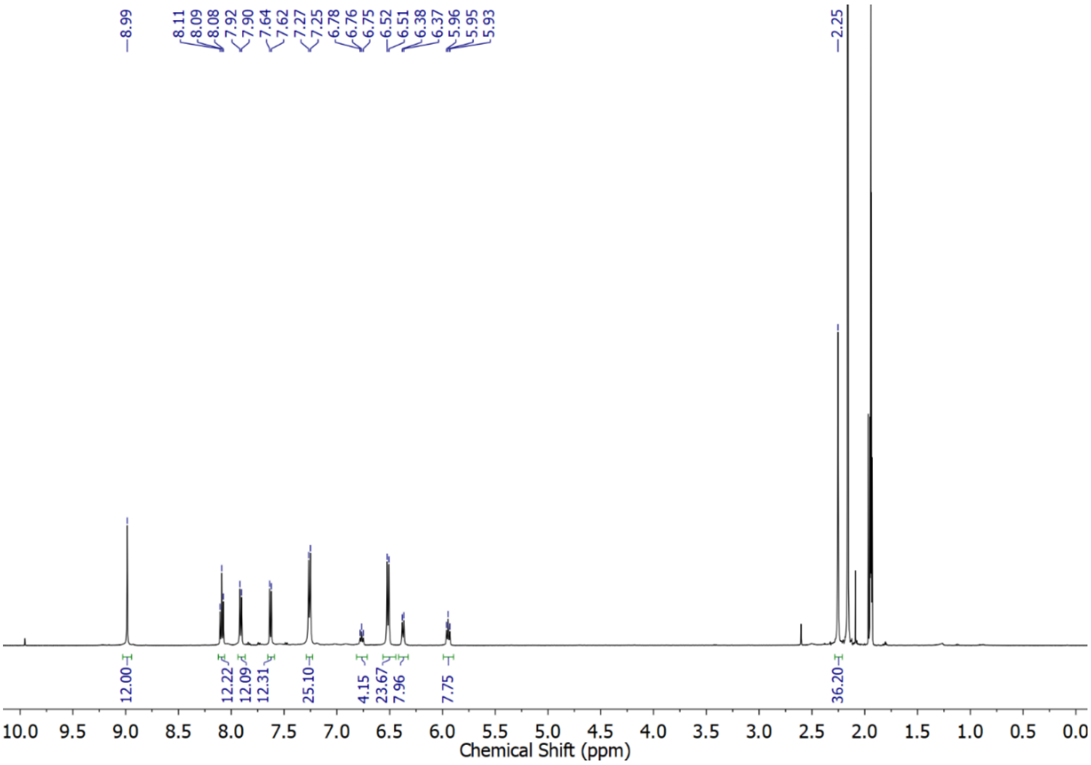

Figure S31: ^{1}H NMR spectrum of **G1**⊂*T*-**1** (500 MHz, CD_3CN, 298 K). The minor species with a peak near 10 ppm corresponds to excess aldehyde.

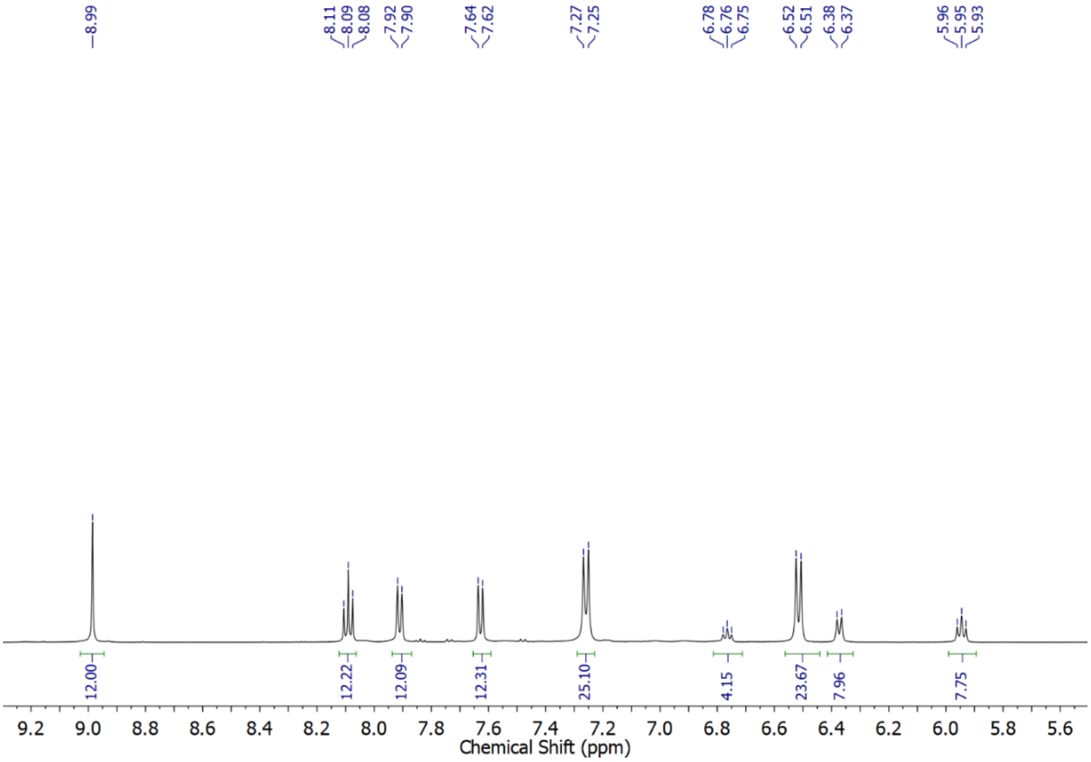

Figure S32: The aromatic region of the ^{1}H NMR spectrum of **G1**⊂*T*-**1** (500 MHz, CD_3CN, 298 K).

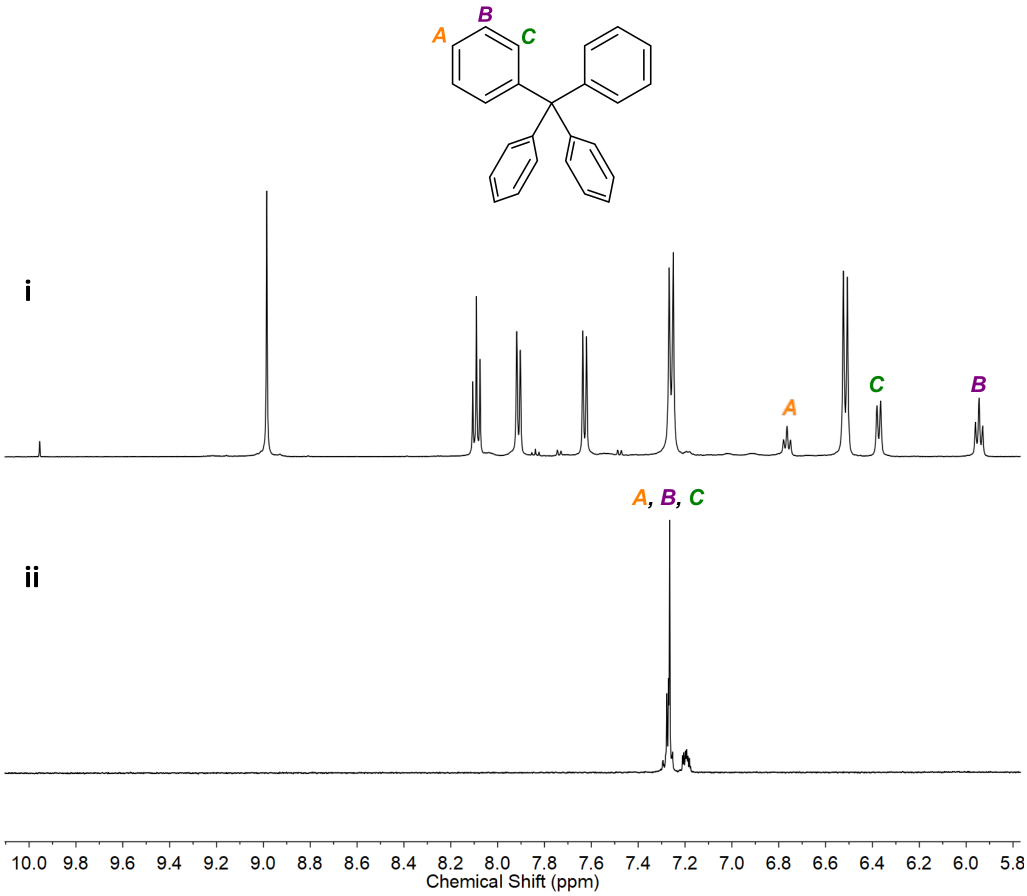

Figure S33: Stacked ^{1}H NMR spectra of: **i** the aromatic region of **G1**⊂*T*-**1** and **ii** free **G1** (500 MHz, CD_3CN, 298 K). Protons A, B and C of **G1** are labeled in both spectra to show the change in chemical shift of each proton signal upon encapsulation.

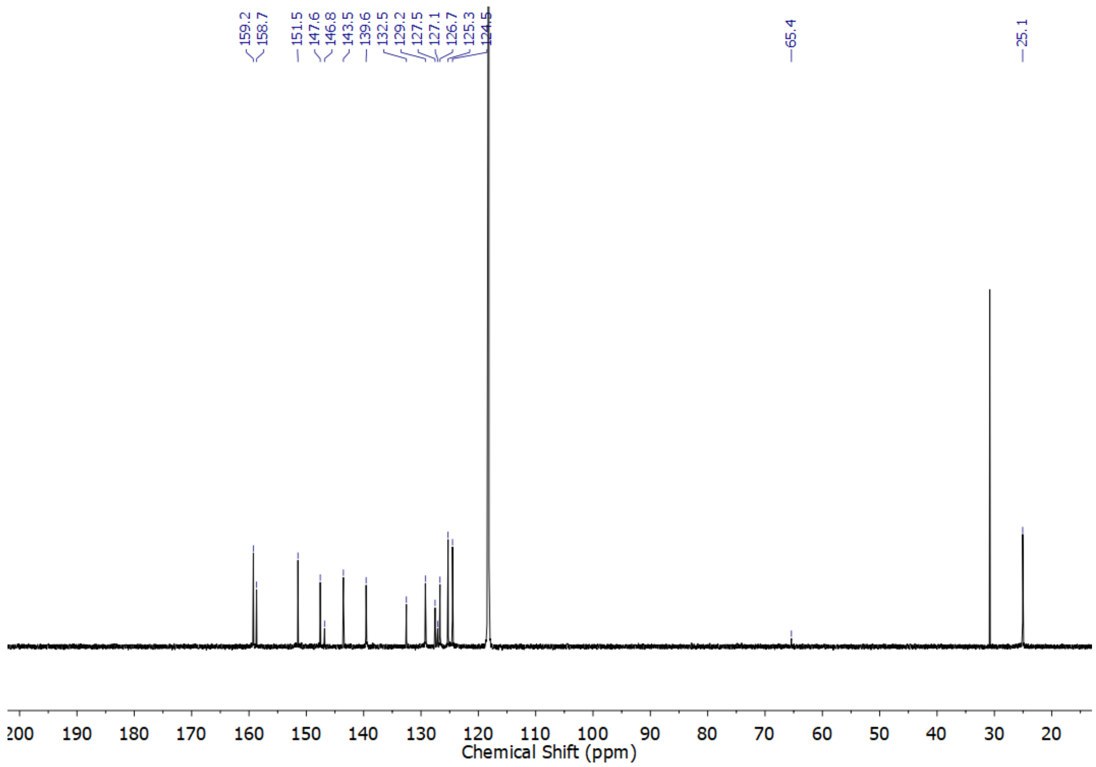

Figure S34: ^{13}C NMR spectrum of **G1**⊂*T*-**1** (176 MHz, CD_3CN, 298 K). The signal at 30.9 ppm is assigned to residual acetone.

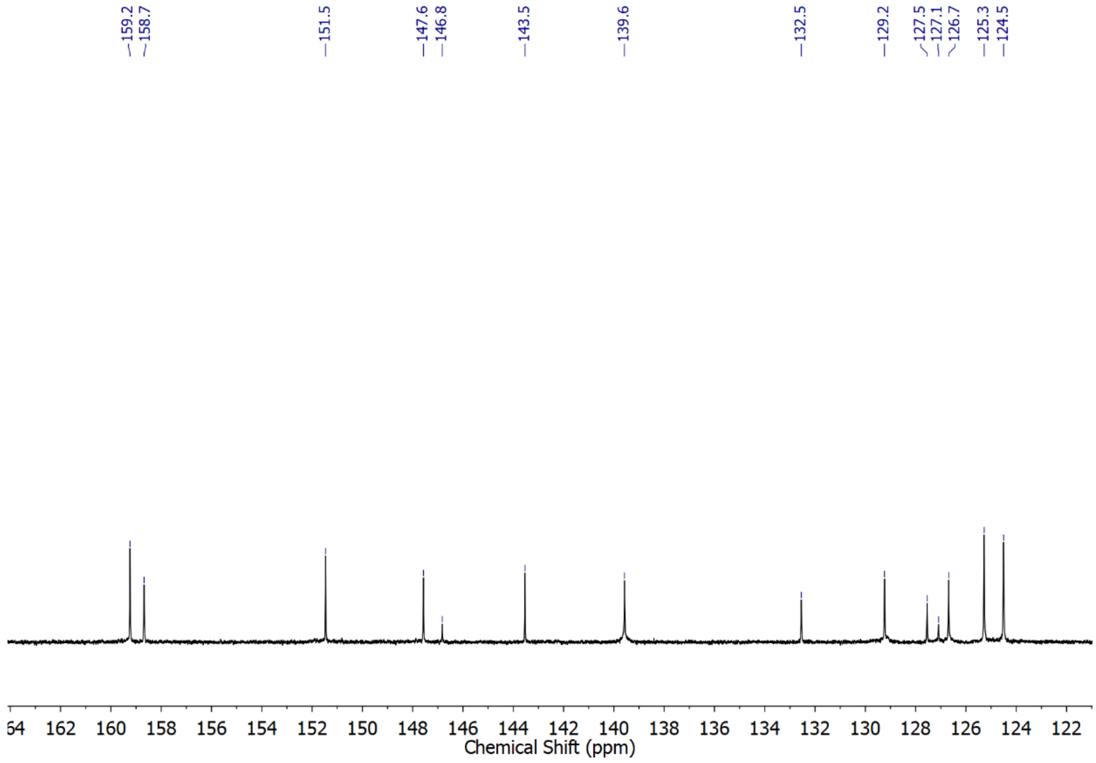

Figure S35: The aromatic region of the ^{13}C NMR spectrum of **G1**⊂*T*-**1** (176 MHz, CD_3CN, 298 K).

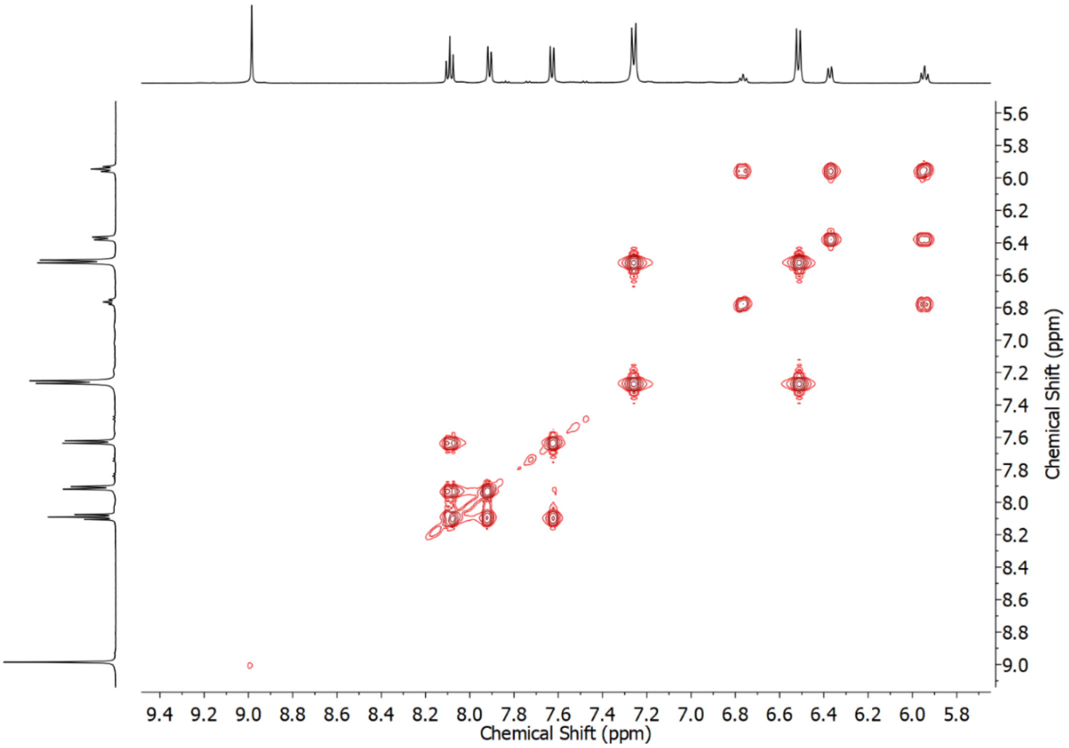

Figure S36: The aromatic region of the ^{1}H-^{1}H DQF-COSY NMR spectrum of **G1**⊂*T*-**1** (500 MHz, CD_3CN, 298 K).

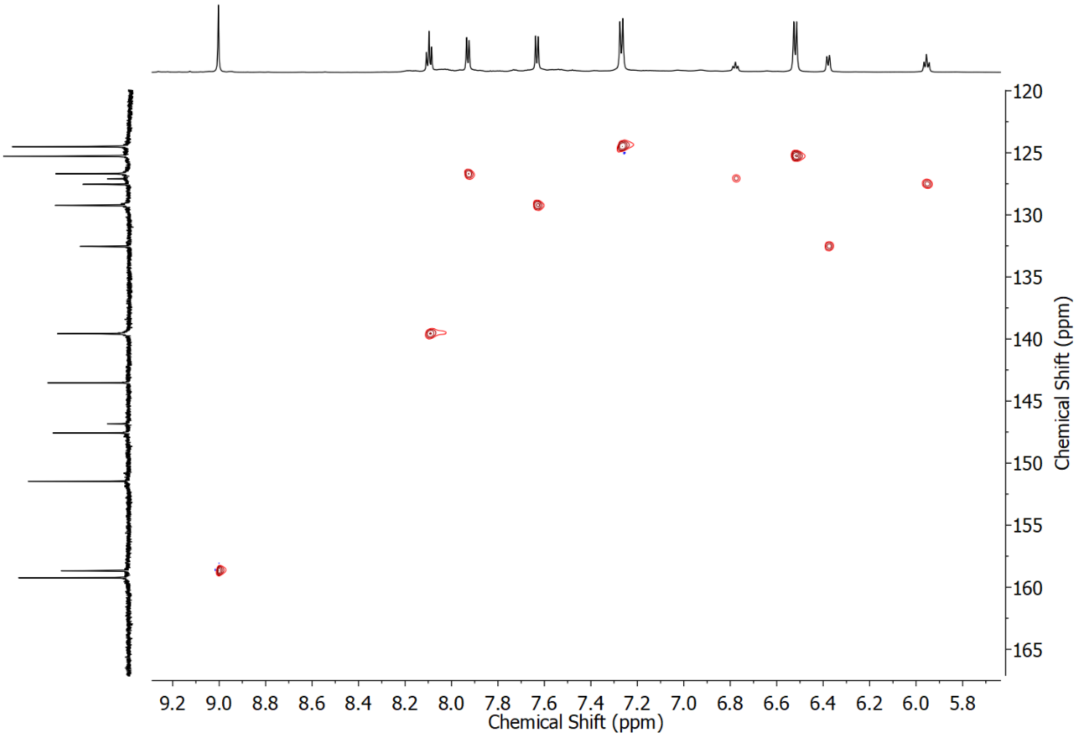

Figure 37: The aromatic region of the ^{1}H-^{13}C HSQC NMR spectrum of **G1**⊂*T*-**1** (700 MHz, CD_3CN, 298 K).

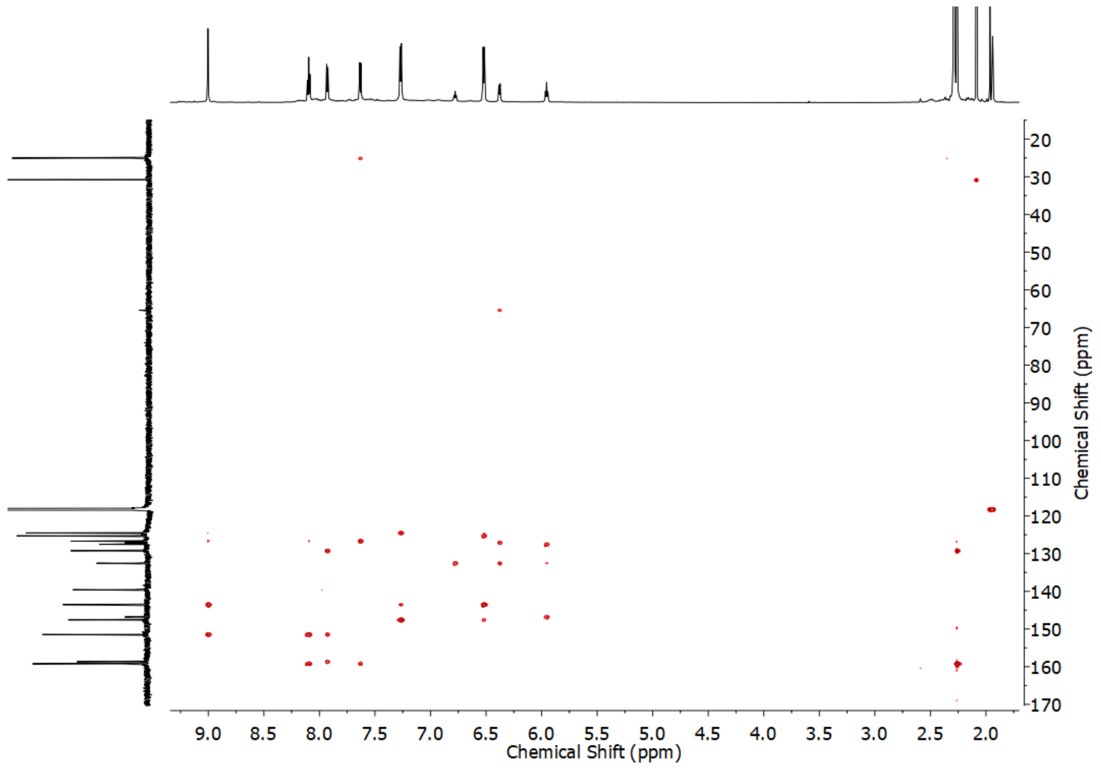

Figure S38: ^{1}H-^{13}C HMBC NMR spectrum of **G1**⊂*T*-**1** (700 MHz, CD_3CN, 298 K).

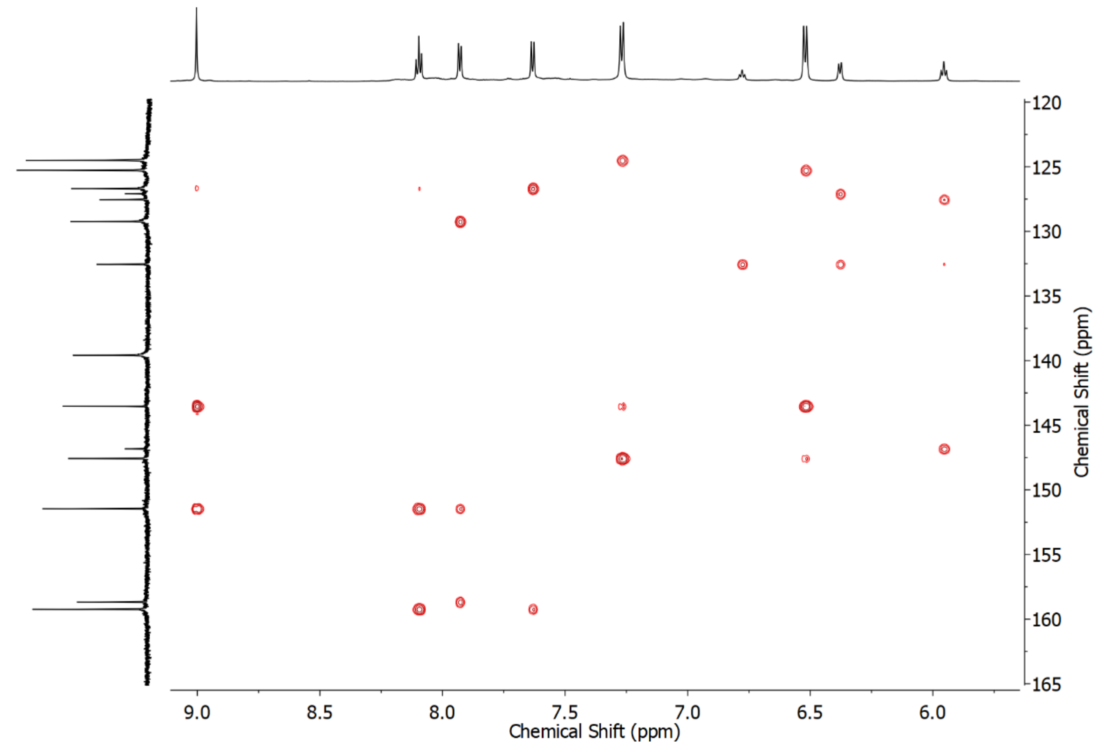

Figure S39: The aromatic region of the ^{1}H-^{13}C HMBC NMR spectrum of **G1**⊂*T*-**1** (700 MHz, CD_3CN, 298 K).

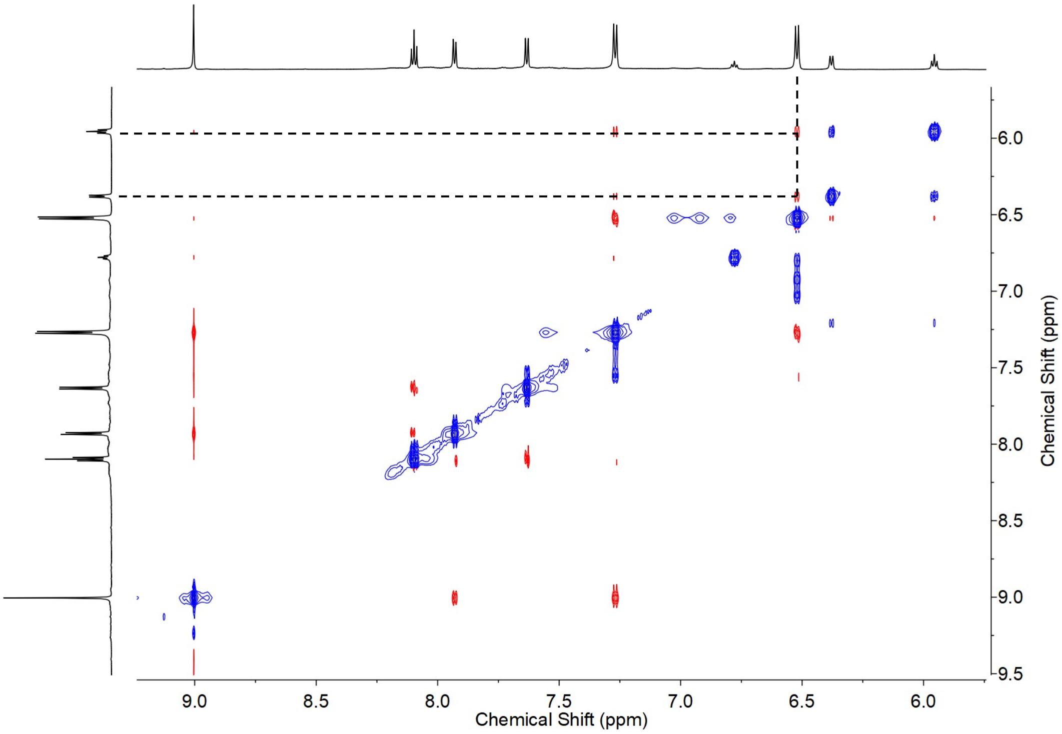

Figure S40: ^1H-^1H ROESY NMR spectrum of **G1**⊂*T*-**1** (700 MHz, CD_3CN, 298 K). ^1H-^1H through-space correlations between *T*-**1** and **G1** are shown by dashed lines.

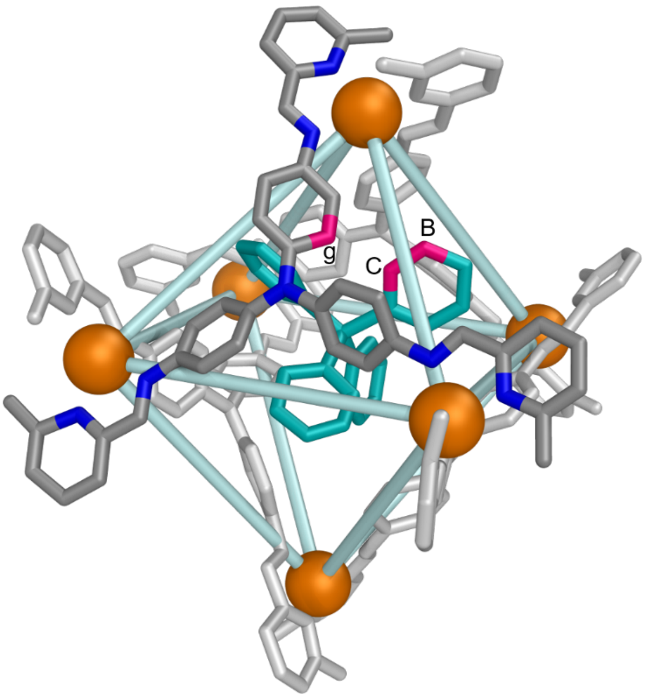

G1⊂*T*-**1**

Figure S41: ^1H-^1H ROESY correlations between *T*-**1** and **G1** highlighted in pink.

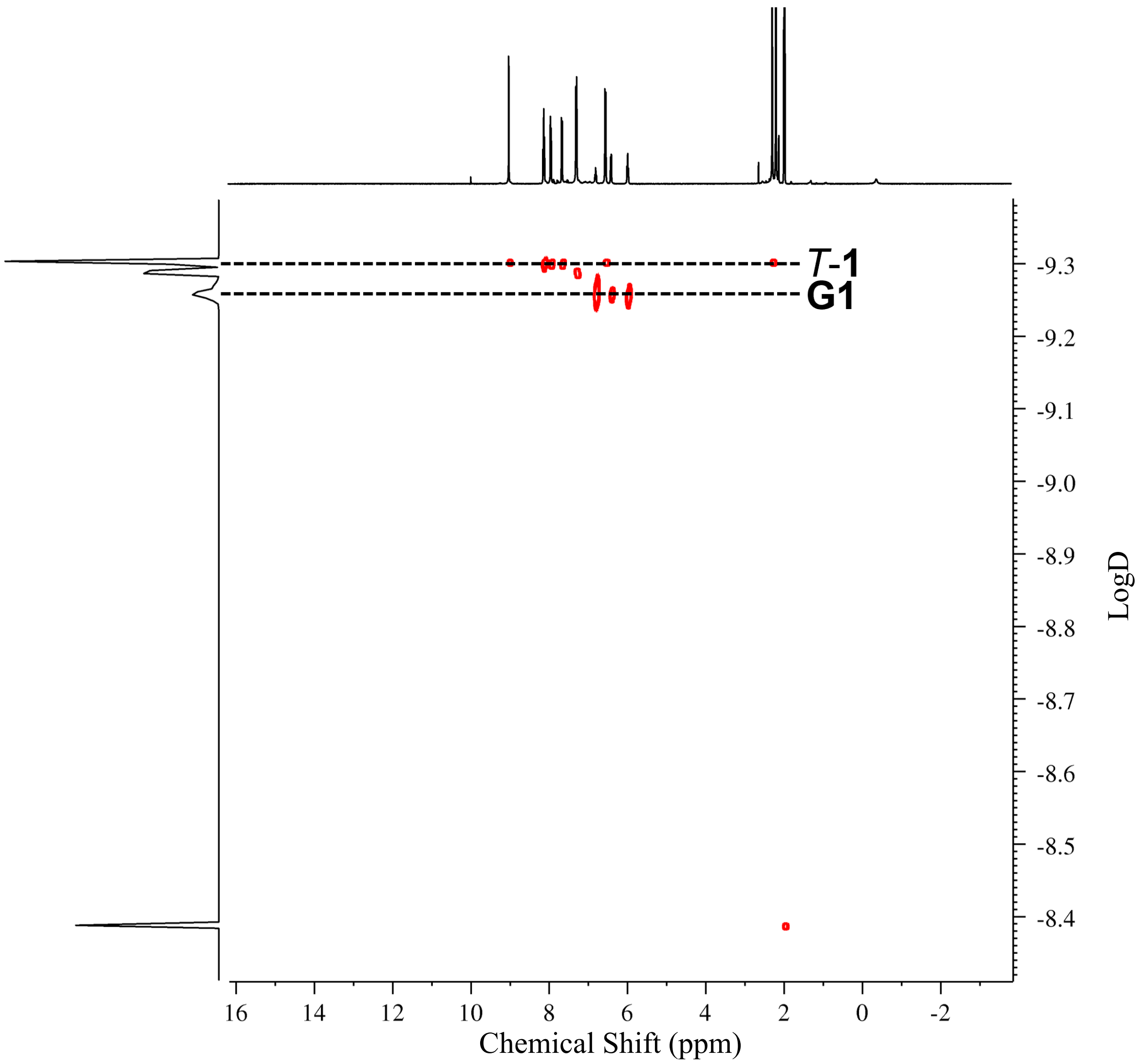

Figure S42: ^{1}H DOSY NMR spectrum of **G1**⊂*T*-**1** (400 MHz, CD_3CN, 298 K). The diffusion coefficient for **G1**⊂*T*-**1** is 5.01×10^{-10} m^2s^{-1}, corresponding to a solvodynamic radius of 12.8 Å. Free and bound **G1** are in fast exchange on the NMR timescale, giving a diffusion coefficient for **G1** averaged between those for free **G1** and **G1**⊂*T*-**1**.

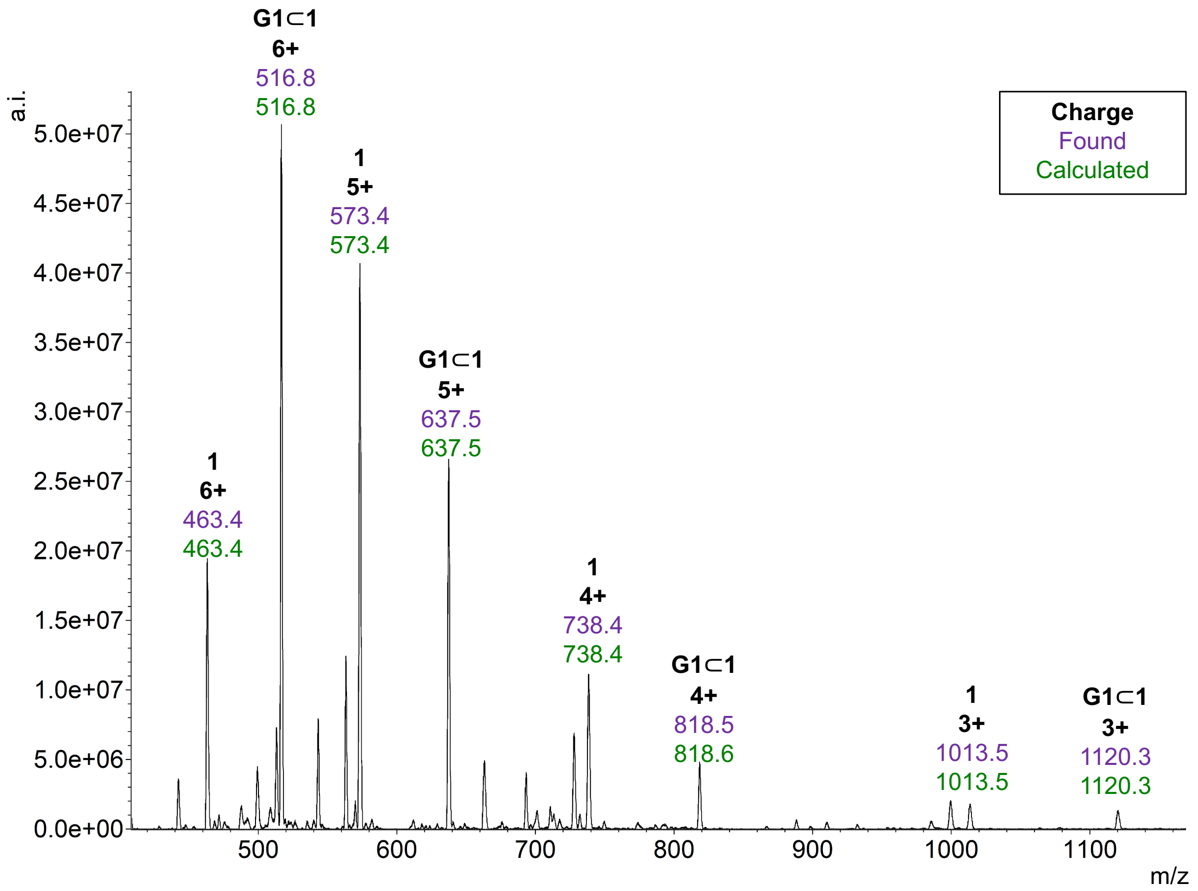

Figure S43: Low resolution ESI-MS spectrum for **G1⊂1**. Signals corresponding to **G1⊂1** and empty **1** are labeled.

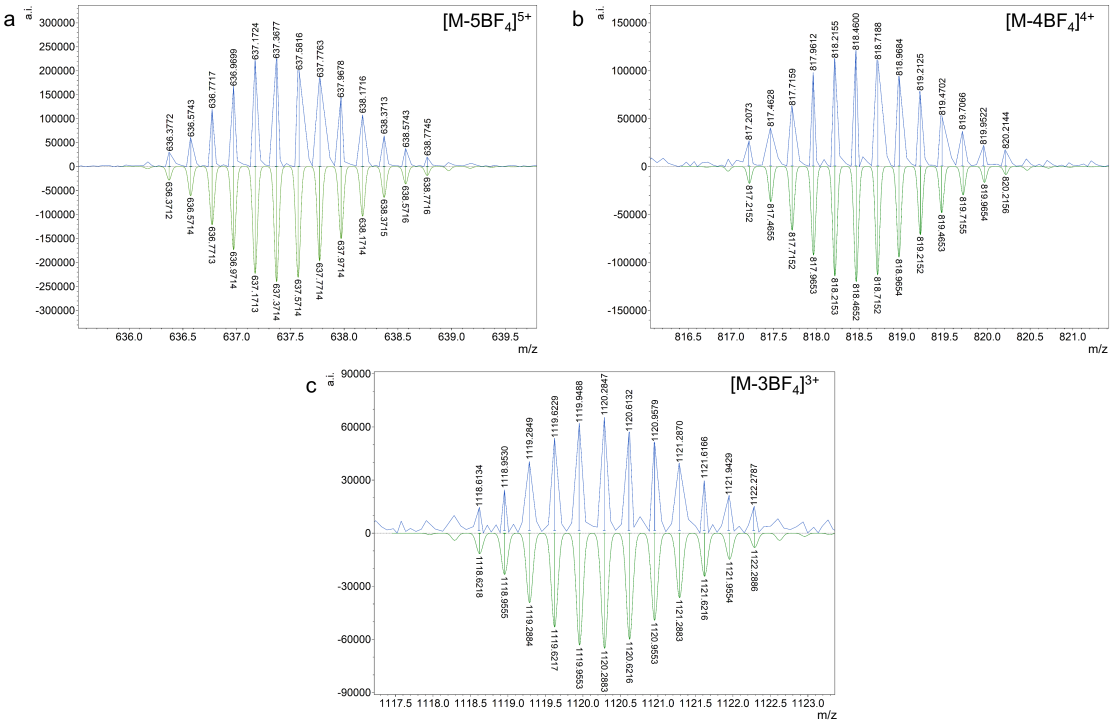

Figure S44: High resolution ESI-mass spectrum of **G1⊂1**. Experimental (blue) and theoretical (green) peaks for (a) $[M\text{-}5BF_4)]^{5+}$; (b) $[M\text{-}4BF_4]^{4+}$; (c) $[M\text{-}3BF_4]^{3+}$.

S3.2 Characterization of **G2**⊂*T*-**1**

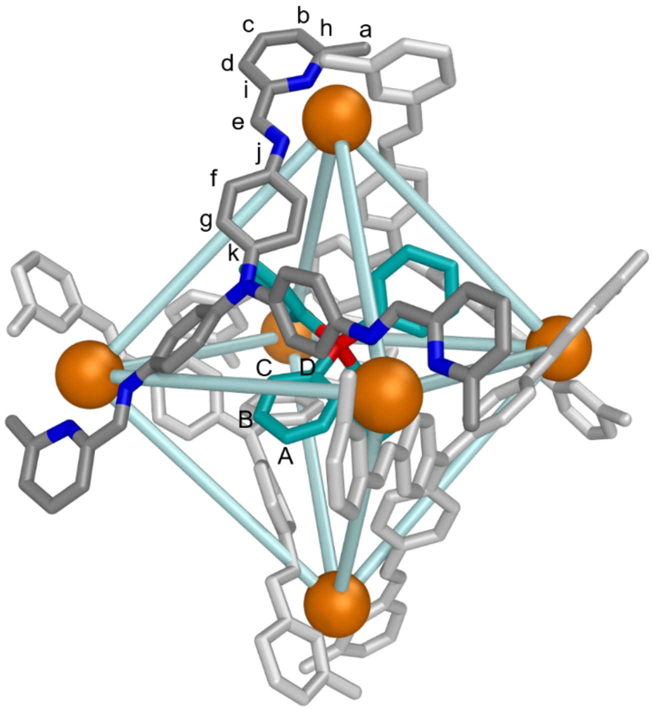

G2⊂*T*-**1**

δ_H (500 MHz, CD_3CN, 298 K) 8.98 (s, 12H, H_e), 8.06 (t, 12H, *J* = 7.8 Hz, H_c), 7.85 (d, 12H, *J* = 7.6 Hz, H_d), 7.60 (d, 12H, *J* = 7.8 Hz, H_b), 7.20 (d, 24H, *J* = 8.4 Hz, H_f), 6.51 (br, 12H, H_C), 6.47 (d, 24H, *J* = 7.4 Hz, H_g), 6.28 (t, 12H, *J* = 7.3 Hz, H_A), 5.56 (t, 24H, *J* = 7.3 Hz, H_B), 2.21 (s, 36H, H_a).

δ_C (126 MHz, CD_3CN, 298 K) 159.3 (C_h), 158.0 (C_e), 151.6 (C_i), 148.5 (C_k), 143.4 (C_j), 139.6 (C_c), 138.2 (C_C), 129.2 (C_b), 126.6 (C_d), 126.1 (C_g), 125.2 (C_B), 124.5 (C_f), 121.8 (C_A), 25.1 (C_a). The signal corresponding to quaternary carbon C_D was too low in intensity to be observed.

LR-ESI-MS [POS] ([**G2**⊂*T*-**1**] = [($C_{156}H_{132}N_{28}Cu_6$)($C_{24}H_{20}B$)(BF_4)$_5$] *m/z* = 620.0 [*T*-**1**(**G2**)]$^{5+}$ (calc. 619.9), 796.6 [*T*-**1**(**G2**)(BF_4)]$^{4+}$ (calc. 796.6), 1091.2 [*T*-**1**(**G2**)(BF_4)$_2$]$^{3+}$ (calc. 1091.0).

HR-ESI-MS [POS] ([**G2**⊂*T*-**1**] = [($C_{156}H_{132}N_{28}Cu_6$)($C_{24}H_{20}B$)(BF_4)$_5$] *m/z* = 619.7709 [M-5BF$_4$]$^{5+}$ (calc. 619.7728), 796.4658 [M-4BF$_4$]$^{4+}$ (calc. 796.4668), 1090.9538 [M-3BF$_4$)$_2$]$^{3+}$ (calc. 1090.9571), 1679.9262 [M-2BF$_4$]$^{2+}$ (calc. 1679.9380).

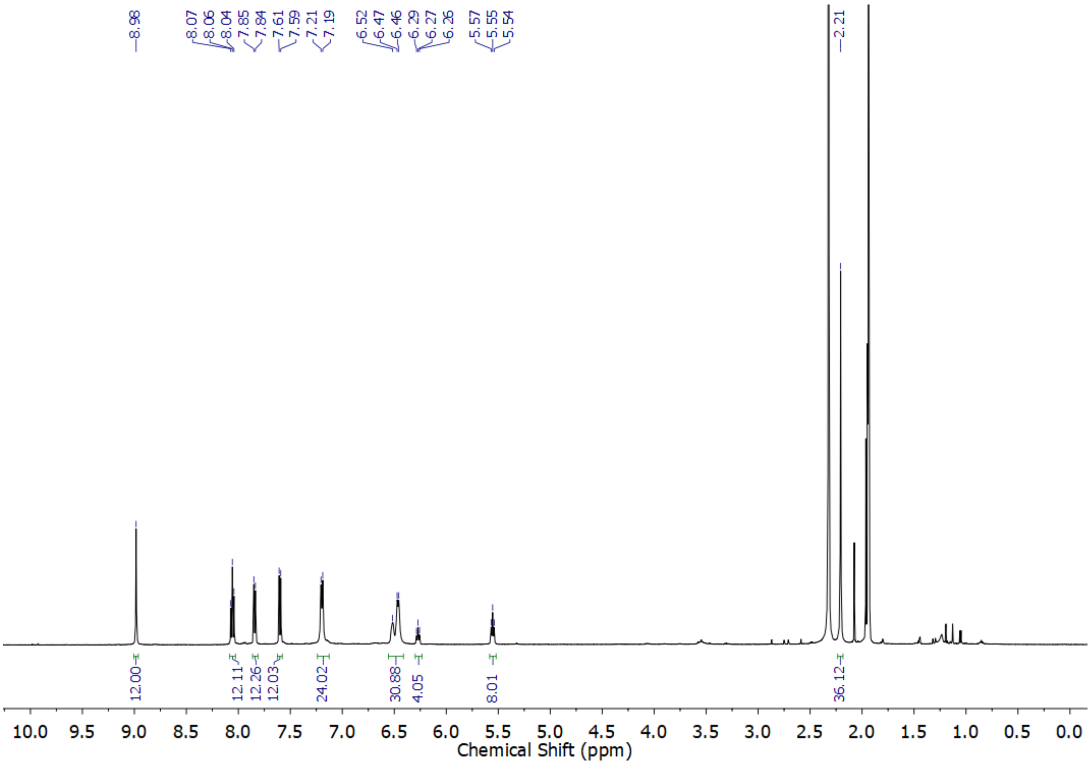

Figure S45: ^{1}H NMR spectrum of **G2**⊂*T*-**1** (500 MHz, CD_3CN, 298 K). Measured after addition of 1.0 equiv. of **G2**.

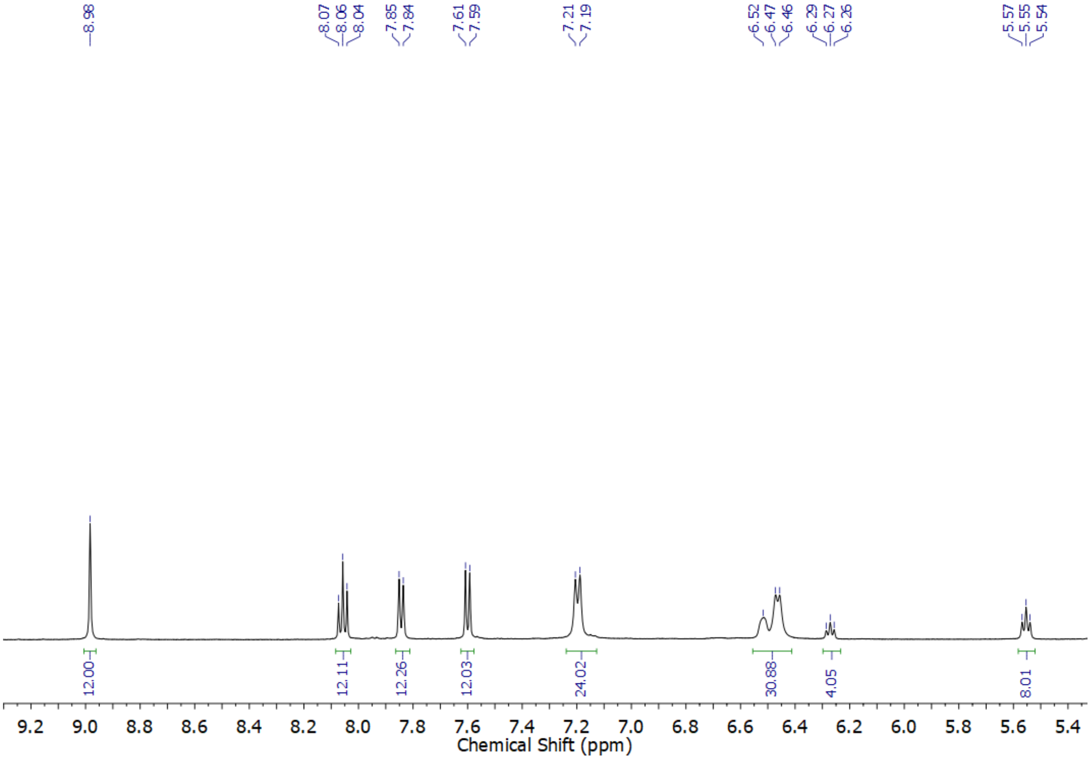

Figure S46: The aromatic region of the ^{1}H NMR spectrum of **G2**⊂*T*-**1** (500 MHz, CD_3CN, 298 K).

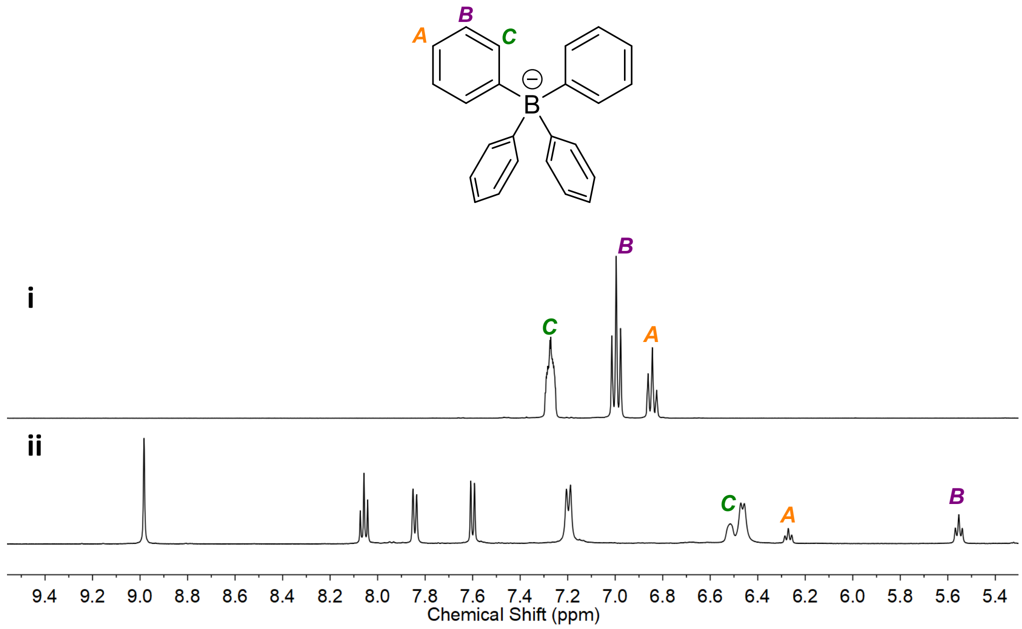

Figure S47: Stacked ^{1}H NMR spectra of: **i** free **G2** and **ii** the aromatic region of **G2**⊂*T*-**1** (500 MHz, CD_3CN, 298 K). Protons A, B and C in **G2** are labeled in both spectra to highlight the change in chemical shift of each proton environment upon encapsulation.

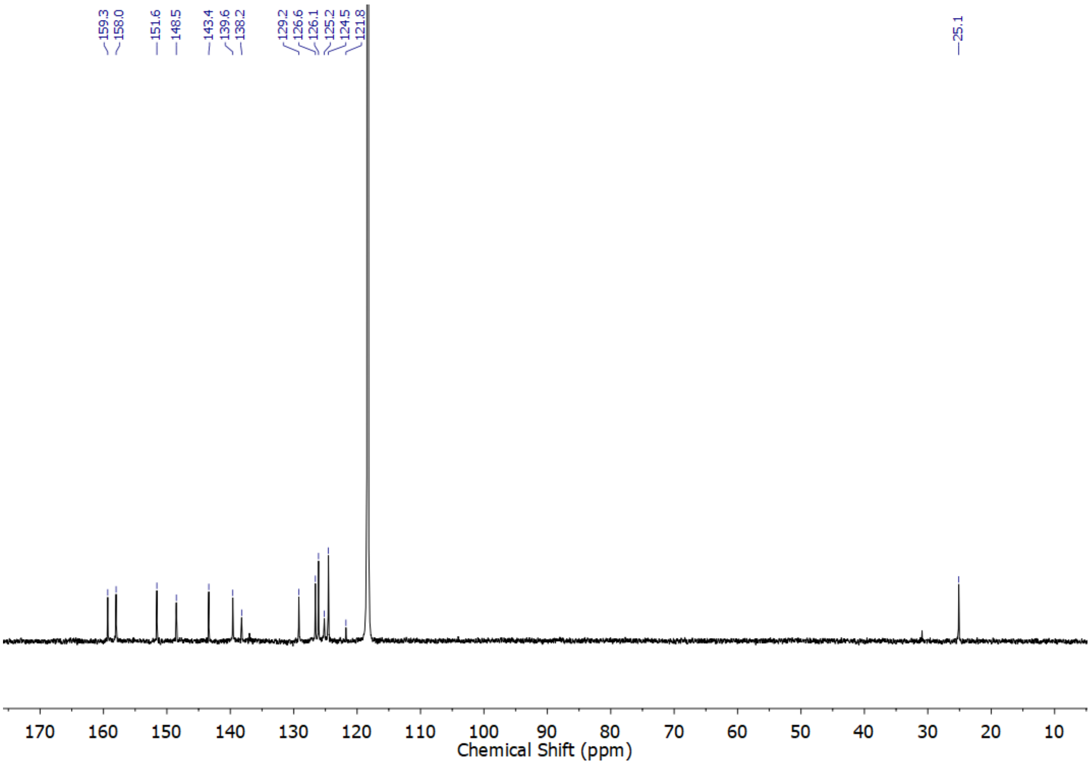

Figure S48: ^{13}C NMR spectrum of **G2**⊂*T*-**1** (126 MHz, CD_3CN, 298 K).

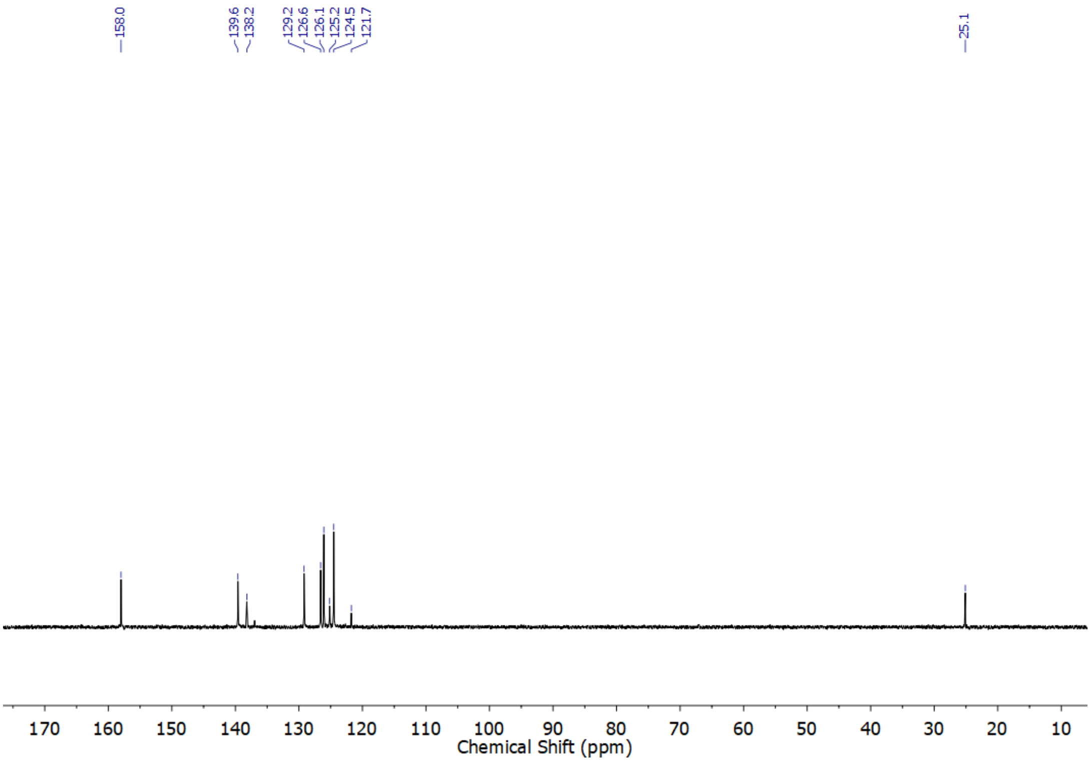

Figure S49: DEPT-135 ^{13}C NMR spectrum of **G2**⊂*T*-**1** (126 MHz, CD_3CN, 298 K).

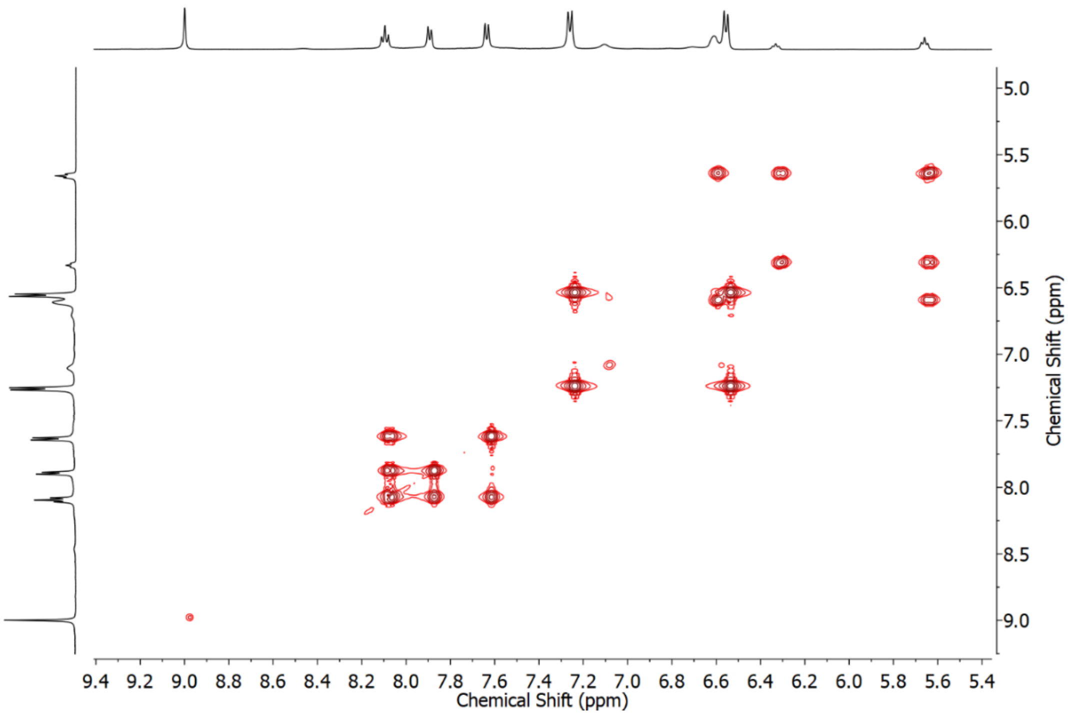

Figure S50: ^{1}H-^{1}H COSY NMR spectrum of **G2**⊂*T*-**1** (500 MHz, CD_3CN, 298 K).

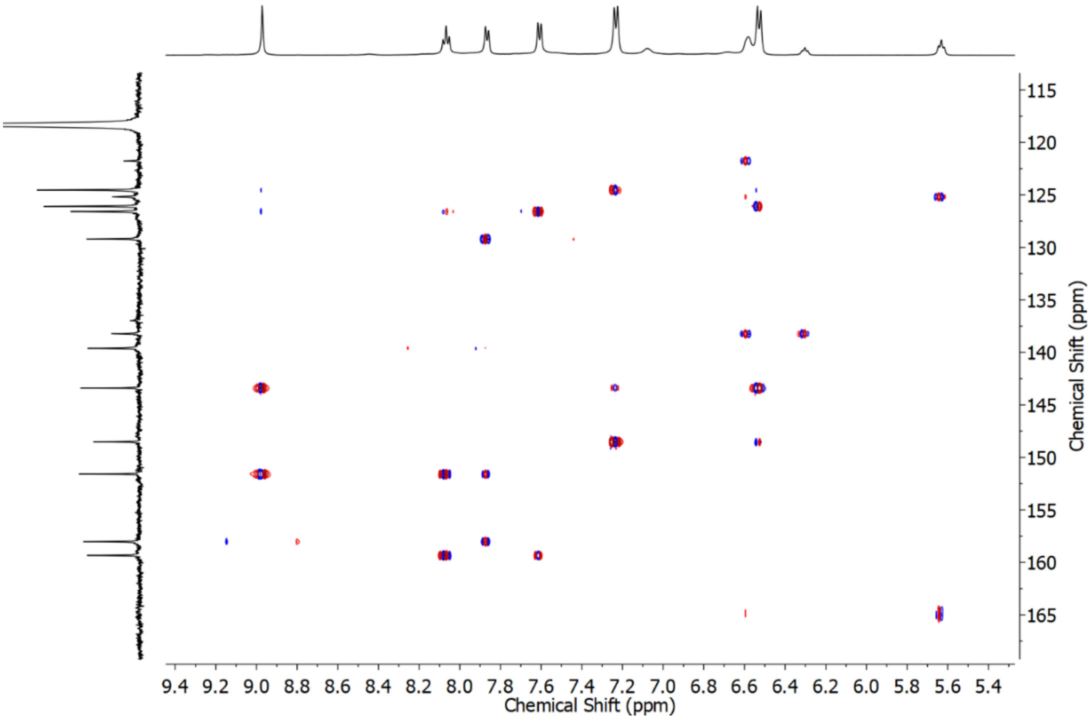

Figure S51: ^{1}H-^{13}C HMBC NMR spectrum of **G2**⊂*T*-**1** (500 MHz, CD_3CN, 298 K).

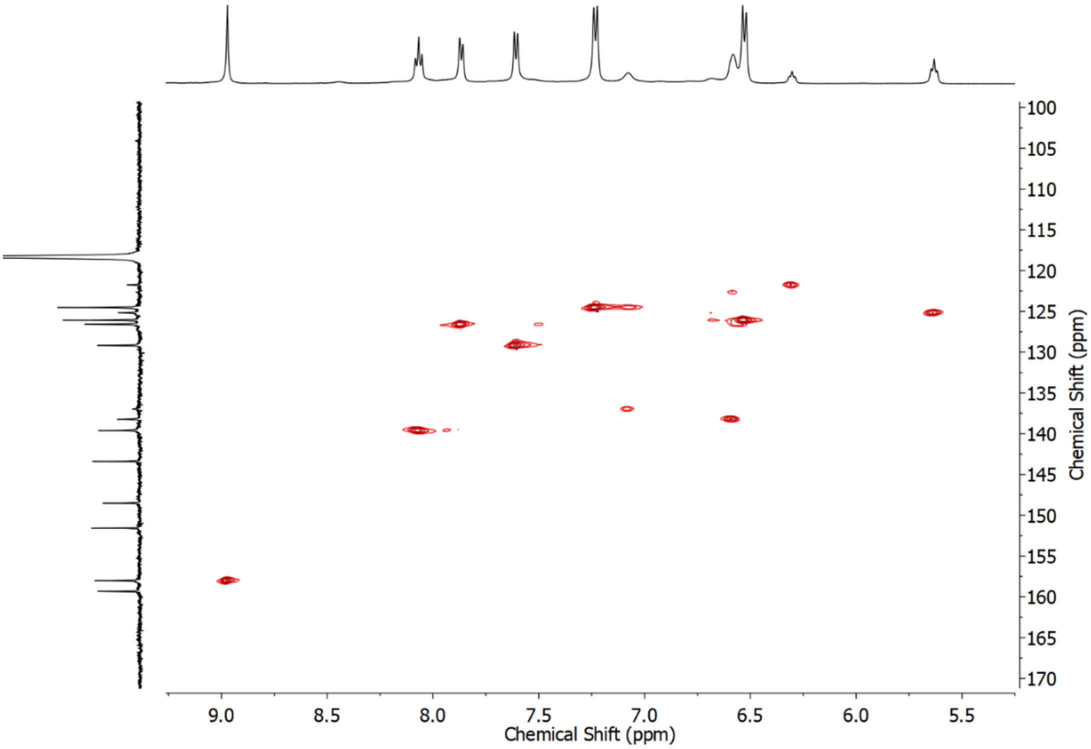

Figure S52: ^{1}H-^{13}C HSQC NMR spectrum of **G2**⊂*T*-**1** (500 MHz, CD_3CN, 298 K).

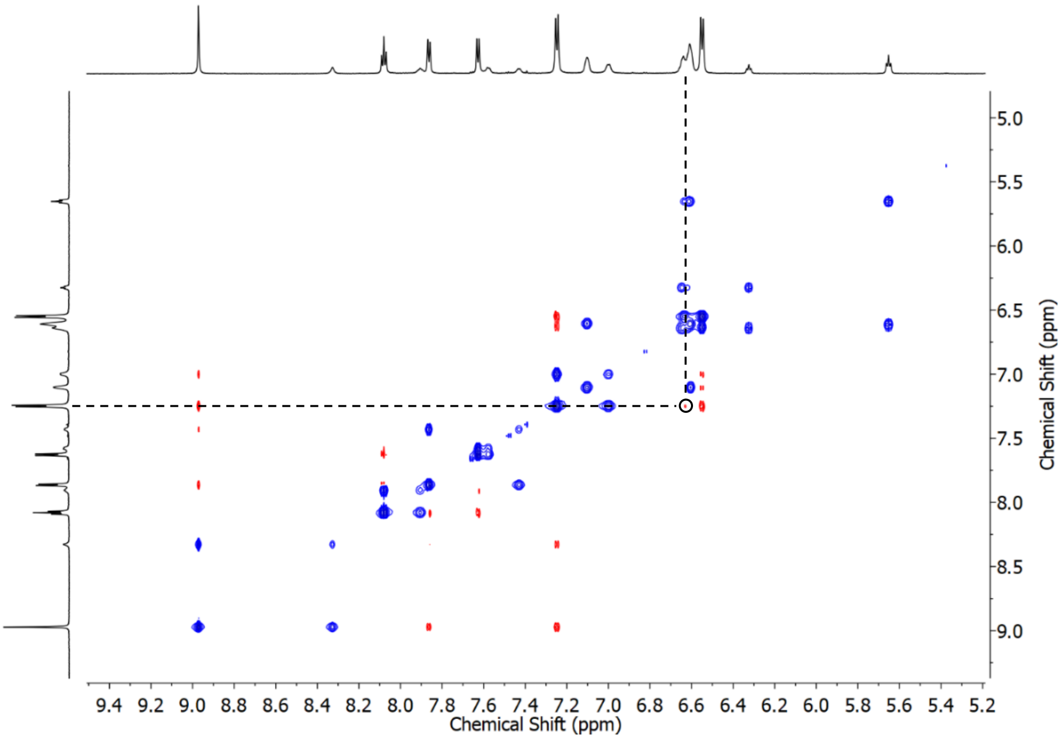

Figure S53: ^{1}H-^{1}H ROESY NMR spectrum of **G2⊂*T*-1** (700 MHz, CD_3CN, 298 K).

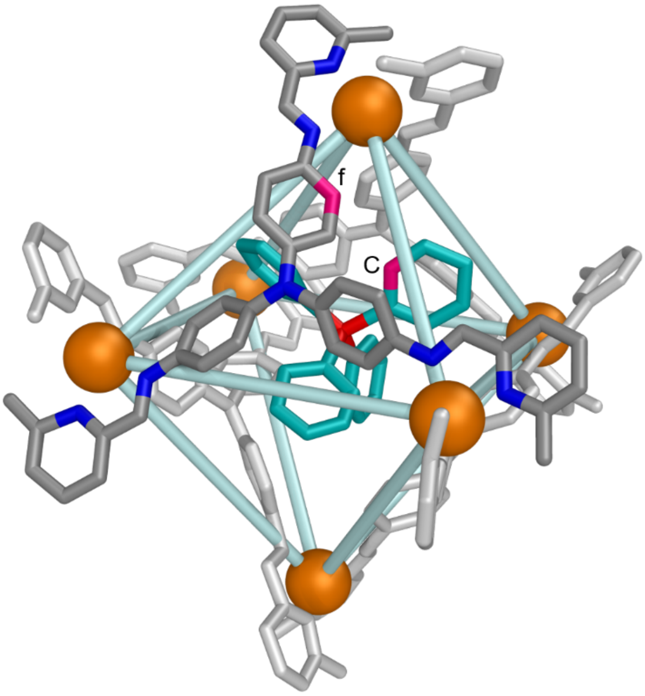

G2⊂*T*-1

Figure S54: ^{1}H-^{1}H ROESY correlations between *T*-**1** and **G2** are highlighted in pink.

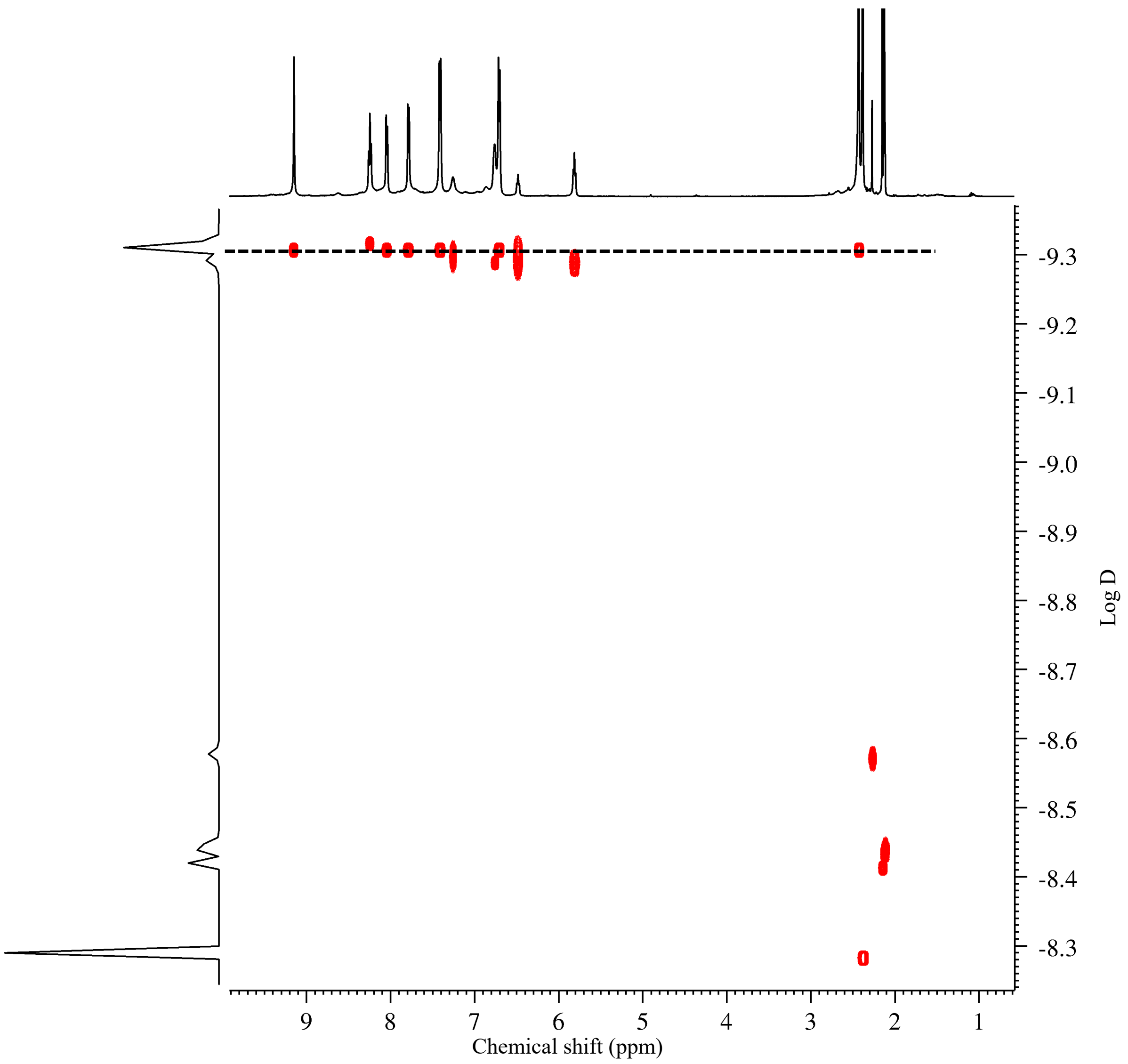

Figure S55: ^{1}H DOSY NMR spectrum of **G2**⊂*T*-**1** (400 MHz, CD_3CN, 298 K). The dashed line shows the diffusion of BPh_4^-⊂*T*-**1**, with a diffusion coefficient of 4.95×10^{-10} m^2s^{-1} corresponding to a solvodynamic radius of 13.2 Å. Residual solvent signals for acetone and H_2O are shown at approximately 2.08 and 2.13 ppm respectively.

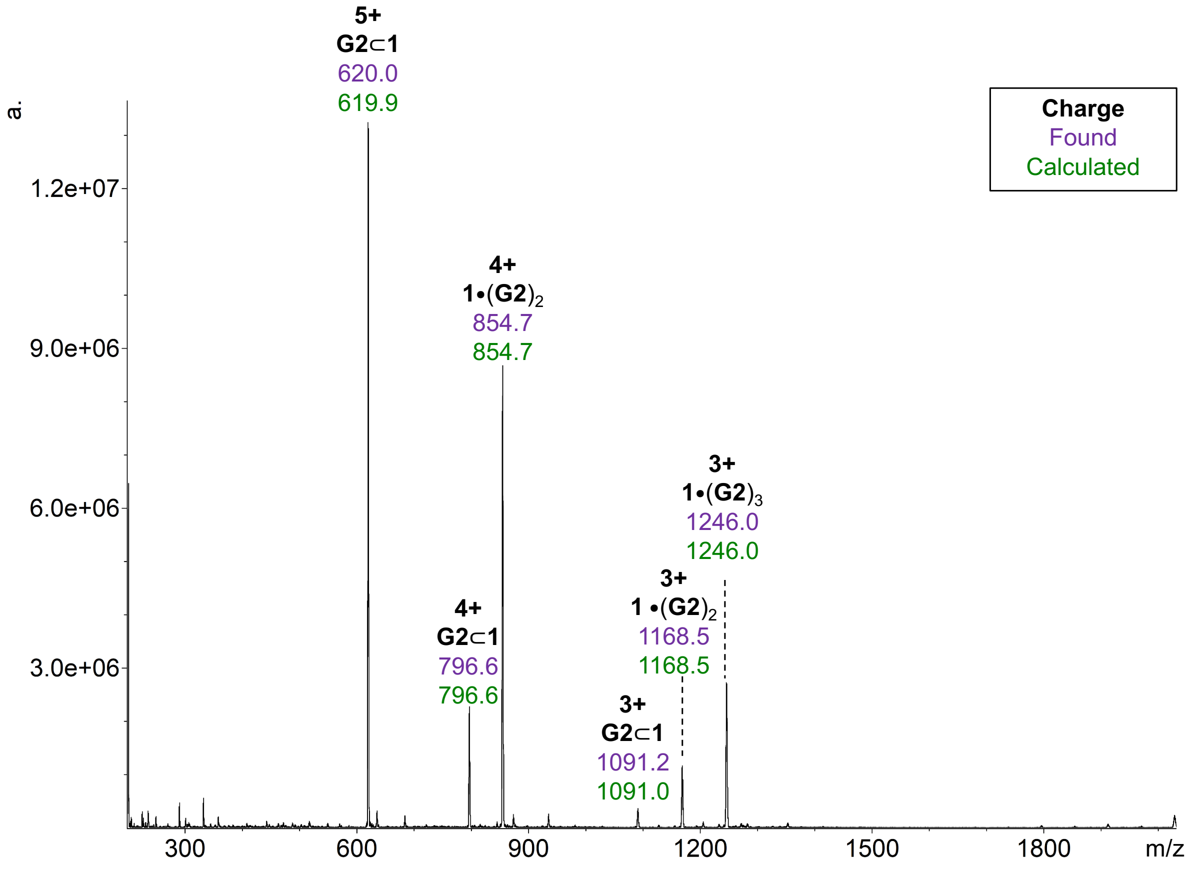

Figure S56: Low resolution ESI-mass spectrum of **G2**⊂*T*-**1**. Signals corresponding to *T*-**1**•(**G2**)$_2$ and *T*-**1**•(**G2**)$_3$ are attributed to counter-ion exchange.

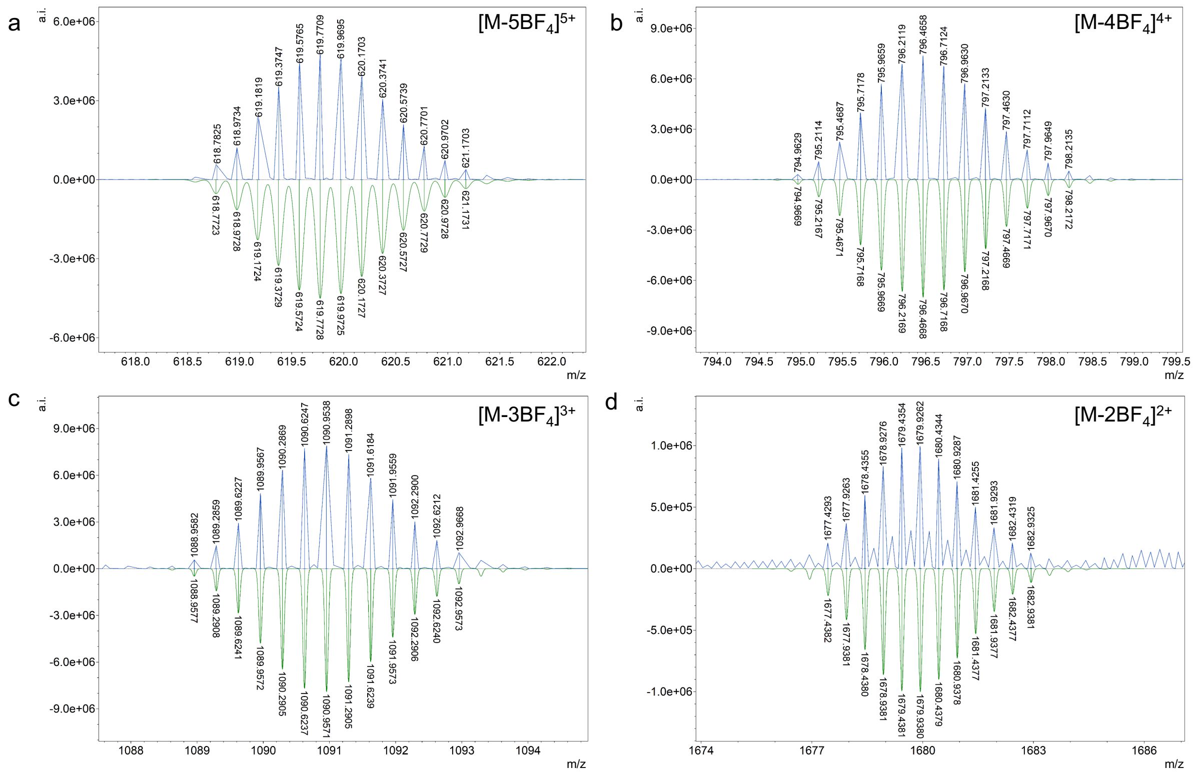

Figure S57: High resolution ESI-mass spectrum of **G2**⊂*T*-**1**. Experimental (blue) and theoretical (green) peaks for (a) $[M\text{-}5BF_4]^{5+}$; (b) $[M\text{-}4BF_4]^{4+}$; (c) $[M\text{-}3BF_4]^{3+}$; (d) $[M\text{-}2BF_4]^{2+}$.

S4. Characterization of Suit[4]ane **G3⊂*T*-1**

After determining the binding mode of **G1⊂*T*-1** and **G2⊂*T*-1**, tetrahedral guest **G3** with elongated arms was screened to form a suit[4]ane.

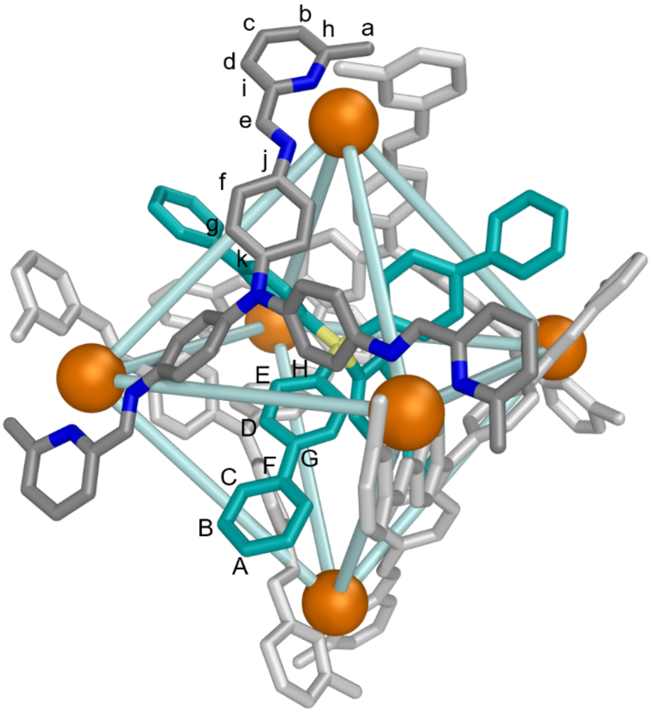

G3⊂*T*-1

δ_H (700 MHz, CD_3CN, 298 K) 8.67 (s, 12H, H_e), 8.09 (t, 12H, *J* = 7.8 Hz, H_c), 7.78 (d, 12H, *J* = 7.6 Hz, H_d), 7.62 (d, 12H, *J* = 7.8 Hz, H_b), 7.51 (t, 4H, *J* = 7.5 Hz, H_A), 7.35 (t, 8H, *J* = 7.8 Hz, H_B), 7.23 (d, 24H, *J* = 8.9 Hz, H_f), 7.00 (d, 8H, *J* = 7.2 Hz, H_C), 6.86 (d, 8H, *J* = 8.1 Hz, H_D), 6.52 (d, 24H, *J* = 8.5 Hz, H_g), 6.30 (d, 8H, *J* = 8.0 Hz, H_E), 2.22 (s, 36H, H_a).

δ_C (176 MHz, CD_3CN, 298 K) 159.4 (C_h), 158.3 (C_e), 151.3 (C_i), 147.9 (C_k), 143.6 (C_j and C_F or C_G), 141.5 (C_F or C_G), 139.7 (C_c), 138.2 (C_D), 133.1 (C_H), 130.3 (C_B), 129.5 (C_b), 129.4 (C_A), 127.5 (C_C), 127.3 (C_E), 126.8 (C_d), 125.4 (C_g), 124.8 (C_f), 25.2 (C_a).

LR-ESI-MS [POS] ([**G3⊂*T*-1**] = [$(C_{156}H_{132}N_{28}Cu_6)(C_{48}H_{36}Si)(BF_4)_6$]) *m/z* = 570.2 $[M\text{-}6BF_4]^{6+}$ (calc. 570.2), 701.6 $[M\text{-}5BF_4]^{5+}$ (calc. 701.6), 898.7 $[M\text{-}4BF_4]^{4+}$ (calc. 898.7), 1227.2 $[M\text{-}3BF_4]^{3+}$ (calc. 1227.2).

HR-ESI-MS [POS] ([**G3⊂*T*-1**] = [$(C_{156}H_{132}N_{28}Cu_6)(C_{48}H_{36}Si)(BF_4)_6$]) *m/z* = 570.1592 $[M\text{-}6BF_4]^{6+}$ (calc. 570.1592), 701.5920 $[M\text{-}5BF_4]^{5+}$ (calc. 701.5919), 898.4888 $[M\text{-}4BF_4]^{4+}$ (calc. 898.4911), 1226.9847 $[M\text{-}3BF_4]^{3+}$ (calc. 1226.9892), 1883.9739 $[M\text{-}2BF_4]^{2+}$ (calc. 1883.9857).

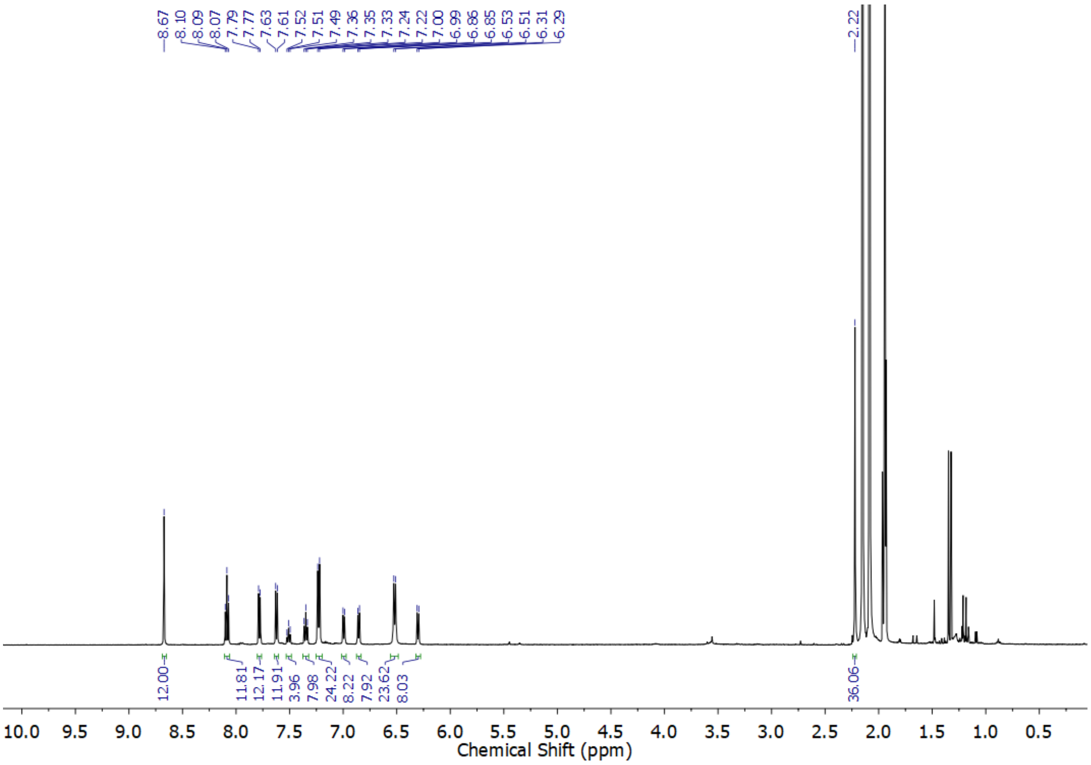

Figure S58: ^{1}H NMR spectrum of **G3**⊂*T*-**1** (700 MHz, CD_3CN, 298 K).

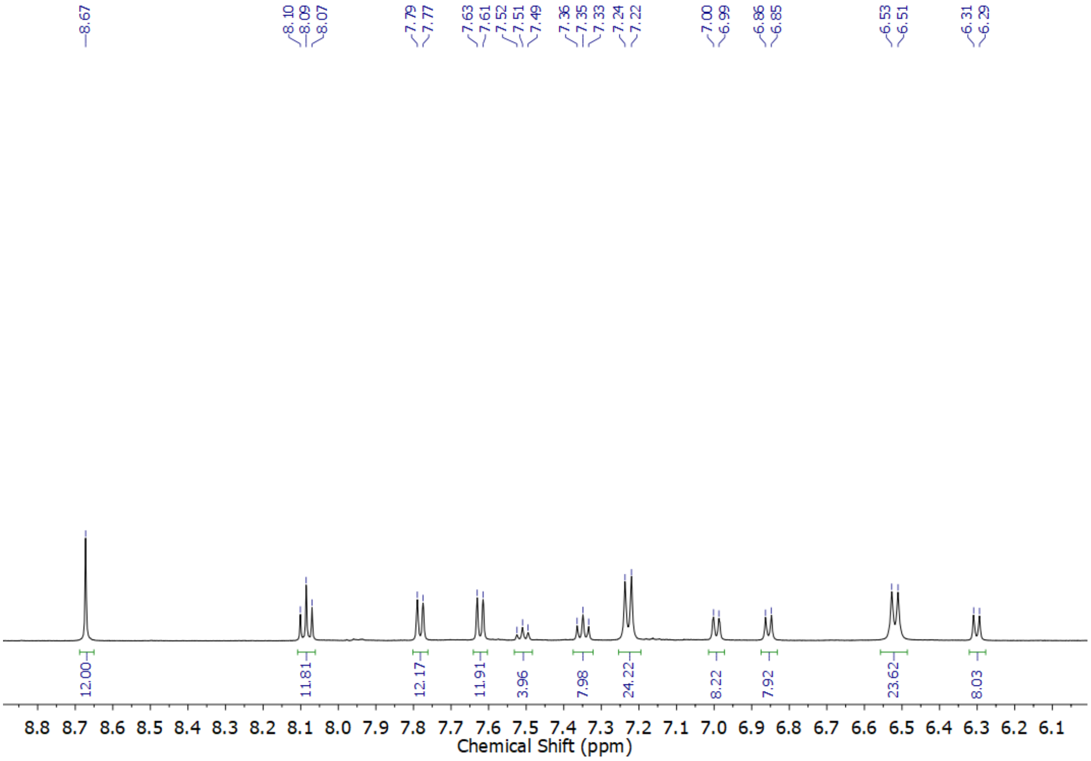

Figure S59: The aromatic region of the ^{1}H NMR spectrum of **G3**⊂*T*-**1** (700 MHz, CD_3CN, 298 K).

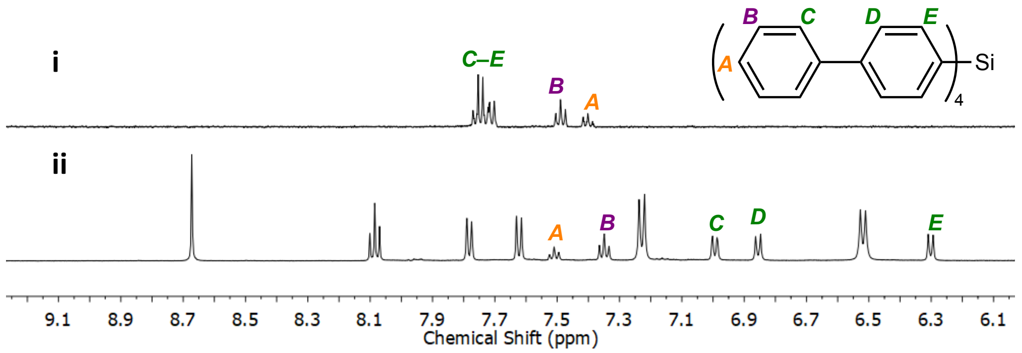

Figure S60: Stacked ^{1}H NMR spectra of: **i** free **G3** and **ii** the aromatic region of **G3**⊂*T*-**1** (500 MHz, CD_3CN, 298 K). Protons A, B, C, D and E in **G3** are labeled in both spectra to show the change in chemical shift of each proton environment upon encapsulation.

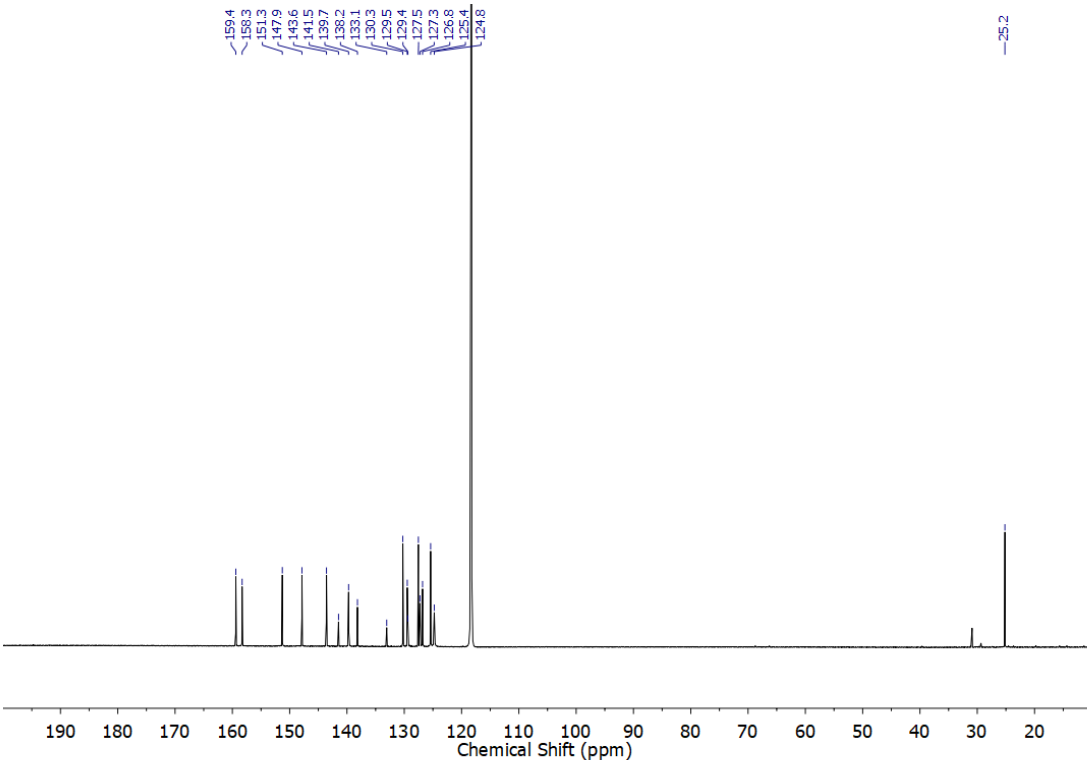

Figure S61: ^{13}C NMR spectrum of **G3**⊂*T*-**1** (176 MHz, CD_3CN, 298 K).

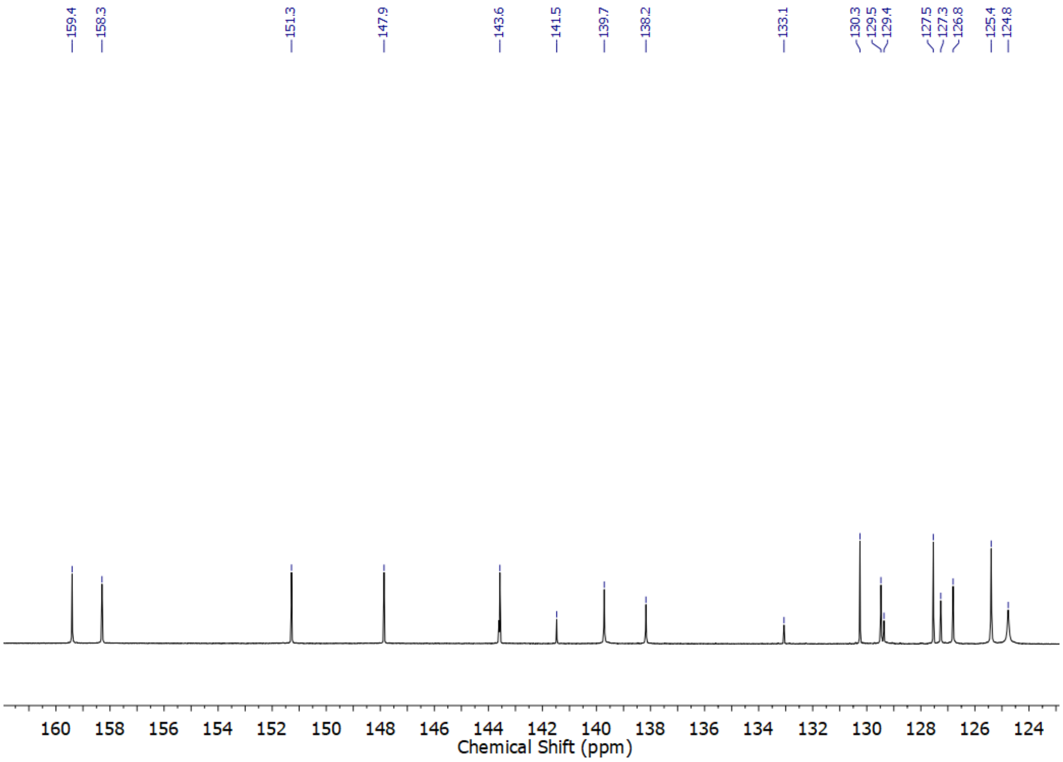

Figure S62: ^{13}C NMR spectrum of the aromatic region of **G3**⊂*T*-**1** (176 MHz, CD_3CN, 298 K).

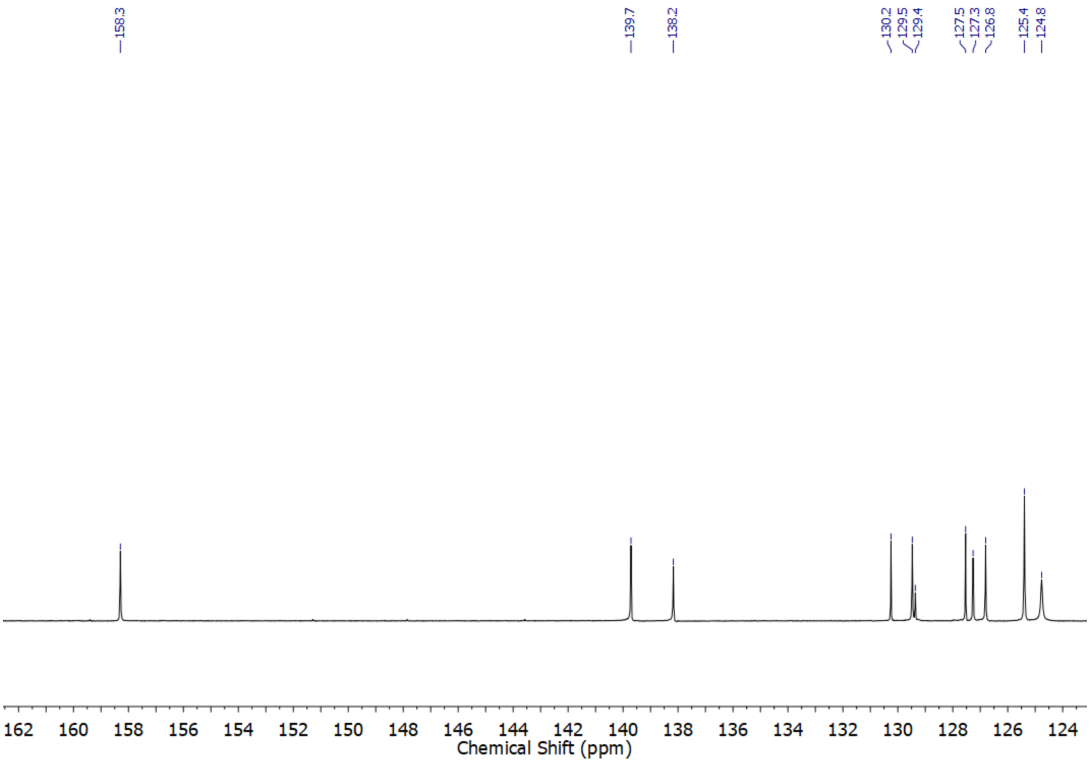

Figure S63: DEPT-135 spectrum of the aromatic region of **G3**⊂*T*-**1** (176 MHz, CD_3CN, 298 K).

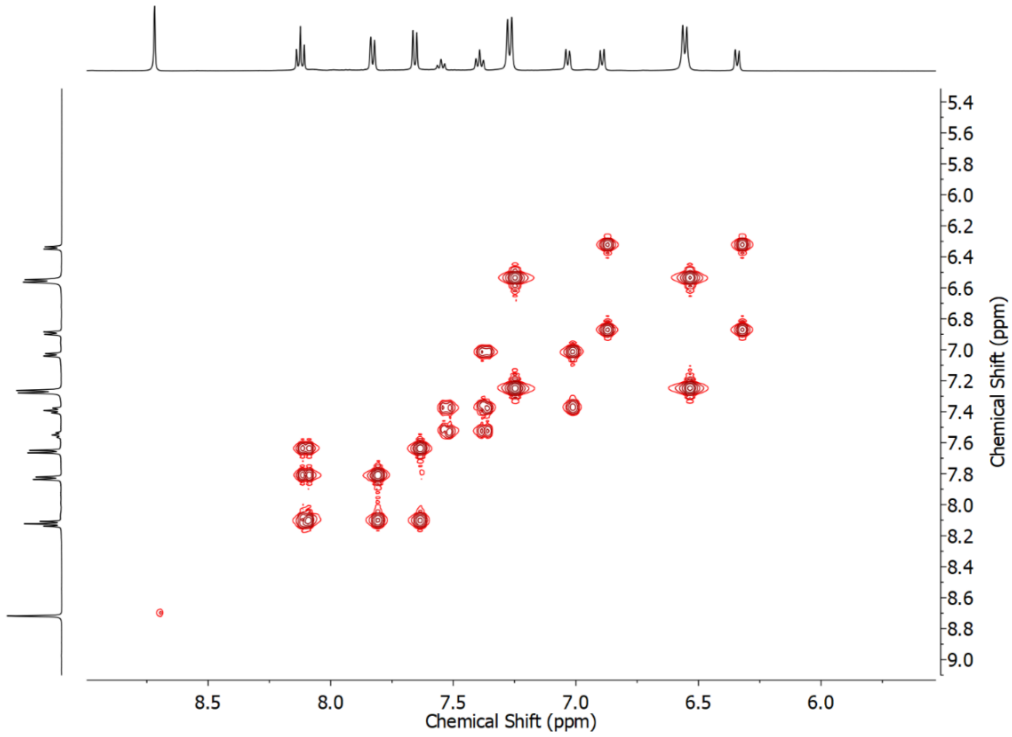

Figure S64: The aromatic region of the ^{1}H-^{1}H DQF-COSY NMR spectrum of **G3**⊂*T*-**1** (500 MHz, CD_3CN, 298 K).

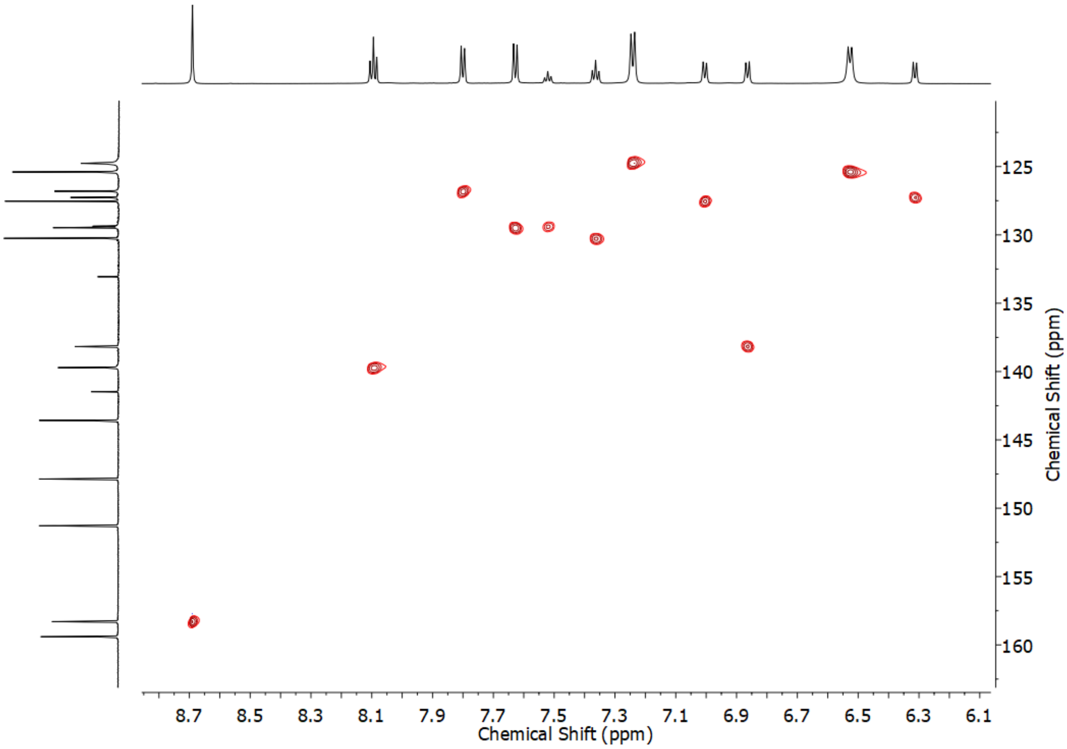

Figure S65: The aromatic region of the ^{1}H-^{13}C HSQC spectrum of **G3**⊂*T*-**1** (700 MHz, CD_3CN, 298 K).

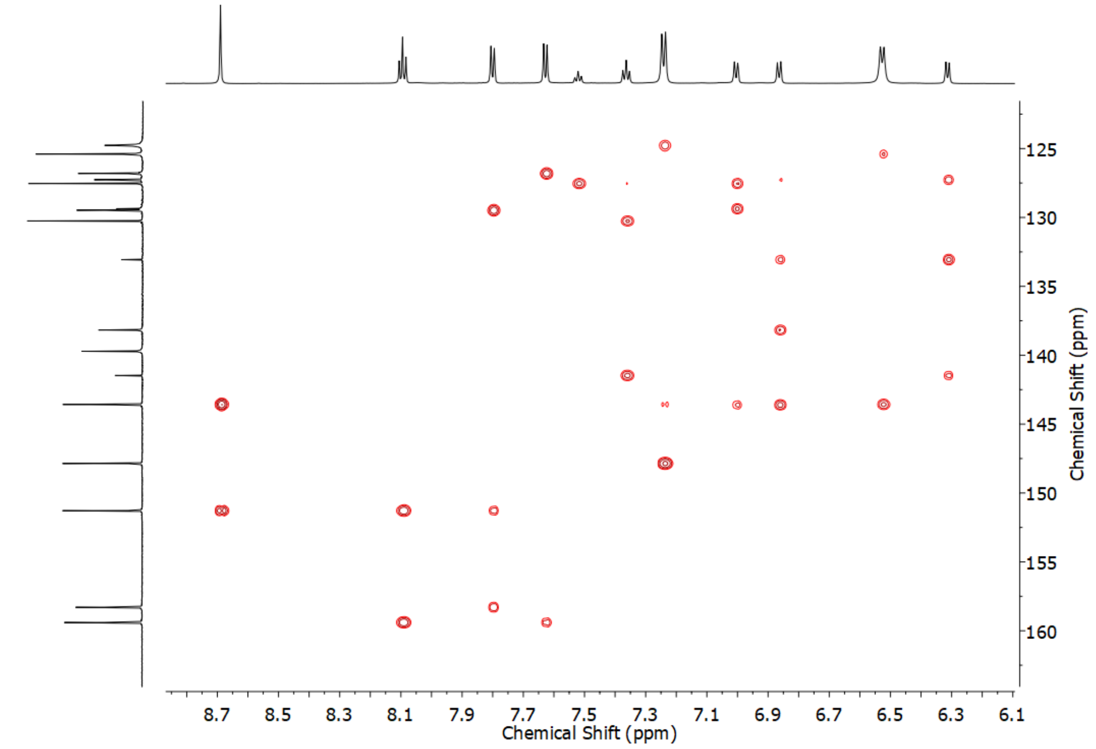

Figure S66: The aromatic region of the ^{1}H-^{13}C HMBC spectrum of **G3**⊂*T*-**1** (700 MHz, CD_3CN, 298 K).

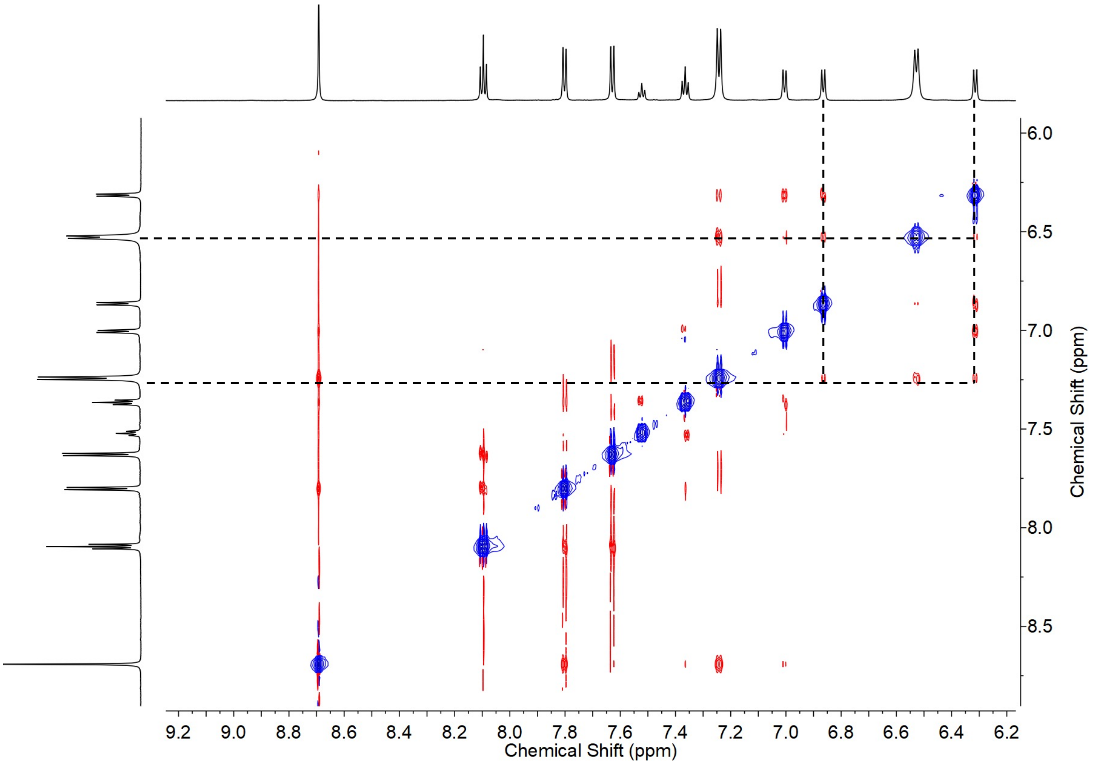

Figure S67: ^{1}H-^{1}H ROESY NMR spectrum of **G3**⊂*T*-**1** (700 MHz, CD_3CN, 298 K). ^{1}H-^{1}H through-space correlations between *T*-**1** and **G3** are shown by dashed lines.

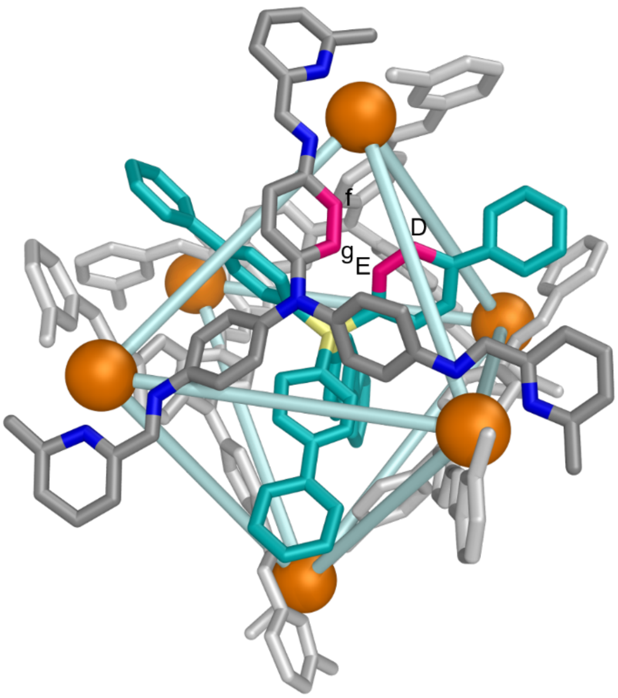

G3⊂*T*-**1**

Figure S68: [1]H-^{1}H ROESY correlations between *T*-**1** and **G3** are highlighted in pink.

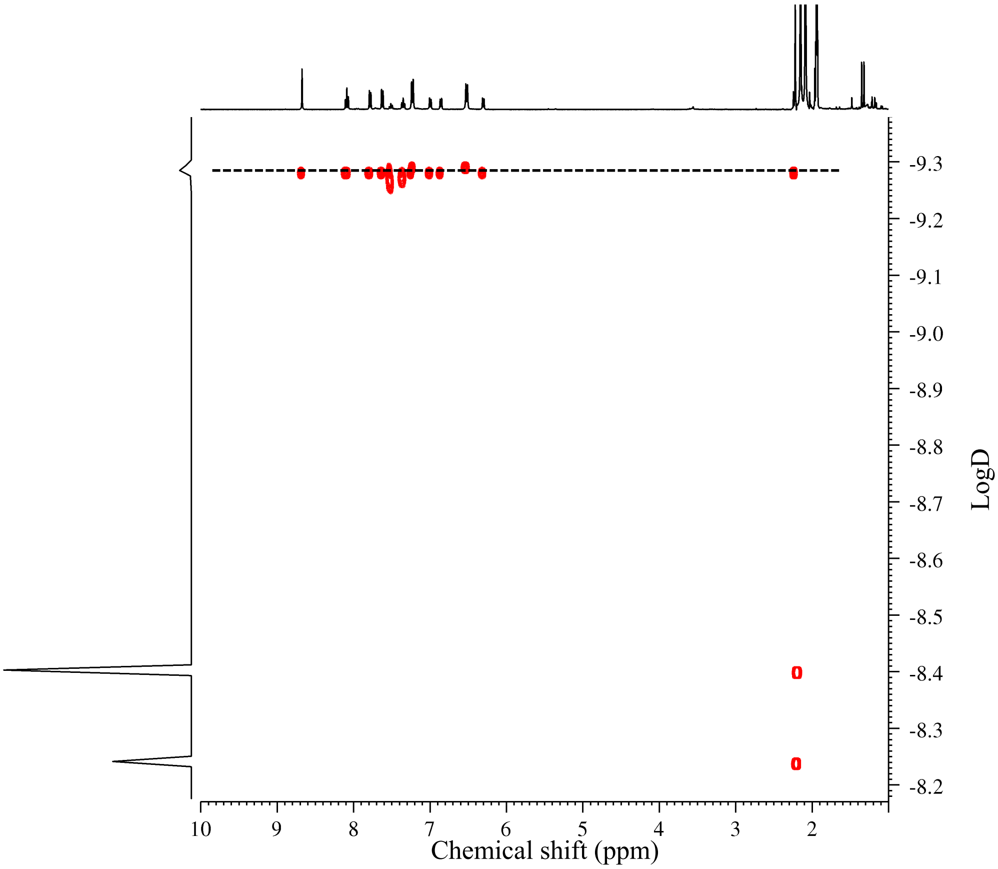

Figure S69: ¹H DOSY NMR spectrum of **G3⊂*T*-1** (400 MHz, CD_3CN, 298 K). The diffusion coefficient was measured to be 5.21 × 10^{-10} m^2s^{-1}, corresponding to a solvodynamic radius of 12.3 Å. Residual water and acetone signals are shown at approximately 2.13 and 2.18 ppm respectively.

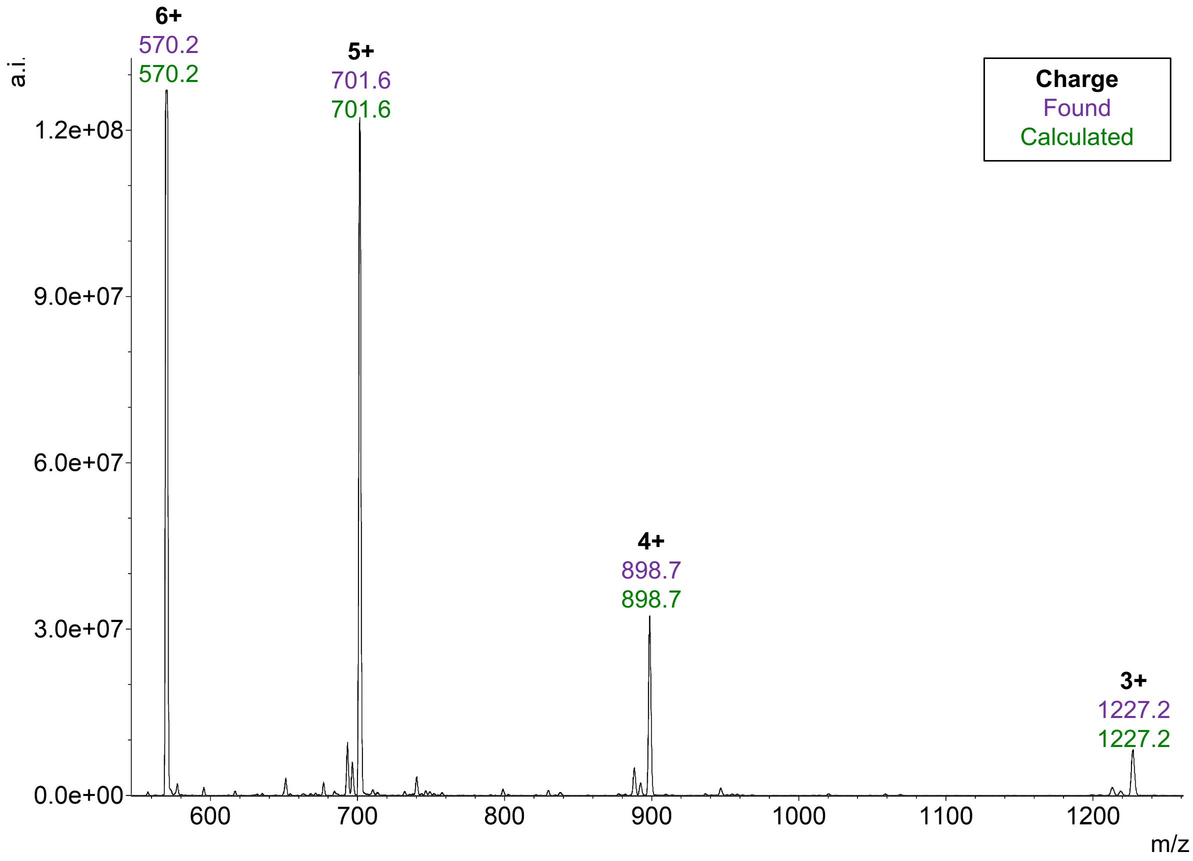

Figure S70: Low resolution ESI-mass spectrum of **G3**⊂*T*-**1**.

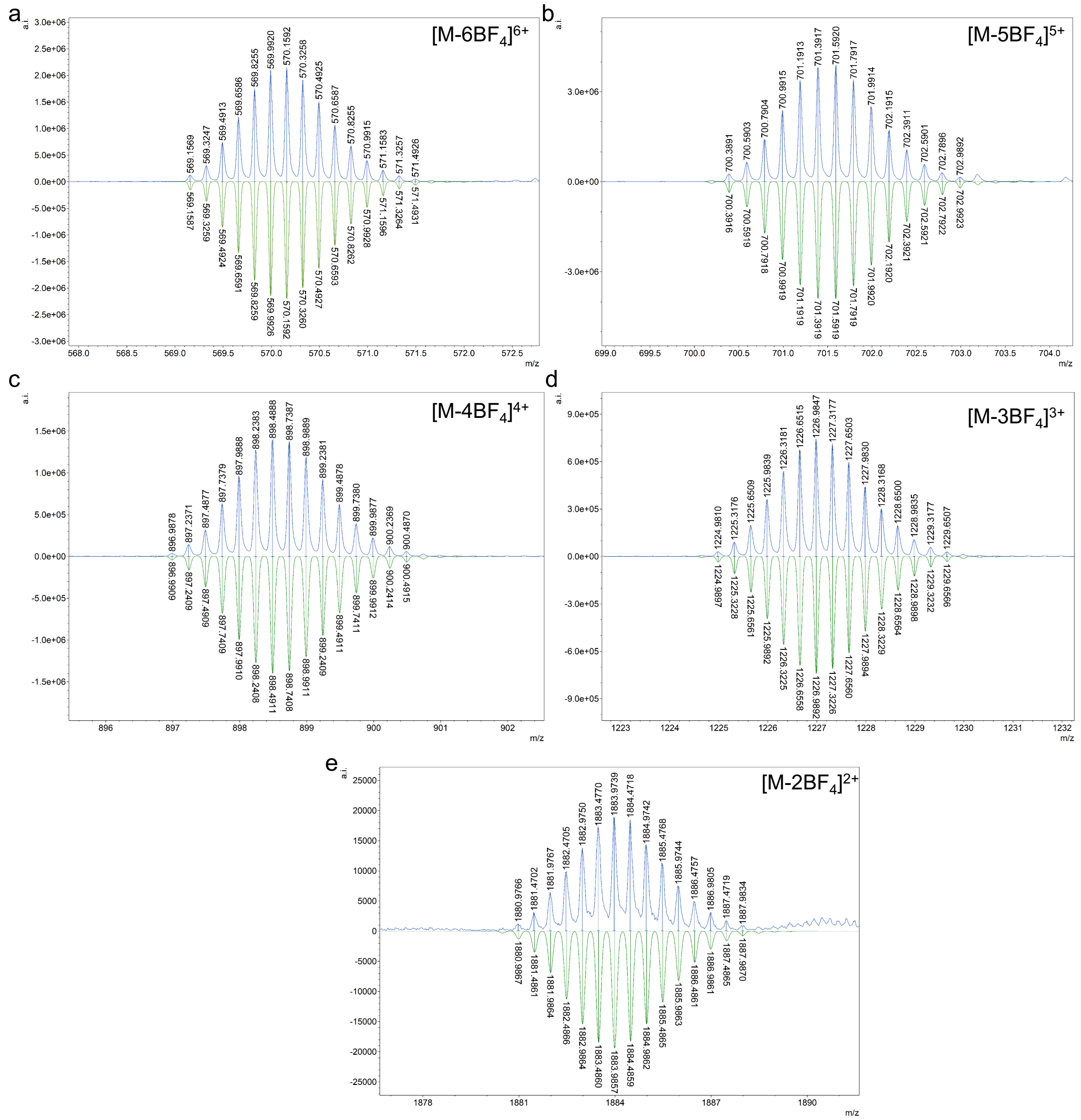

Figure S71: High resolution ESI-mass spectrum of **G3**⊂*T*-**1**. Experimental (blue) and theoretical (green) peaks for (a) $[M\text{-}6BF_4]^{6+}$; (b) $[M\text{-}5BF_4]^{5+}$; (c) $[M\text{-}4BF_4]^{4+}$; (d) $[M\text{-}3BF_4]^{3+}$; (e) $[M\text{-}2BF_4]^{2+}$.

S5 X-ray crystallography

Data were collected at Beamline I19 of the Diamond Light Source employing silicon double crystal monochromated synchrotron radiation (0.6889 Å) with ω and ψ scans at 100(2) K.[1] Data integration and reduction were undertaken with Xia2.[2–4] Subsequent computations were carried out using the WinGX-32 graphical user interface.[5] Multi-scan empirical absorption corrections were applied to the data using the AIMLESS[6] tool in the CCP4 suite.[7] The structures were solved by direct methods using SHELXT[8] then refined and extended with SHELXL.[9] In general, non-hydrogen atoms with occupancies greater than 0.5 were refined anisotropically. Carbon-bound hydrogen atoms were included in idealized positions and refined using a riding model. Disorder was modeled using standard crystallographic methods including constraints, restraints and rigid bodies where necessary. Crystallographic data along with specific details pertaining to the refinement follow. Crystallographic data have been deposited with the CCDC (2302005 and 2302006).

S5.1 X-ray crystallography of S_4-**1**

Specific refinement details:

Single crystals of S_4-**1**·$6BF_4$·$3Et_2O$·7.25MeCN [+ solvent] were grown by the slow diffusion of Et_2O into an MeCN solution of **1**·$6BF_4$. The crystals employed rapidly lost solvent after removal from the mother liquor and rapid handling prior to flash cooling in the cryostream was required to collect data. Few reflections at greater than 0.65 Å resolution were observed and the data were trimmed accordingly. The asymmetric unit was found to contain half of a Cu_6L_4 assembly. Part of one ligand was modeled as disordered over two positions and the relative occupancy allowed to freely refine. Both parts were modeled isotropically. Thermal parameter restraints (SIMU) were applied to all disordered atoms.

Two of the tetrafluoroborate anions were modeled as disordered over two positions. Bond length and thermal parameters were applied to facilitate a reasonable refinement of the disordered anions.

There was also significant disorder in some of the Et_2O and MeCN molecules. A few electron density peaks > 1.0 that couldn't be modeled were observed, likely as a result of further disorder of the solvent. Consequently, the SQUEEZE[10] function of PLATON[11] was employed to remove the contribution of the electron density associated with the highly disordered solvent and counterions, which gave two potential solvent-accessible voids with a total volume of 314 Å^3 per unit cell, containing a total of approximately 72 electrons. Diffuse solvent molecules could not be assigned to acetonitrile or diethyl ether and were therefore not included in the formula. Consequently, the molecular weight and density given above are underestimated.

CheckCIF gives one B level alert, resulting from the isotropic modeling of one of the disordered ligands.

Table 1 Crystal data and structure refinement for *S*$_4$-1.

Identification code	cut065
Empirical formula	$C_{182}H_{183}B_6Cu_6F_{24}N_{35}O_3$
Formula weight	3810.72
Temperature/K	100
Crystal system	triclinic
Space group	P-1
a/Å	17.0931(2)
b/Å	17.55590(10)
c/Å	35.6777(3)
α/°	100.4940(10)
β/°	93.6770(10)
γ/°	107.4450(10)
Volume/Å^3	9962.28(17)
Z	2
ρ_{calc}g/cm^3	1.270
μ/mm^{-1}	0.655
F(000)	3928.0
Crystal size/mm^3	0.02 × 0.01 × 0.01
Radiation	Synchrotron (λ = 0.6889)
2Θ range for data collection/°	2.268 to 64
Index ranges	-26 ≤ h ≤ 26, -27 ≤ k ≤ 27, -54 ≤ l ≤ 54
Reflections collected	195377
Independent reflections	73878 [R_{int} = 0.0419, R_{sigma} = 0.0488]
Data/restraints/parameters	73878/1037/2451
Goodness-of-fit on F^2	1.037
Final R indexes [I>=2σ (I)]	R_1 = 0.0757, wR_2 = 0.2405
Final R indexes [all data]	R_1 = 0.1046, wR_2 = 0.2553
Largest diff. peak/hole / e Å^{-3}	1.52/-1.88

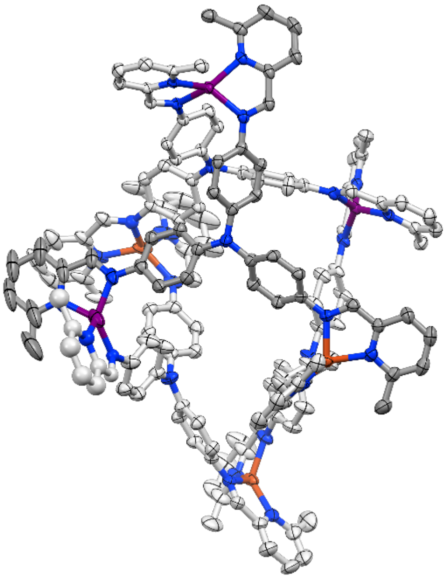

Figure S72: ORTEP plot of the cationic part of the crystal structure of S_4-**1**, showing thermal ellipsoids at 50%. Disorder is omitted for clarity.

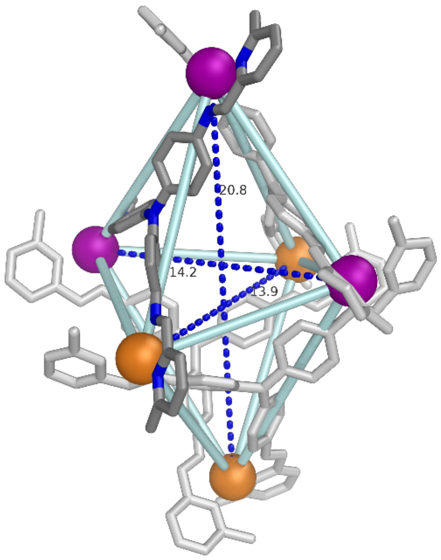

Figure S73: Crystal structure of the cationic part of S_4-**1** with the Δ···Λ, Δ···Δ and Λ···Λ distances labeled as 20.8 Å, 14.2 Å and 13.9 Å respectively. Disorder and hydrogen atoms are omitted for clarity.

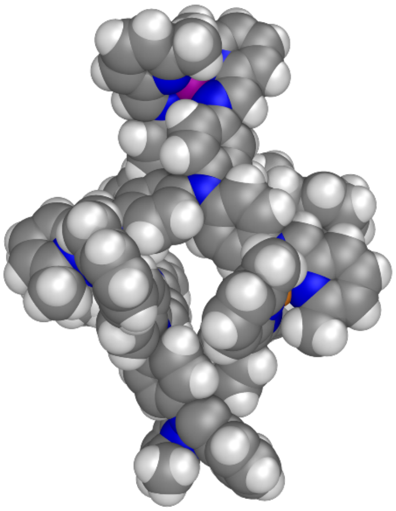

Figure S74: Space-filling representation of the X-ray structure of the cationic part of S_4-**1**.

S5.2 X-ray crystallography of **G3**⊂*T*-**1**

Specific refinement details:

Single crystals of [**G3**⊂*T*-**1**]·6BF$_4$·4.25MeCN [+ solvent] were grown by the slow diffusion of Et$_2$O into an MeCN solution of [**G3**⊂*T*-**1**]·6BF$_4$. The crystals rapidly lost solvent after removal from the mother liquor and rapid handling prior to flash cooling in the cryostream was required to collect data. Despite these measures and the use of synchrotron radiation, few reflections at greater than 1.0 Å resolution were observed and the data were trimmed accordingly.

Due to the limited resolution, bond lengths and angles within chemically identical ligands were restrained to be similar to each other and thermal parameter restraints (SIMU, RIGU) were applied to all atoms except for copper. One of the cage vertices was highly disordered, with the copper ion and coordinating ligands being modeled over two positions.

G3 also showed evidence of disorder. Four phenyl rings in **G3** were modeled as disordered over two positions and bond lengths and angles within chemically identical groups were restrained to be similar to each other.

The tetrafluoroborate anions also showed evidence of disorder. Eight tetrafluoroborate anions were modeled as disordered over two locations. Substantial bond length and thermal parameter restraints were applied to allow a reasonable refinement of the disordered tetrafluoroborate anions and solvent. Most low occupancy disordered groups were modeled with isotropic thermal parameters. One tetrafluoroborate anion could not be located. Consequently, the SQUEEZE[10] function of PLATON[11] was employed to remove the contribution of the electron density associated with the highly disordered solvent and

tetrafluoroborate anion, which gave a potential solvent-accessible void with a total volume of 4688 Å^3 per unit cell, containing a total of approximately 1300 electrons. Diffuse solvent molecules could not be assigned to acetonitrile or diethyl ether and were therefore not included in the formula. Consequently, the molecular weight and density given above are underestimated.

Due to the low resolution of the data some of the hydrogen atoms on the MeCN molecules could not be located in the electron density map and so were omitted from the refinement.

CheckCIF gives one A and one B alert. The A alert is due to the weak diffraction resulting in few reflections observed at greater than 1.0 Å and so the data was trimmed accordingly. The B alert relates to the low bond precision of the C-C bonds due to the limited resolution of the data and the thermal motion and disorder in some areas of the structure.

Table 2 Crystal data and structure refinement for G3⊂*T*-1.

Identification code	cut10
Empirical formula	$C_{212.5}H_{180.75}B_6Cu_6F_{24}N_{32.25}Si$
Formula weight	4116.32
Temperature/K	100(2)
Crystal system	monoclinic
Space group	$P2_1/n$
a/Å	28.4545(3)
b/Å	31.8770(3)
c/Å	48.9539(6)
α/°	90
β/°	95.6430(10)
γ/°	90
Volume/Å^3	44188.2(8)
Z	8
ρ_{calc}g/cm^3	1.237
μ/mm^{-1}	0.599
F(000)	16924.0
Crystal size/mm^3	0.1 × 0.08 × 0.08
Radiation	Synchrotron (λ = 0.6889)
2Θ range for data collection/°	1.48 to 40.296
Index ranges	-28 ≤ h ≤ 28, -31 ≤ k ≤ 31, -48 ≤ l ≤ 47
Reflections collected	225311
Independent reflections	46141 [R_{int} = 0.1204, R_{sigma} = 0.1161]
Data/restraints/parameters	46141/4808/5121
Goodness-of-fit on F^2	0.940
Final R indexes [I>=2σ (I)]	R_1 = 0.1081, wR_2 = 0.2910
Final R indexes [all data]	R_1 = 0.1655, wR_2 = 0.3180
Largest diff. peak/hole / e Å^{-3}	1.10/-0.63

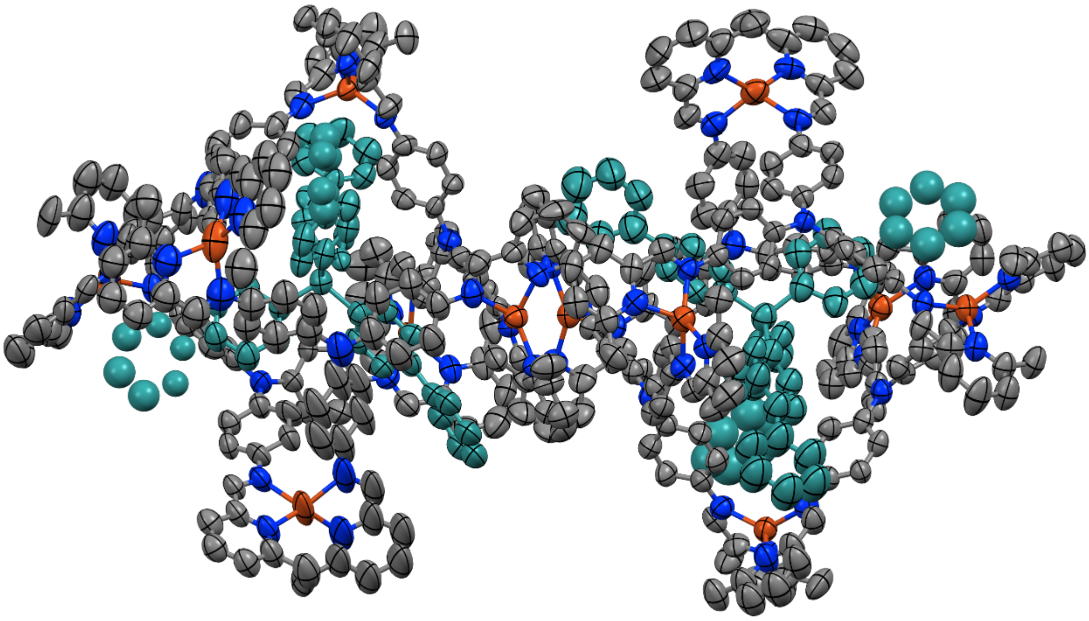

Figure S75: ORTEP plot of the cationic part of the crystal structure of **G3**⊂*T*-**1**. Thermal ellipsoids are set at 50%. Some of the terminal phenyl rings of **G3** are highly disordered. Only one part is shown here.

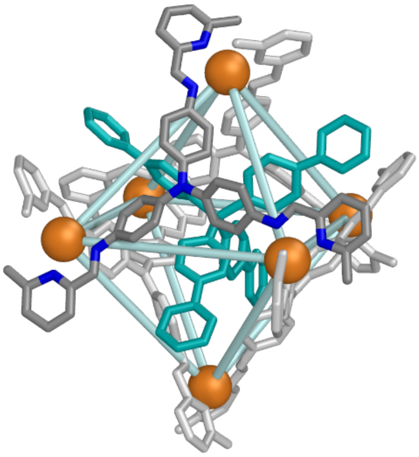

Figure S76: X-ray crystal structure of the cationic part of **G3**⊂*T*-**1**. Disorder and hydrogen atoms are omitted for clarity.

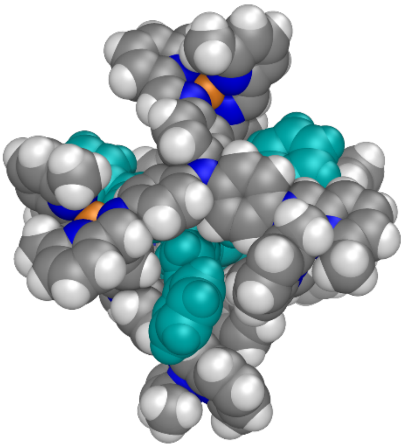

Figure S77: Space-filling representation of the cationic part of X-ray structure of **G3**⊂*T*-**1**.

S6. Binding constants of tetrahedral guests

The binding of **G1, G2** and **G3** stabilized *T*-**1** at 298 K and 253 K. Addition of an excess of **G1**, **G2** or **G3** to a solution of **1** in CD_3CN gave ^1H NMR spectra at 298 K that did not show any free guest signals. Addition of < 1.0 equiv. of guest showed two sets of cage signals, corresponding to the S_4 and T diastereomers, indicating that encapsulation was occurring in slow exchange on the NMR timescale. It can be inferred from these observations that **G1–G3** had association constants exceeding the limit for measurement by ^1H NMR (>10^5 M^{-1}).[12]

Only **G2** had a high enough solubility in MeCN for an accurate association constant to be calculated by isothermal titration calorimetry (ITC). K_1 was calculated as 5.4(2) × 10^6 M^{-1}.

6.1 Procedure

The experiment was carried out at 298 K with a solution of **1** in MeCN (120 μM) in the cell and a solution of **G2** in MeCN (1.1 mM) in the syringe. The injection volume was 2 μL. Two consecutive experiments were run where the cell was left intact, but the syringe was refilled. The data were concatenated and processed as one experiment. The data were not sigmoidal and so were fitted with using the sequential binding model in the manufacturer's software. Four *K* values were used to obtain a fit (Fig. S78). Due to this, only the value of K_1 shown in Fig. S78 can be taken from the data.

The non-sigmoidal nature of the data fits with the observations by NMR suggesting one internal binding site followed by weaker secondary interactions, hypothesized to occur on the exterior of the cage due to electrostatic interactions.

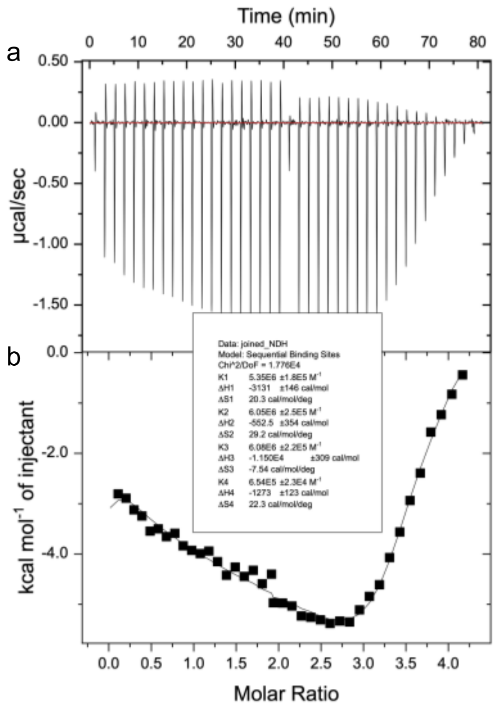

Figure S78: **a**. Raw data from the ITC titration. **b**. Binding isotherm from the integrated calorimetric titration data.

S7. Steroid binding in 1

A range of steroids (**G4–G11**) were screened for binding with **1**.

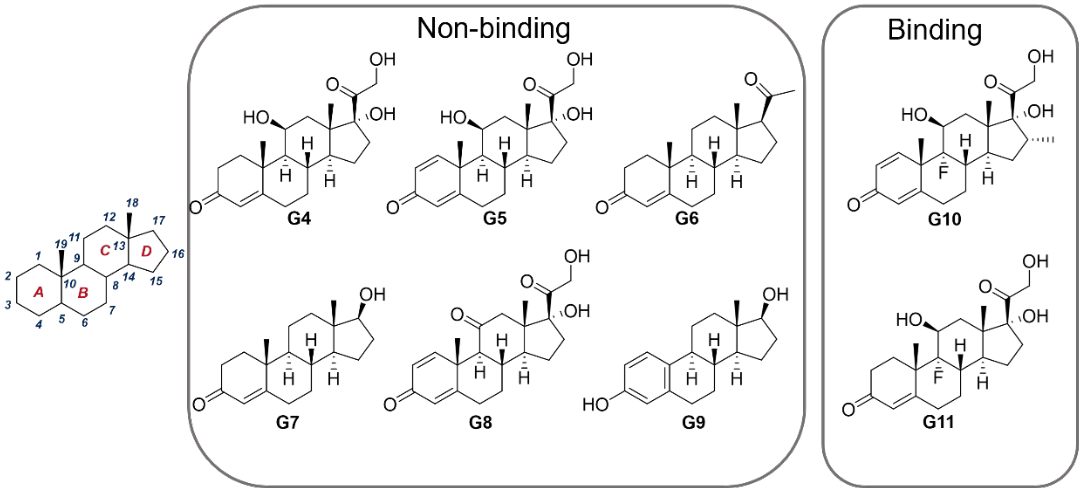

Figure S79: Steroid molecules screened for binding interactions with **1**.

S7.1 Characterization of G10⊂1

Changes in the ^{1}H NMR spectrum of **1** were only seen upon addition of **G10** at 253 K but not at 298 K. For **G10**, a single set of minor signals appeared alongside the S_4-**1** signals, assigned to the *T*-**1** diastereomer. These changes in the ^{1}H NMR spectrum of cage **1** were inferred to indicate an interaction between **1** and **G10**.

Addition of excess **G10** gave a ratio of S_4-**1** : *T*-**1** that was calculated to be 6.4 : 1.0 by deconvolution of the integrals in the ¹H NMR spectrum. This was not changed upon heating overnight.

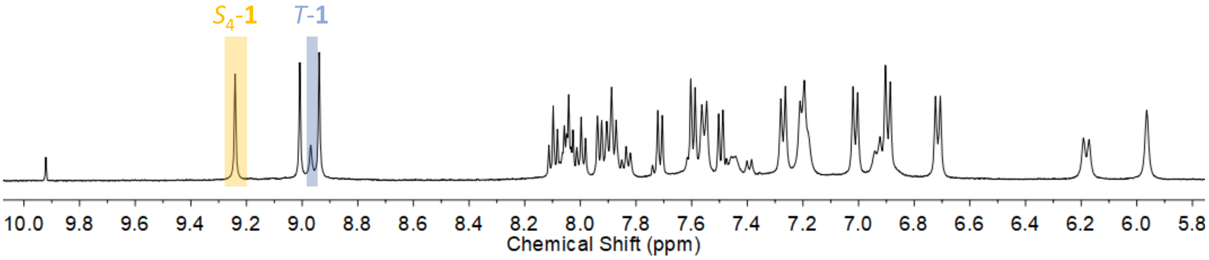

Figure S80: ¹H NMR spectrum of **G10**⊂**1** (500 MHz, CD_3CN, 253 K). The S_4-**1** and *T*-**1** signals used for deconvolution are labeled.

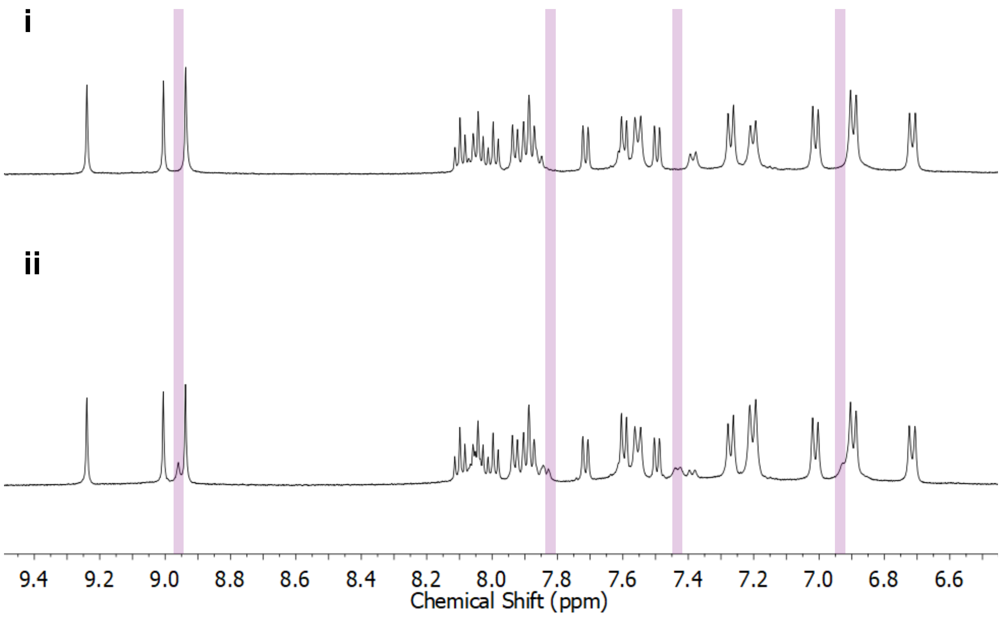

Figure S81: Stacked ¹H NMR spectra of the aromatic region of **i 1 ii 1** and **G10** (500 MHz, CD_3CN, 253 K). Some of the new signals for **1**, assigned to the *T*-**1** diastereomer, that appear upon addition of **G10** are highlighted. Due to signal overlap, not all of the signals of *T*-**1** could be identified.

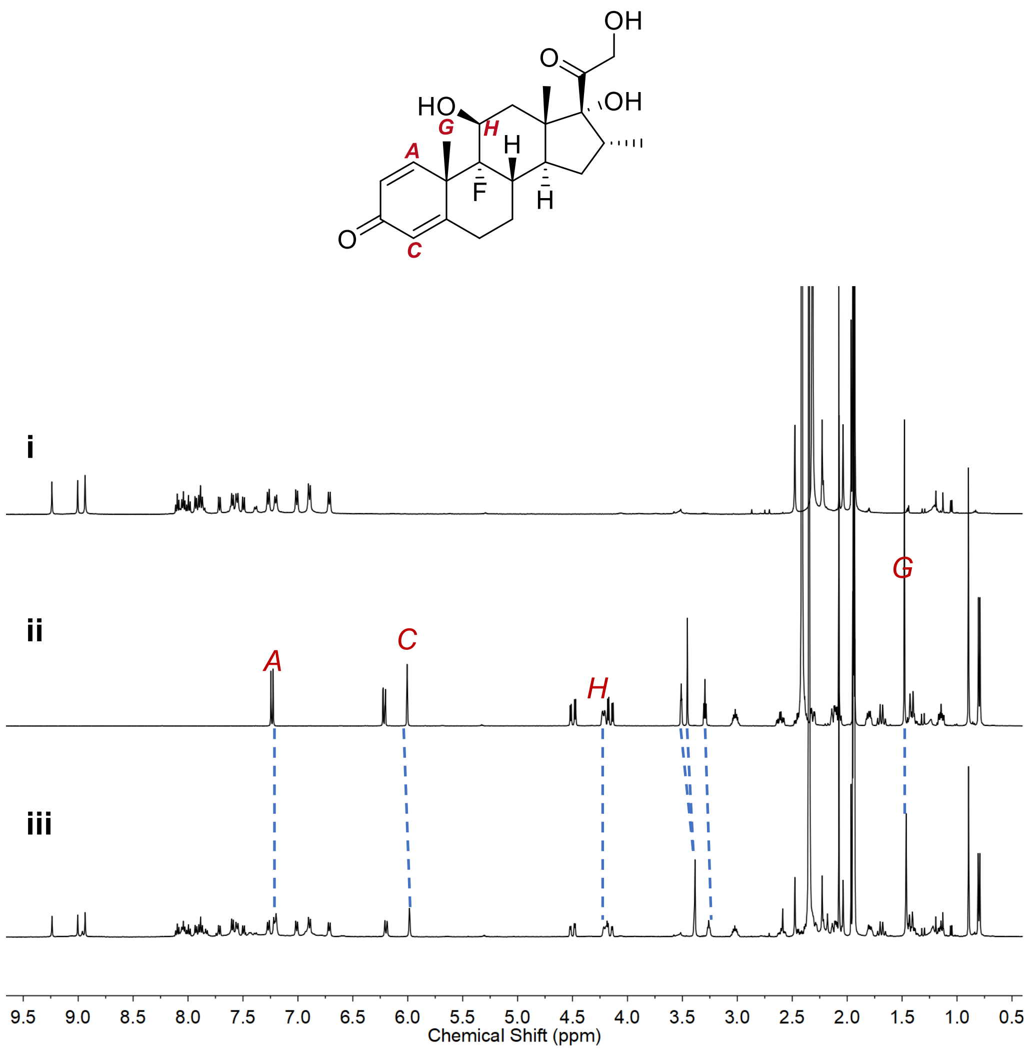

Figure S82: Stacked partial ^{1}H NMR spectra of **i** **1**, **ii** **G10**, **iii** **1** and **G10** (500 MHz, CD_3CN, 253 K). The signals of **G10** that shifted significantly upon encapsulation by **1** are highlighted and assigned based on the 2D NMR data shown in Fig. S113–S118.

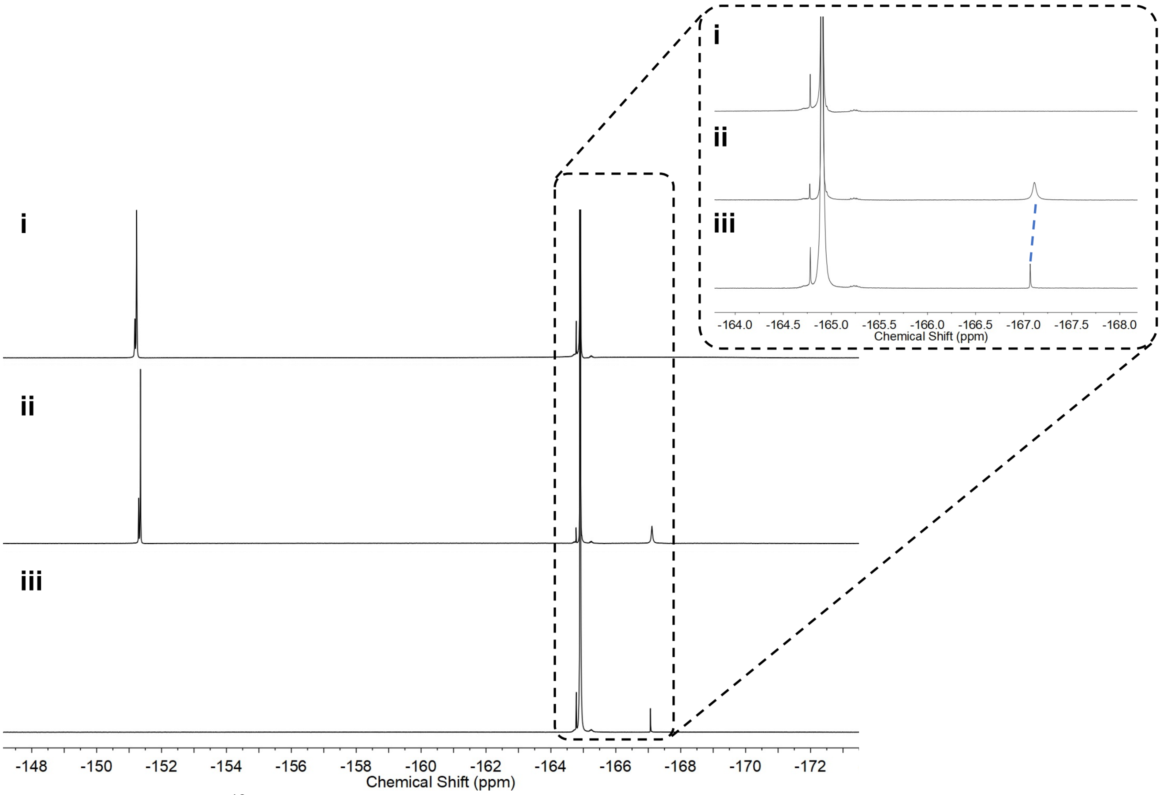

Figure S83: Stacked ^{19}F NMR spectrum of **i 1**, **ii G10⊂1**, **iii** free **G10** (470 MHz, CD_3CN, 253 K). Referenced to hexafluorobenzene. Signals corresponding to **1** and **G10** are labeled. Inset shows the upfield shift of the signal corresponding to **G10** upon encapsulation.

To confirm that the shifts in the signals of **G10** upon addition to **1** were due to interactions between **G10** and cage **1** and not due to trace amounts of Cu^{I} or subcomponent, control experiments were carried out. **G10** was added to the subcomponents required to form cage **1**. No shifts in the signals of **G10** analogous to those seen with cage **1** were observed, indicating that **G10** is interacting with cage **1** not trace amounts of free subcomponent.

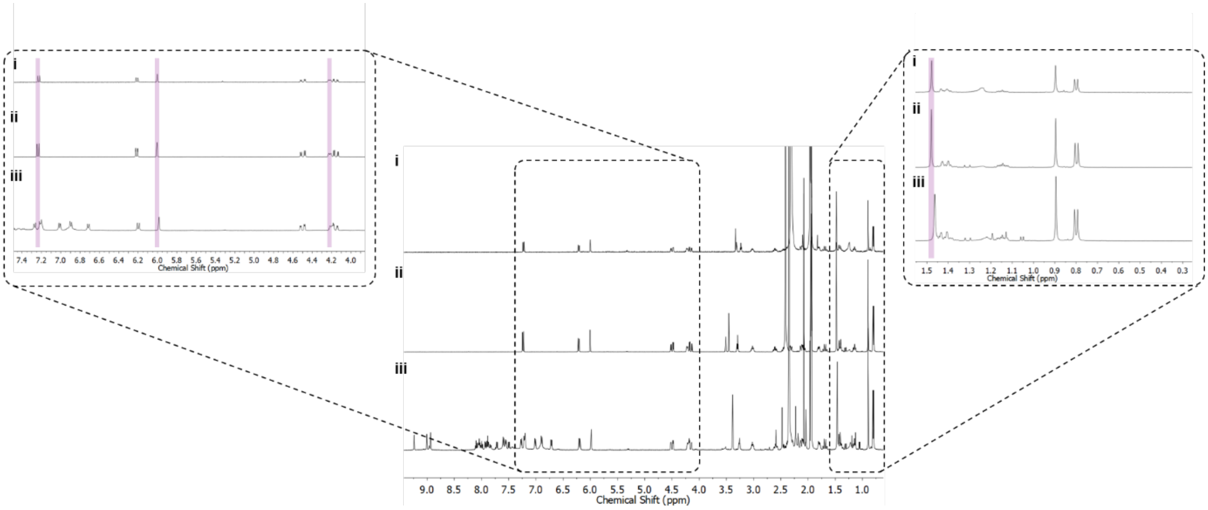

Figure S84: Stacked ^{1}H NMR spectra of **i G10** and $Cu(MeCN)_4BF_4$, **ii** free **G10**, **iii G10⊂1** (500 MHz, CD_3CN, 253 K). Signals of **G10** that shift upon addition to **1** are highlighted. The signals for **i** do not align with those of **iii**.

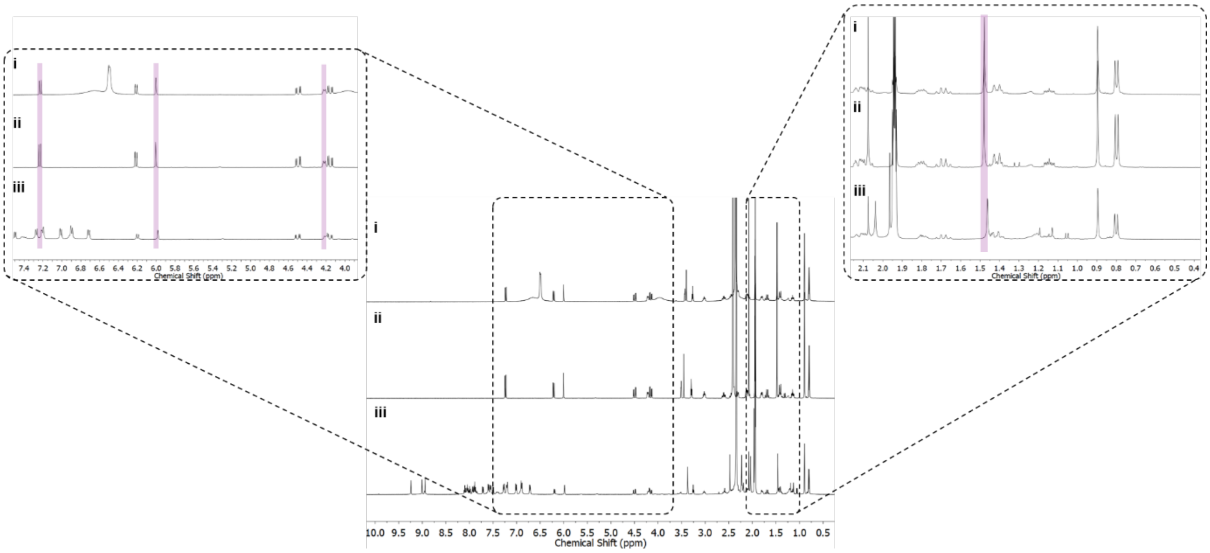

Figure S85: Stacked ^{1}H NMR spectra of **i G10** and **A**, **ii** free **G10**, **iii G10⊂1** (500 MHz, CD_3CN, 253 K). Signals of **G10** that shift upon addition to **1** are highlighted. The signals for **i** do not align with those of **iii**.

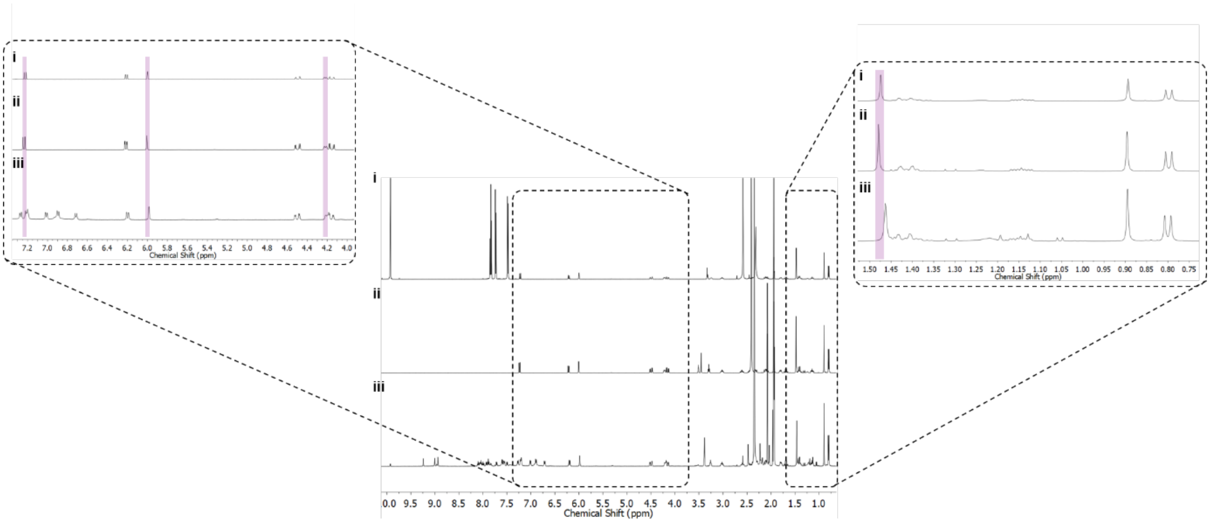

Figure S86: Stacked ^{1}H NMR spectra of **i G10** and **B**, **ii** free **G10**, **iii G10⊂1** (500 MHz, CD_3CN, 253 K). Signals of **G10** that shift upon addition to **1** are highlighted. The signals for **i** do not align with those of **iii**.

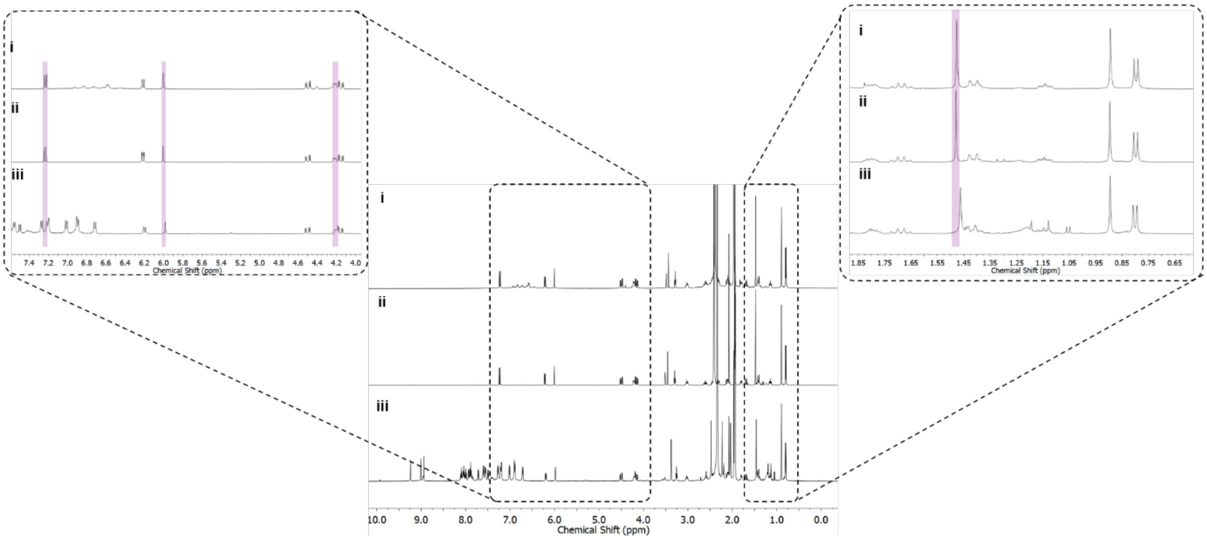

Figure S87: Stacked ^{1}H NMR spectra of **i G10**, $Cu(MeCN)_4BF_4$ and **A**, **ii** free **G10**, **iii G10⊂1** (500 MHz, CD_3CN, 253 K). Signals of **G10** that shift upon addition to **1** are highlighted. The signals for **i** do not align with those of **iii**.

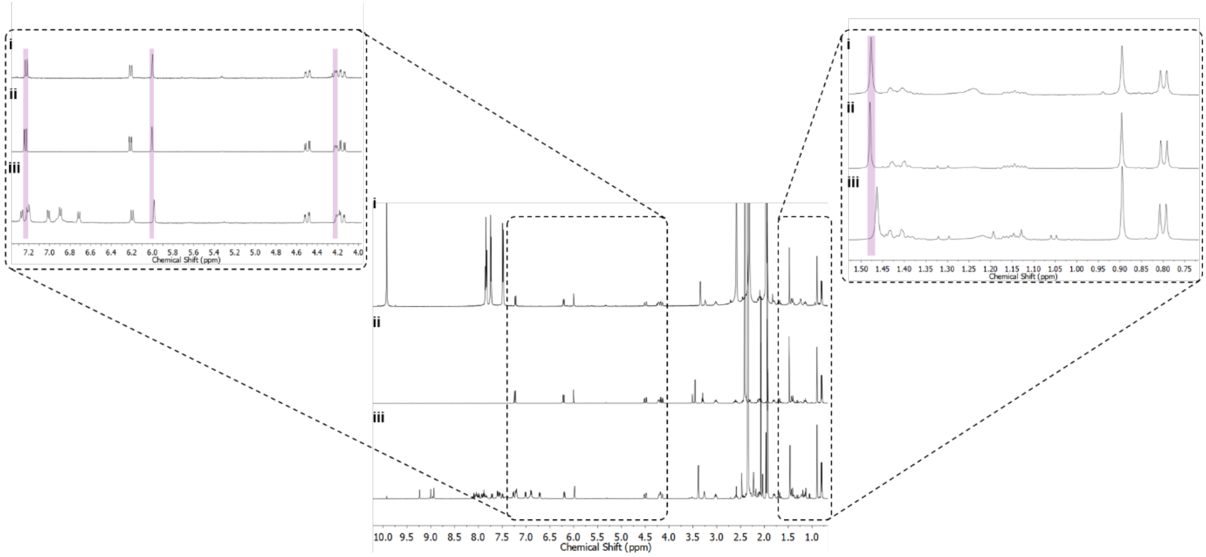

Figure S88: Stacked ^{1}H NMR spectra of **i G10**, $Cu(MeCN)_4BF_4$ and **B**, **ii** free **G10**, **iii G10⊂1** (500 MHz, CD_3CN, 253 K). Signals of **G10** that shift upon addition to **1** are highlighted. The signals for **i** do not align with those of **iii**.

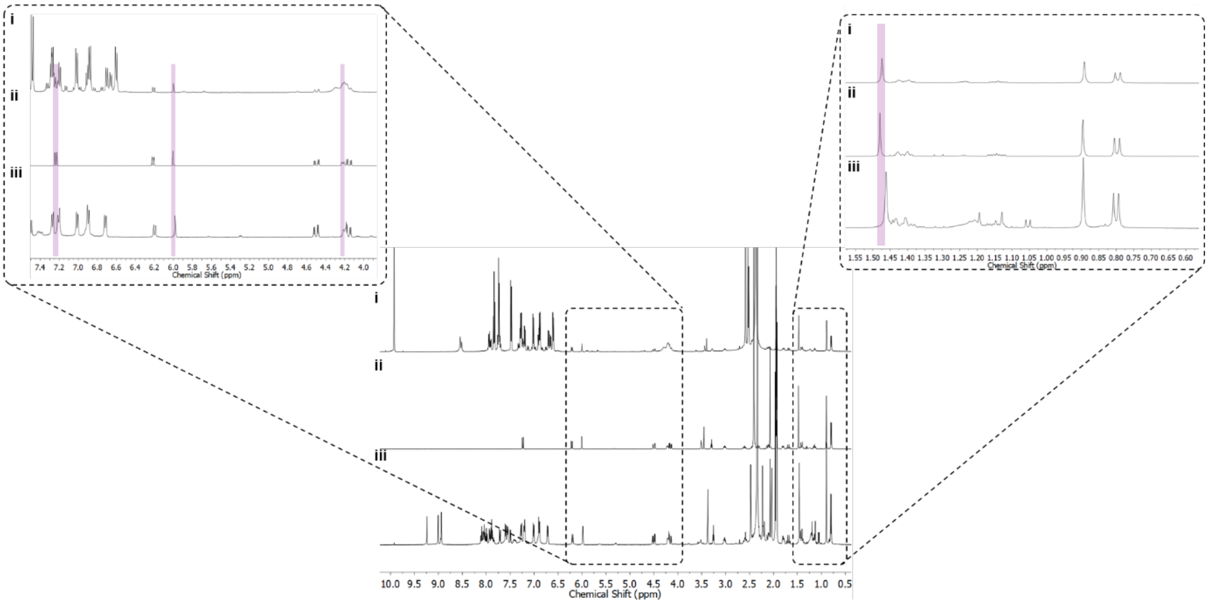

Figure S89: Stacked ^{1}H NMR spectra of **i G10**, **A** and **B**, **ii** free **G10**, **iii G10⊂1** (500 MHz, CD_3CN, 253 K). Signals of **G10** that shift upon addition to **1** are highlighted. Some small shifts were seen between **i** and **ii**, however the signals for **i** do not align with those of **iii**.

To verify that **G4–9** did not bind, the ^{1}H NMR spectra of the free steroid and **1** were compared to those of **1** and the steroid in solution together. For all the non-binding steroids, there were no changes in the multiplicity or chemical shift for either **1** or the steroid.

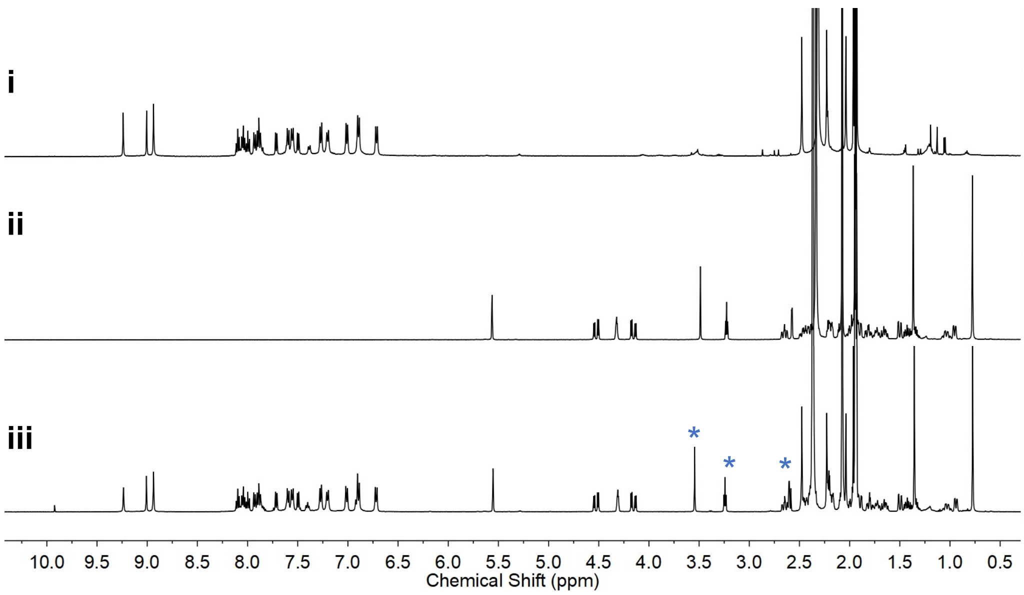

Figure S90: Stacked partial ^{1}H NMR spectra of **i 1**, **ii G4**, **iii 1** and **G4** (500 MHz, CD_3CN, 253 K). The only signals observed to shift in **iii** are the labile O-H protons in **G4**, marked by asterisks.

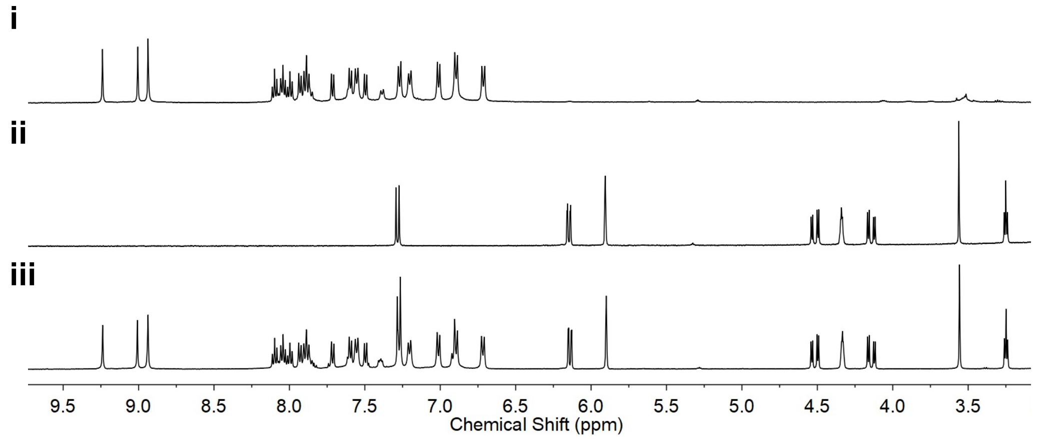

Figure S91: Stacked partial ^{1}H NMR spectra of **i 1**, **ii G5**, **iii 1** and **G5** (500 MHz, CD_3CN, 253 K). There are no shifts or splitting of the signals of **1** or **G5** in **iii**.

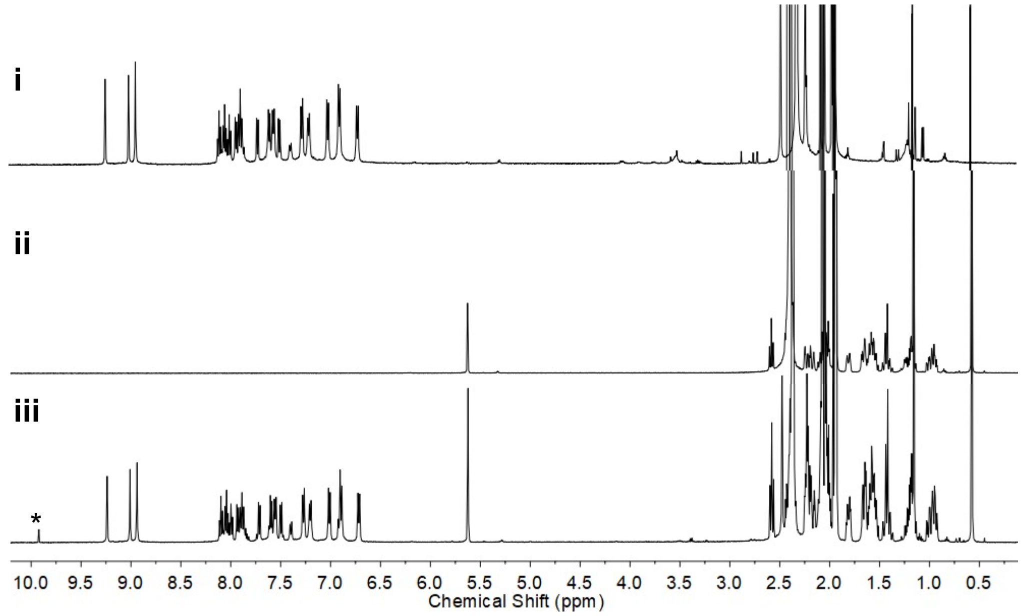

Figure S92: Stacked ^{1}H NMR spectra of **i 1**, **ii G6**, **iii 1** and **G6** (500 MHz, CD_3CN, 253 K). There are no shifts or splitting of the signals of **1** or **G6** in **iii**. A small set of signals corresponding to excess **B** are seen in **iii** (indicated by an asterisk).

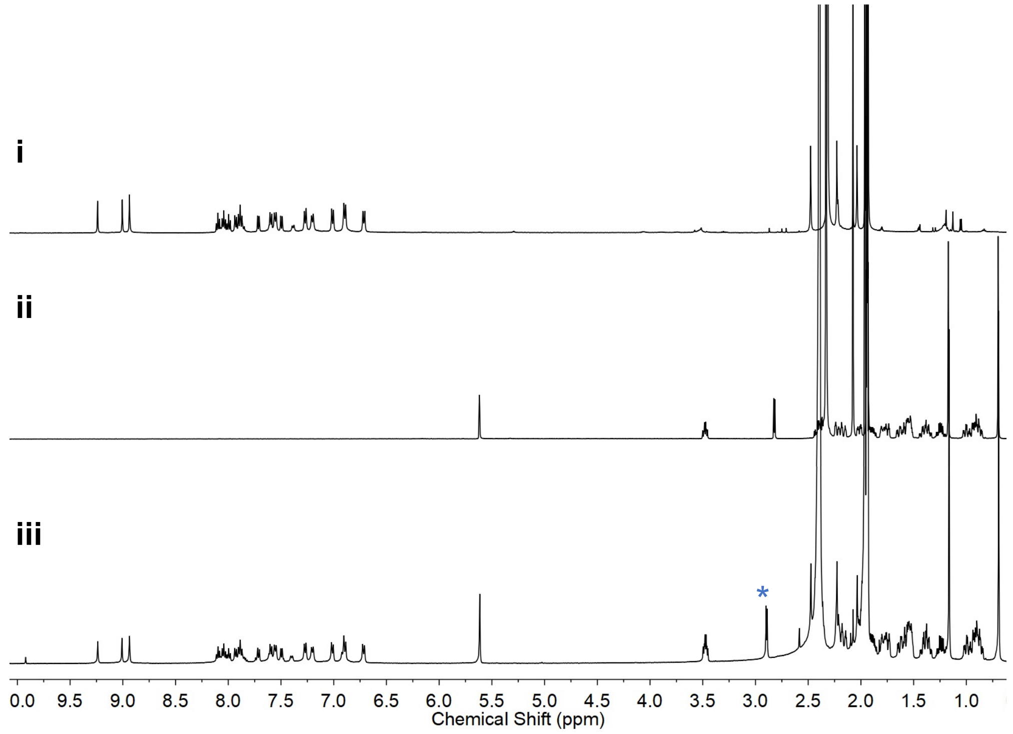

Figure S93: Stacked partial ^{1}H NMR spectra of **i** **1**, **ii** **G7**, **iii** **1** and **G7** (500 MHz, CD_3CN, 253 K). The only signal that shifts in **iii** is the labile O-H proton in **G7**, which is marked by an asterisk.

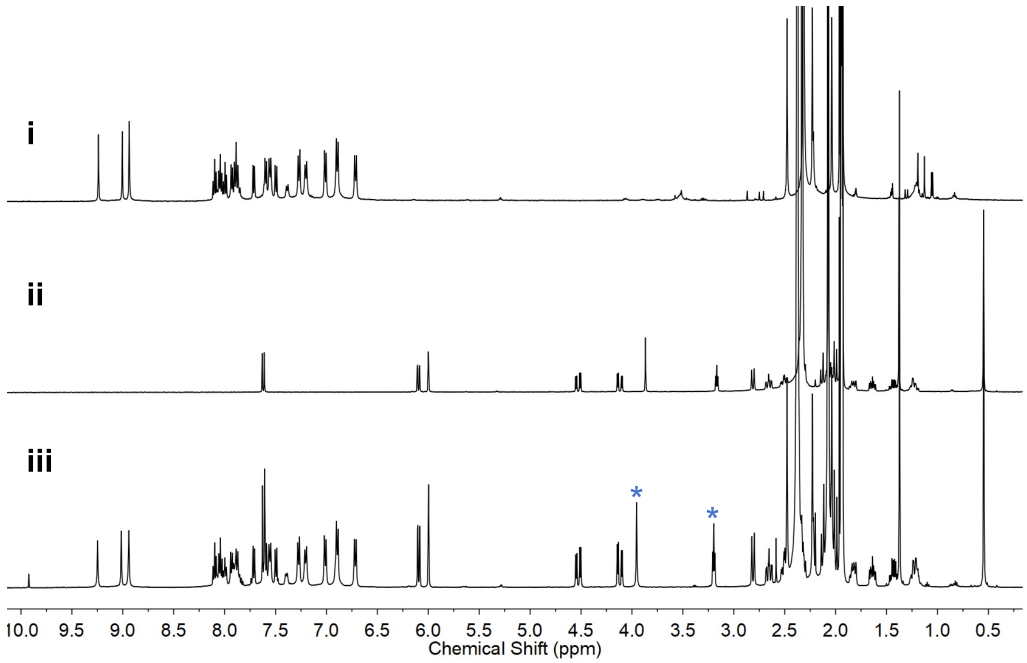

Figure S94: Stacked partial ^{1}H NMR spectra of **i** **1**, **ii** **G8**, **iii** **1** and **G8** (500 MHz, CD_3CN, 253 K). The only signals that shift in **iii** are the labile O-H protons in **G8**, which are marked by asterisks.

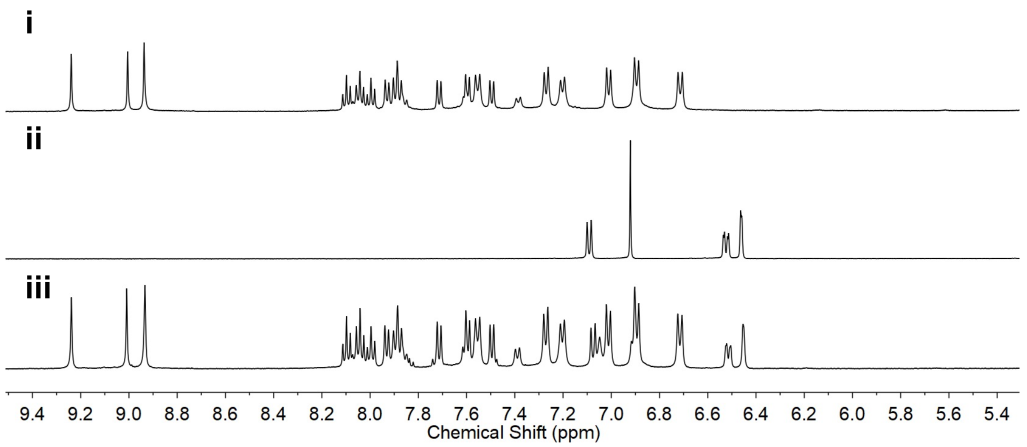

Figure S95: Stacked partial ^{1}H NMR spectra of **i 1**, **ii G9**, **iii 1** and **G9** (500 MHz, CD_3CN, 253 K). There are no shifts or splitting of the signals of **1** or **G9** in **iii**.

S7.2 Characterization of **G11⊂1**

G11 is analogous to **G4**, differing only in the replacement of the proton H-9 by a fluorine atom.

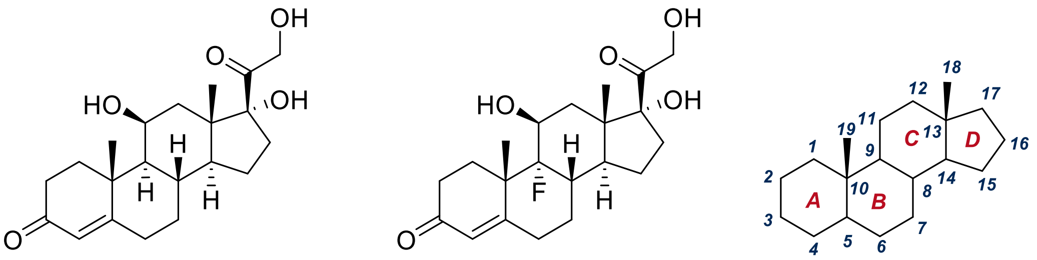

hydrocortisone
G4

fludrocortisone
G11

Figure S96: Structure of **G11** compared to **G4**, with a steroid skeleton labeled according to the IUPAC convention[13].

Addition of **G11** to a solution of **1** in CD_3CN resulted in the appearance of another set of signals alongside the signals corresponding to S_4-**1**. These signals were highly symmetric and so were assigned to the T-**1** diastereomer. These changes in the ^{1}H NMR spectrum of cage **1** indicate an interaction between **1** and **G11**.

As with the binding of **G10**, the conversion was only seen at 253 K and not at 298 K. The ratio of the conversion of S_4-**1** : T-**1** for **G11** was 6.1 : 1.0 by deconvolution of the integrals in the ^{1}H NMR spectrum. This was not changed upon heating overnight.

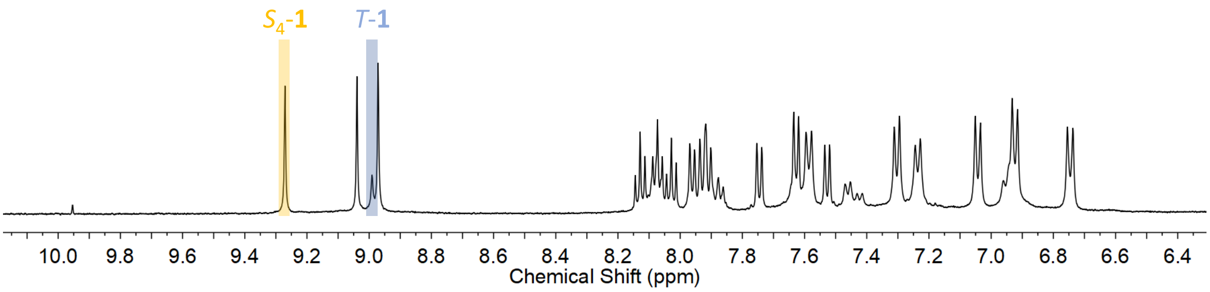

Figure S97: ^{1}H NMR spectrum of **G11⊂1** (500 MHz, CD_3CN, 253 K). The S_4-**1** and *T*-**1** signals used for deconvolution are labeled.

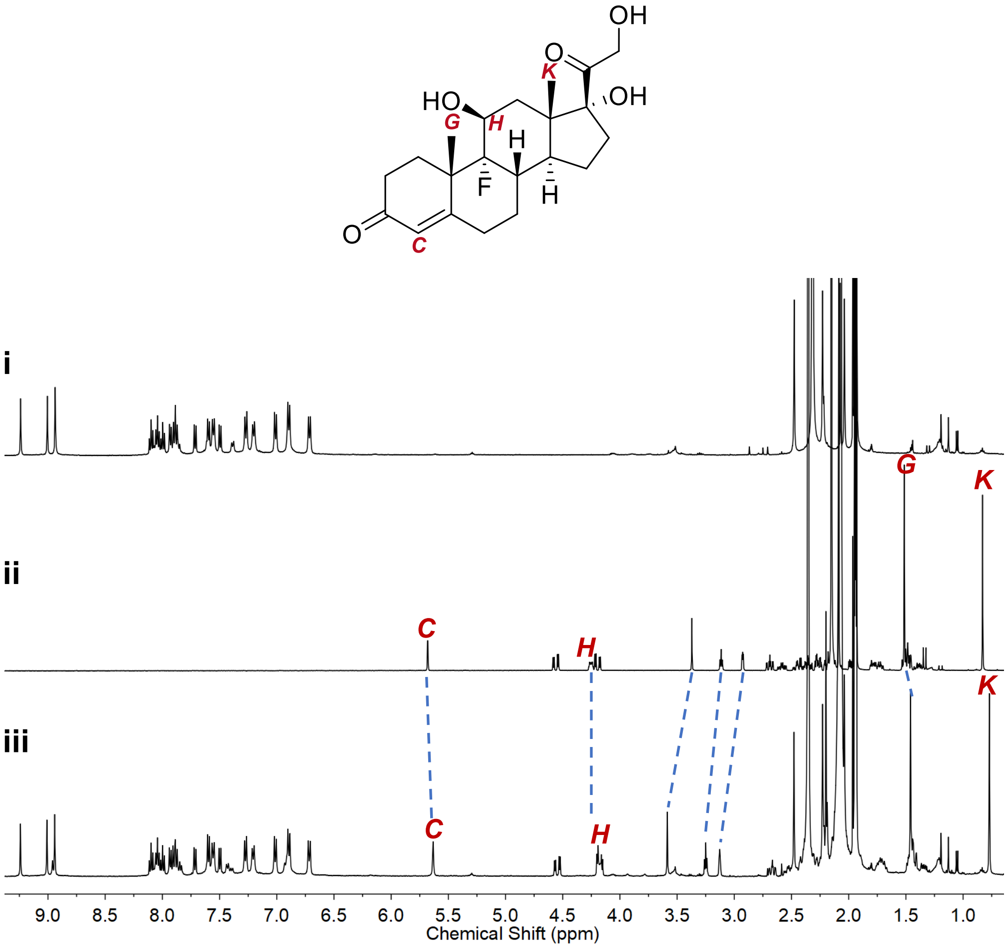

Figure S98: Stacked ^{1}H NMR spectra of **i 1**, **ii** free **G11**, **iii G11⊂1** (500 MHz, CD_3CN, 253 K). ^{1}H signals of **G11** that shift are labeled in **ii** and **iii** with their assignment in **G11**.

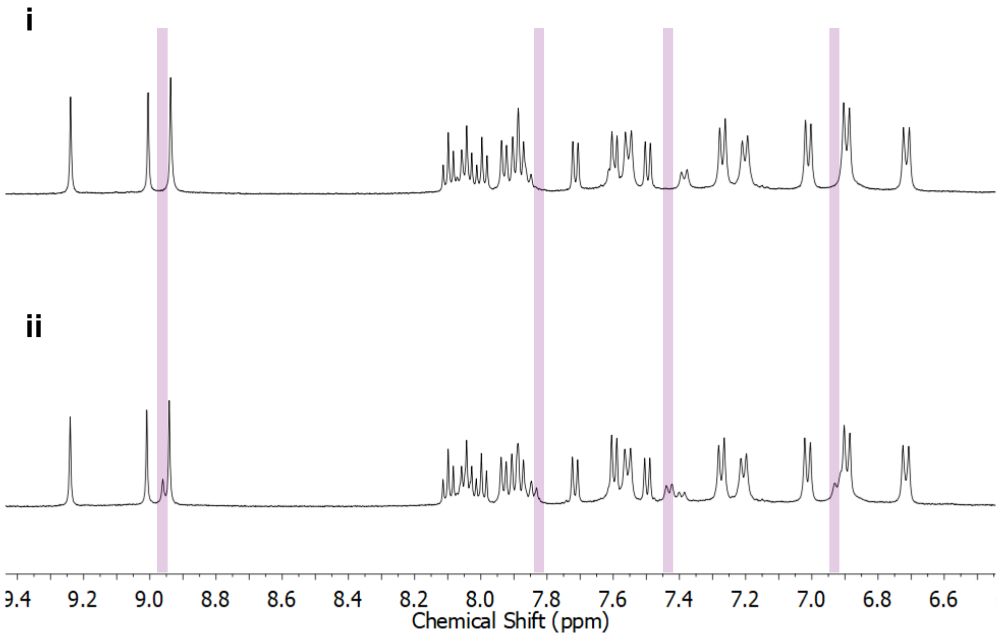

Figure S99: Stacked ^{1}H NMR spectra of the aromatic region of **i 1 ii 1** and **G11** (500 MHz, CD_3CN, 253 K). Some of the new signals of **1**, assigned to the *T*-**1** diastereomer, that appear upon addition of **G11** are highlighted. Due to signal overlap, not all of the signals of *T*-**1** could be identified.

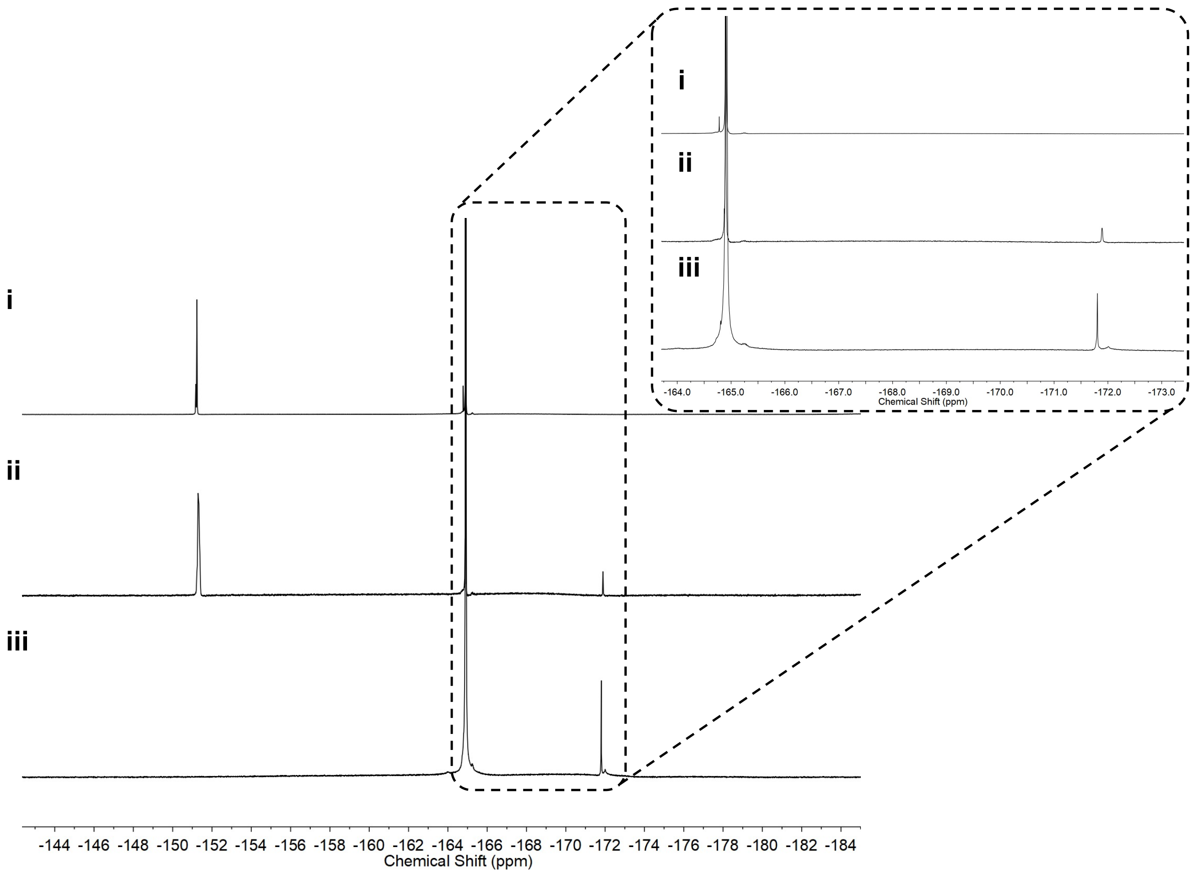

Figure S100: Stacked ^{19}F NMR spectra of **i 1**, **ii G11⊂1**, **iii** free **G11** (470 MHz, CD_3CN, 253 K). Referenced to hexafluorobenzene. Inset shows the upfield shift of the signal corresponding to **G11** upon encapsulation.

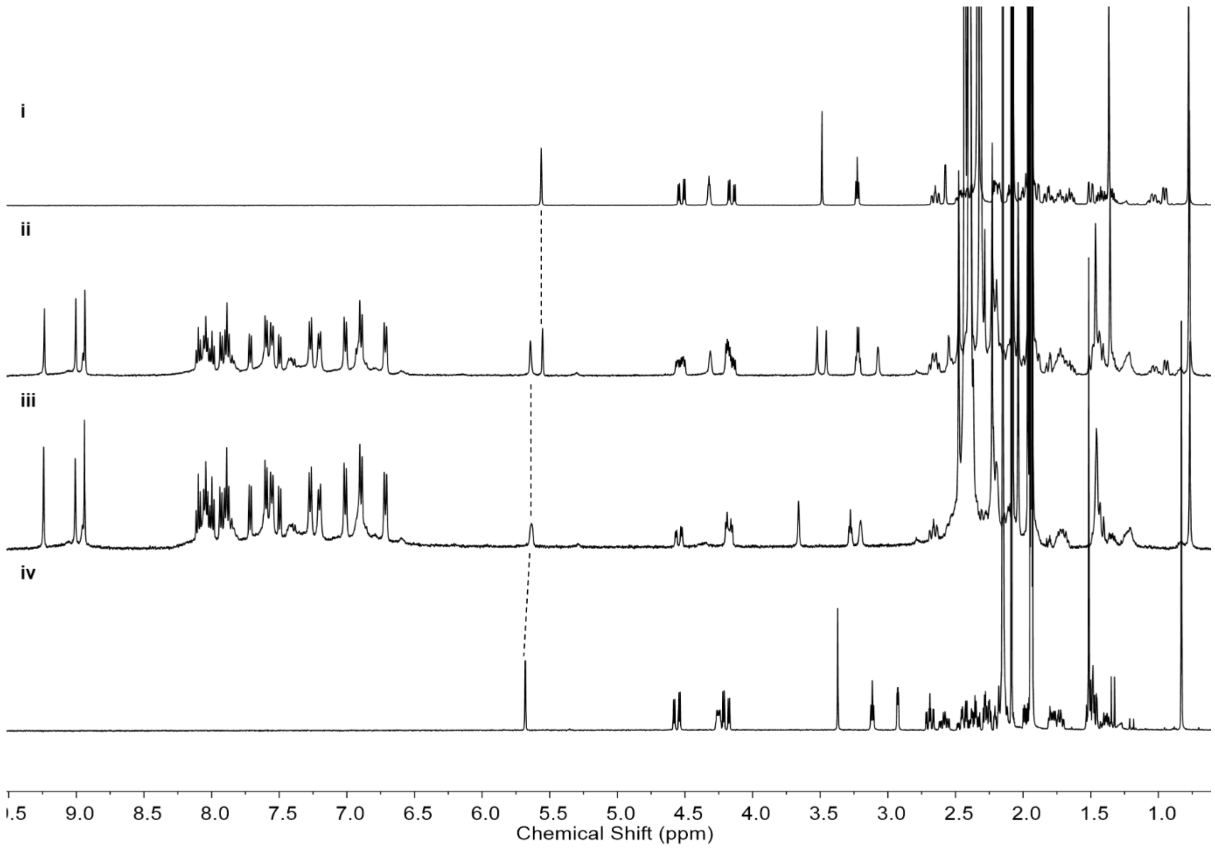

Figure S101: Stacked ^{1}H NMR spectra of **i G4**, **ii G4** and **G11⊂1**, **iii G11⊂1**, **iv G11** (500 MHz, CD_3CN, 253 K).

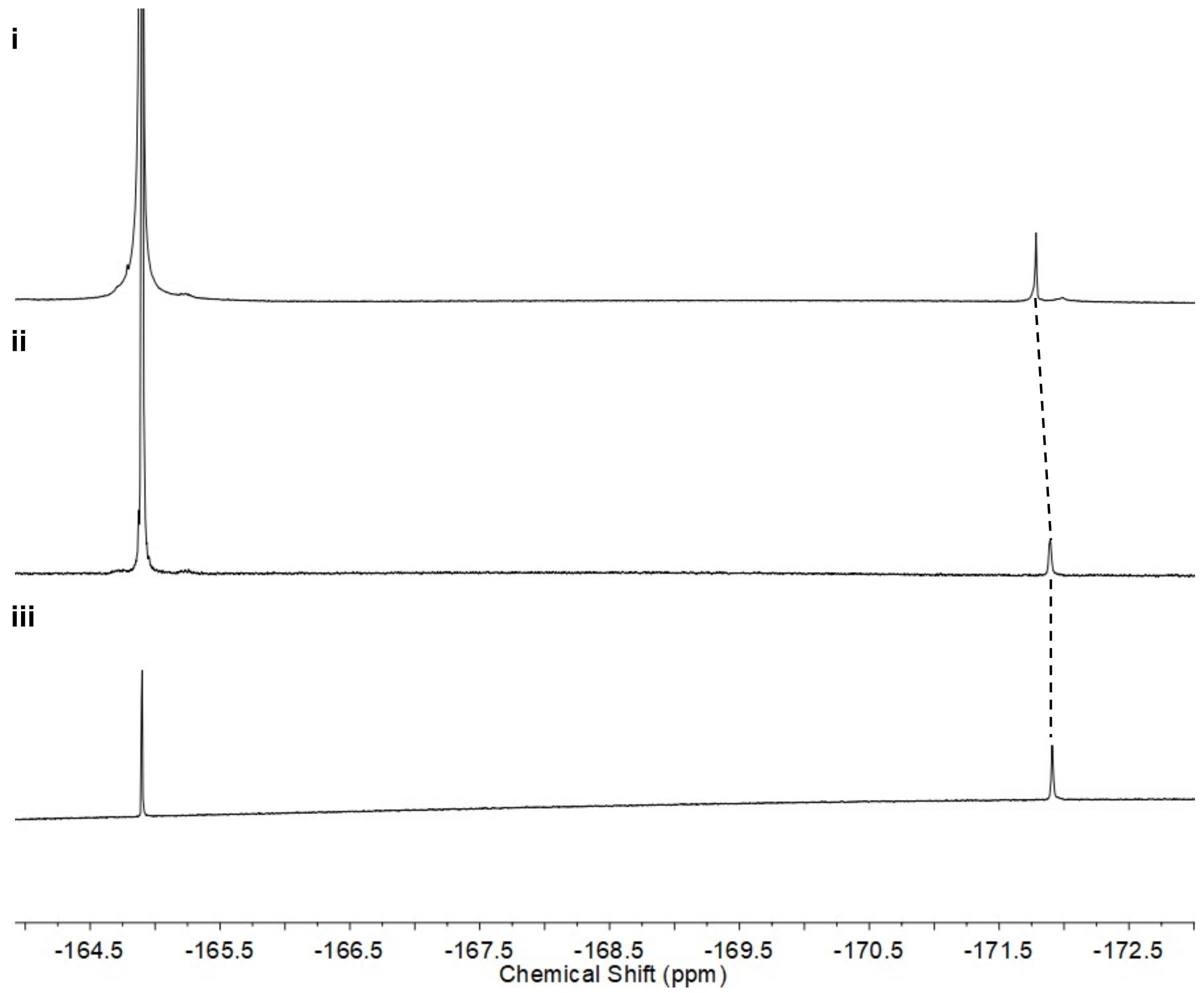

Figure S102: ^{19}F NMR spectra of **i G11**, **ii G11⊂1**, **iii G11⊂1** with **G4** (470 MHz, CD_3CN, 253 K). Referenced to hexafluorobenzene.

To confirm that the shifts in the signals of **G11** upon addition to **1** were due to interactions between **G11** and cage **1** and not due to trace amounts of Cu^I or subcomponent, control experiments were carried out. **G11** was added to the subcomponents required to form cage **1** and no shifts in the signals of **G11** were seen in the ^1H NMR spectra at 253 K.

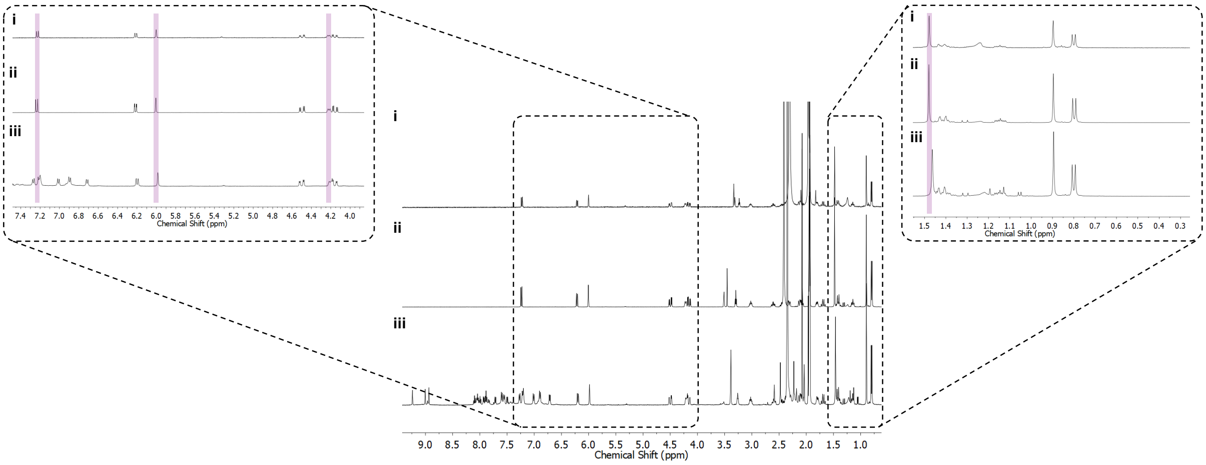

Figure S103: Stacked ^{1}H NMR spectra of **i G11** and $Cu(MeCN)_4BF_4$, **ii** free **G11**, **iii G11⊂1** (500 MHz, CD_3CN, 253 K). Signals of **G11** that shift upon addition to **1** are highlighted. The signals for **i** do not align with those of **iii**.

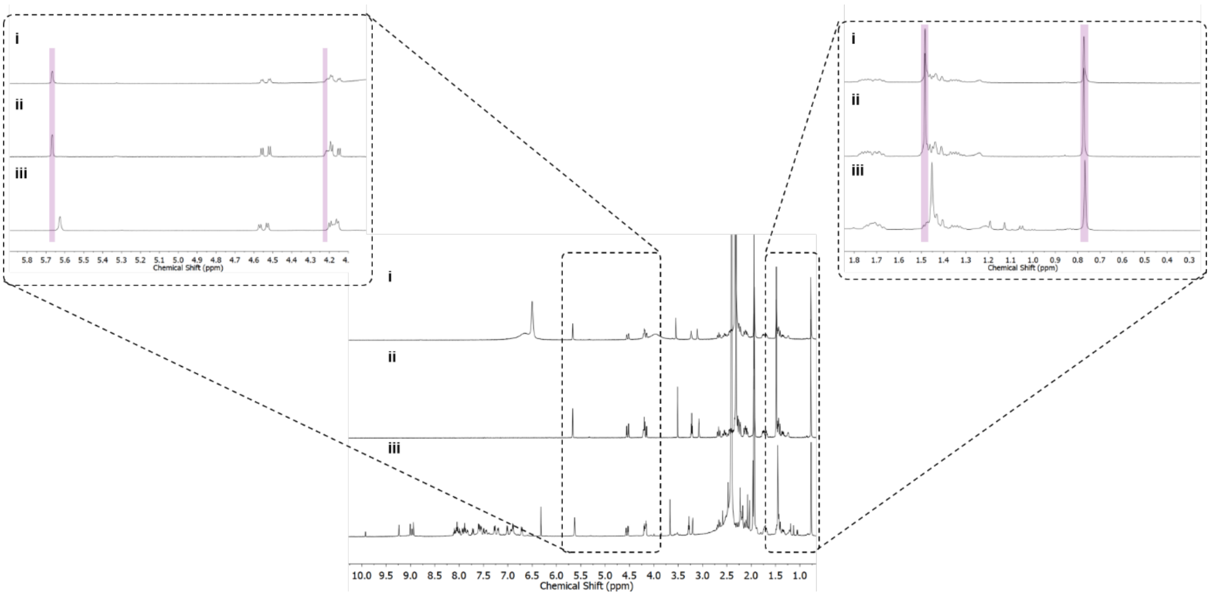

Figure S104: Stacked ^{1}H NMR spectra of **i G11** and **A**, **ii** free **G11**, **iii G11⊂1** (500 MHz, CD_3CN, 253 K). Signals of **G11** that shift upon addition to **1** are highlighted. The signals for **i** do not align with those of **iii**.

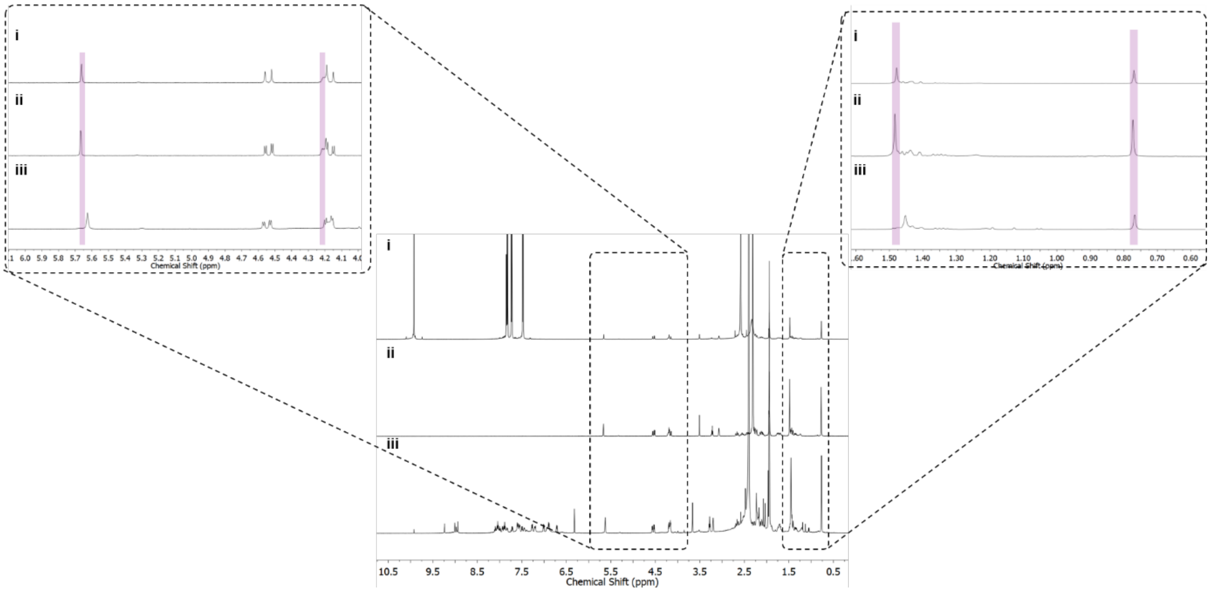

Figure S105: Stacked ^{1}H NMR spectra of **i G11** and **B**, **ii** free **G11**, **iii G11⊂1** (500 MHz, CD_3CN, 253 K). Signals of **G11** that shift upon addition to **1** are highlighted. The signals for **i** do not align with those of **iii**.

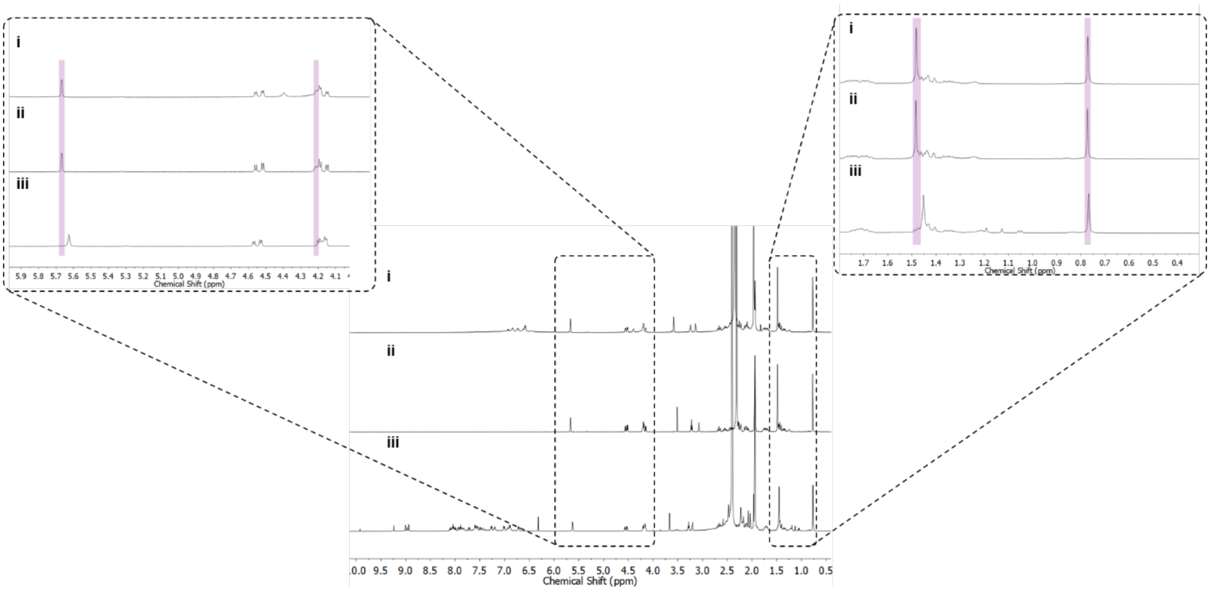

Figure S106: Stacked ^{1}H NMR spectra of **i G11**, $Cu(MeCN)_4BF_4$ and **A**, **ii** free **G11**, **iii G11⊂1** (500 MHz, CD_3CN, 253 K). Signals of **G11** that shift upon addition to **1** are highlighted. The signals for **i** do not align with those of **iii**.

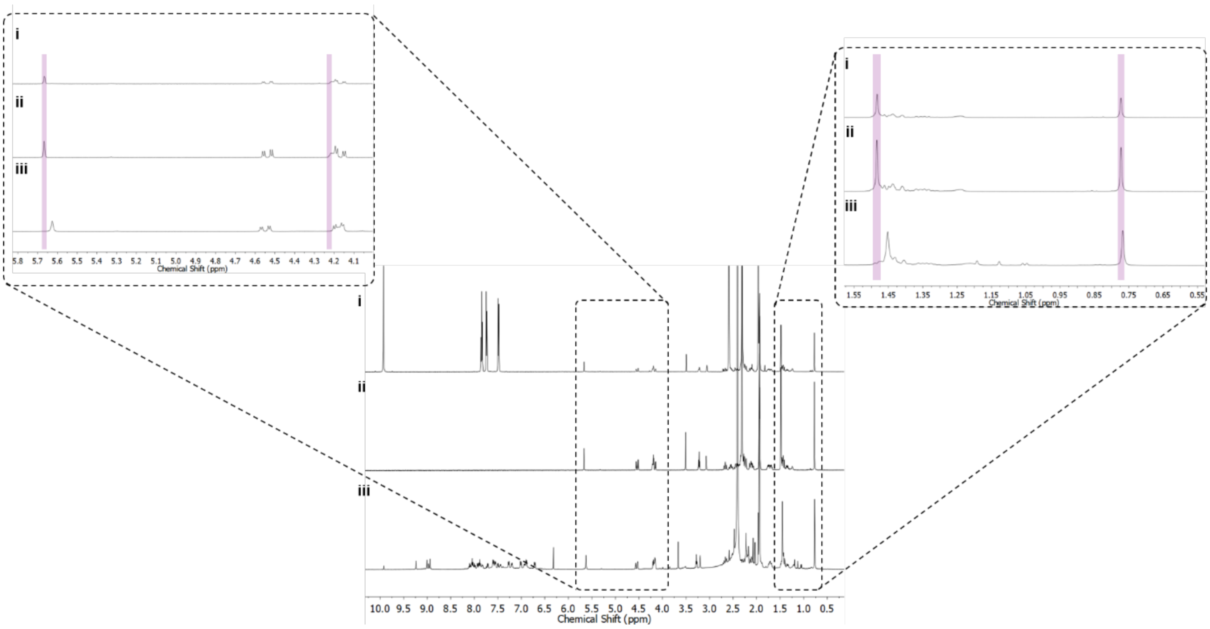

Figure S107: Stacked ^{1}H NMR spectra of **i G11**, $Cu(MeCN)_4BF_4$ and **B**, **ii** free **G11**, **iii G11⊂1** (500 MHz, CD_3CN, 253 K). Signals of **G11** that shift upon addition to **1** are highlighted. The signals for **i** do not align with those of **iii**.

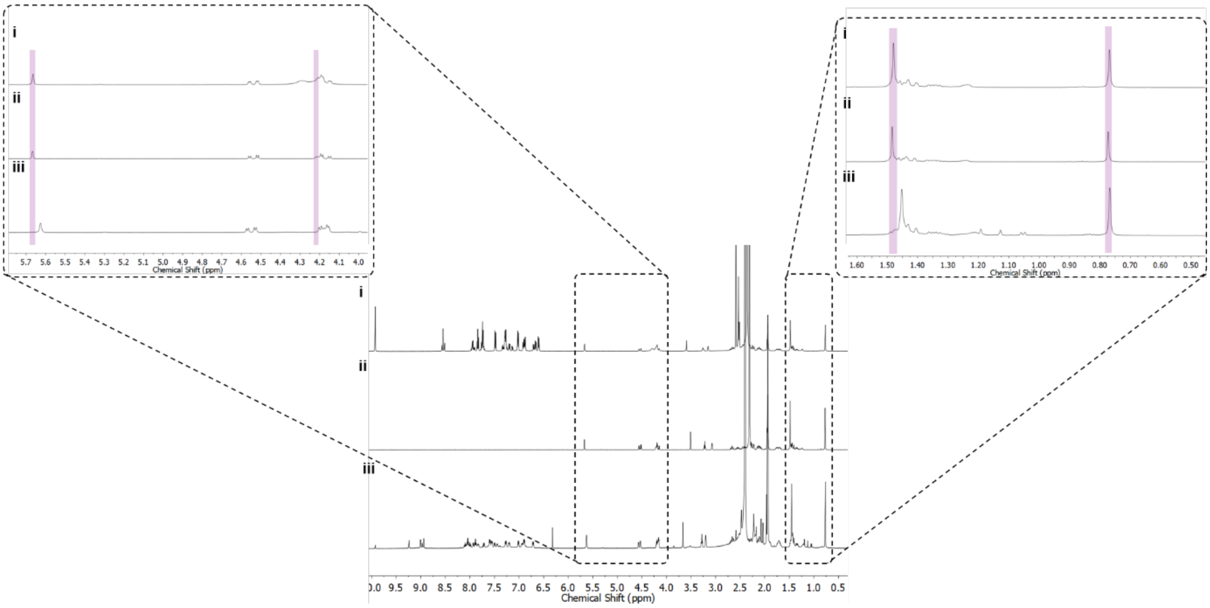

Figure S108: Stacked ^{1}H NMR specta of **i G11**, **A** and **B**, **ii** free **G11**, **iii G11⊂1** (500 MHz, CD_3CN, 253 K). Signals of **G11** that shift upon addition to **1** are highlighted. The signals for **i** do not align with those of **iii**.

S7.3 Binding mode of **G11⊂1**

To determine whether the central pocket of **1** was playing a role in the binding of the fluorinated steroids, **G11** was added to suit[4]ane **G3⊂*T*-1** at 253 K. Shifts in the ^{1}H and ^{19}F NMR signals (Figs. S109 and S110) of **G11** compared to free **G11** were observed. These shifts were similar to those seen for the complex **G11⊂1**, suggesting a similar mode of interaction between **G11** and the suit[4]ane as observed between **G11** and cage **1**. This observation suggested that the

central pocket of **1** is not the binding site for **G11**, implicating instead that the deep clefts between ligands.

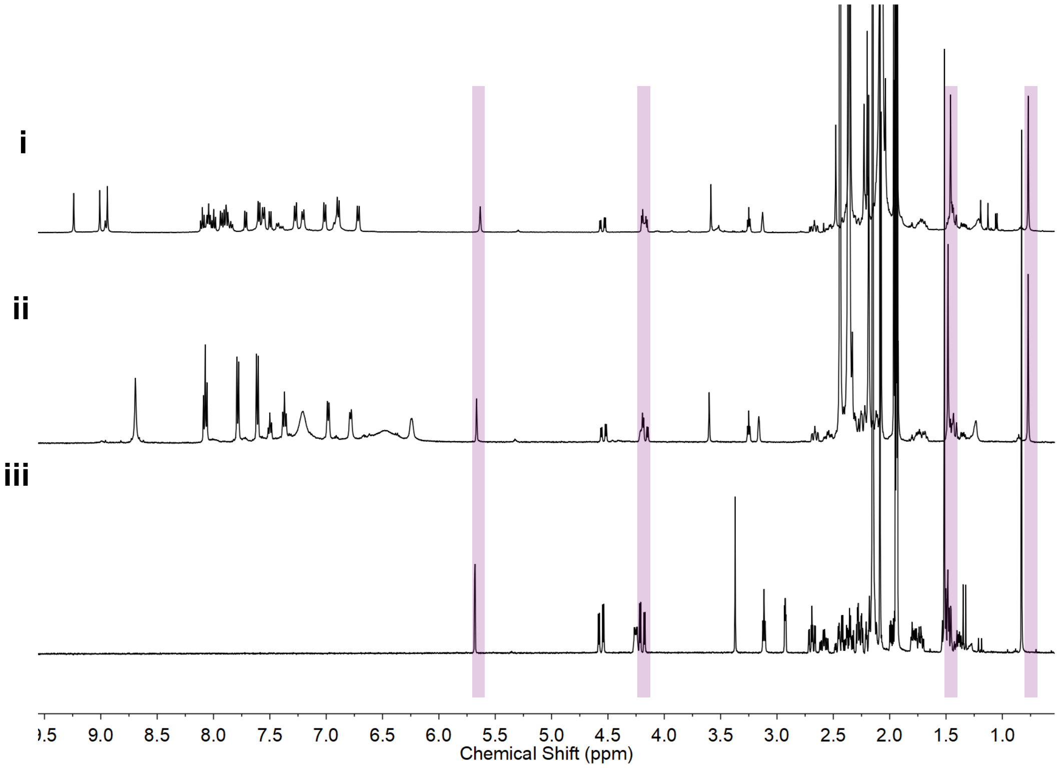

Figure S109: Stacked ^{1}H NMR spectra of **i G11⊂1**, **ii G3⊂*T*-1** and **G11**, and **iii** free **G11** (500 MHz, CD_3CN, 253 K). Shifts in the signals of **G11** upon addition of **1** or **G3⊂*T*-1** are highlighted.

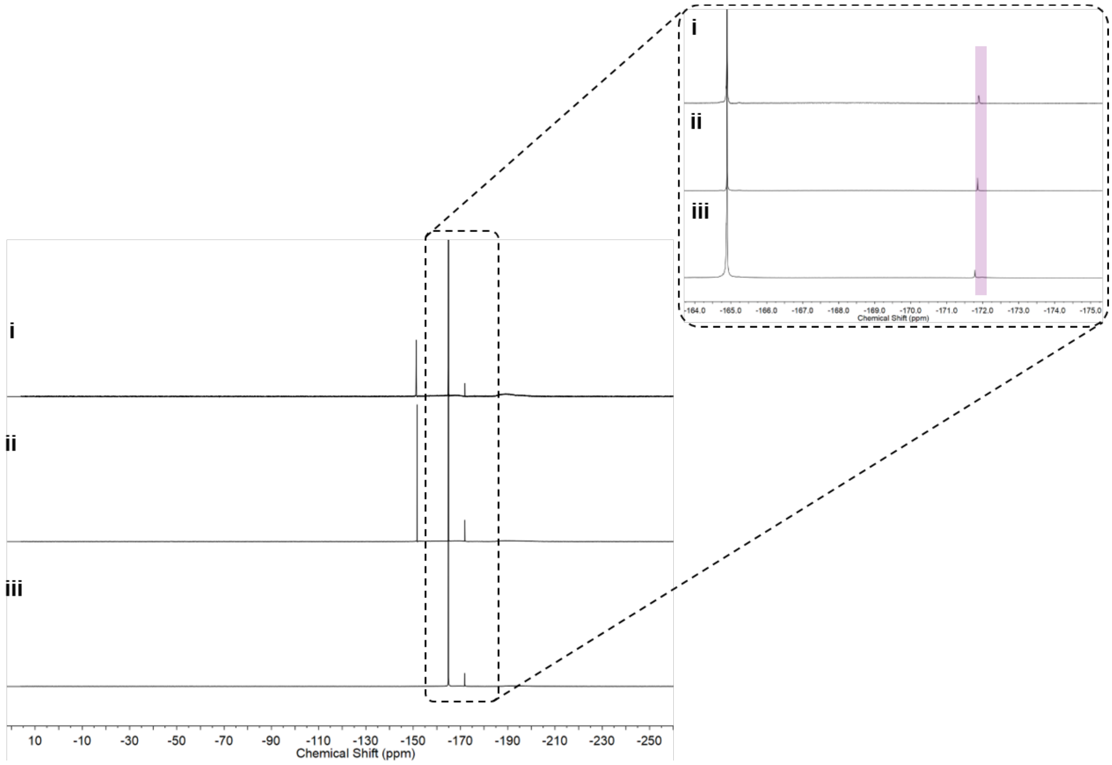

Figure 110: Stacked ^{19}F NMR spectra of **i** of **i G11⊂1**, **ii G3⊂*T*-1** and **G11**, and **iii** free **G11** (470 MHz, CD3CN, 253 K). Spectra are referenced to hexafluorobenzene. The shift in the signal of **G11** upon addition of **G3⊂*T*-1** or **1** is highlighted.

1D selective ^{1}H-^{19}F heteronuclear Overhauser effect spectroscopy (HOESY) NMR experiments were carried out to determine which proton environments on cage **1** were spatially close to the fluorine on **G11**. The spectrum shows correlations between the fluorine of **G11** and protons H_f and H_g of the minor *T*-**1** diastereomer of **G11⊂1** (Fig S112), indicating the **G11** is binding in the clefts across of the closed faces of *T*-**1**.

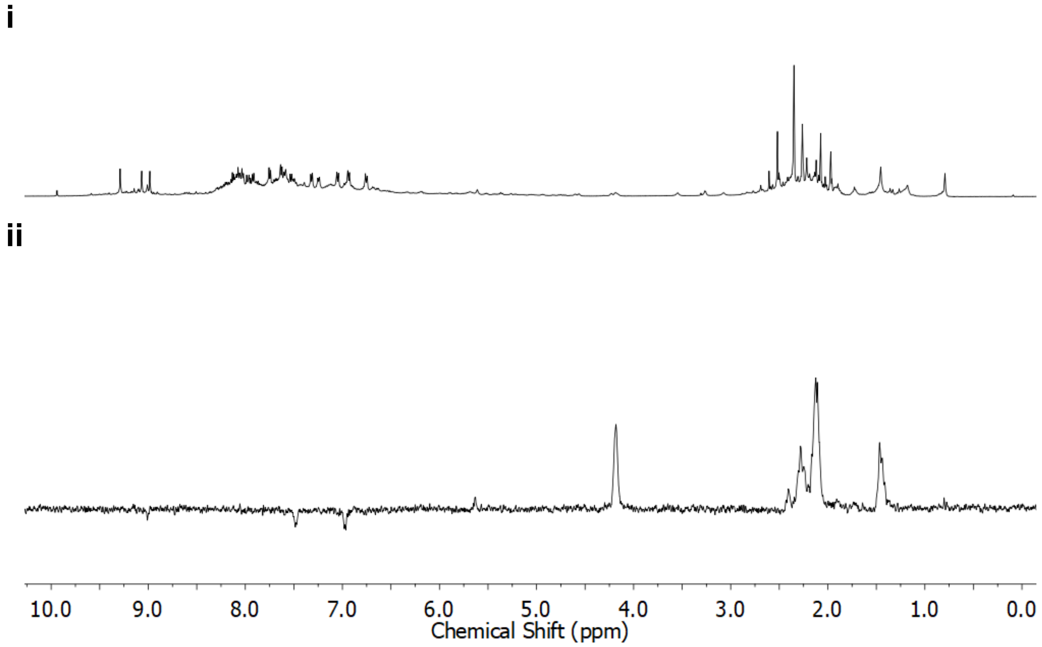

Figure S111: **i** ^{1}H NMR spectrum of **G11⊂1** **ii** 1D selective ^{1}H-^{19}F HOESY NMR spectrum of **G11⊂1**, with the selective excitation of the fluorine of **G11** (470 MHz, CD_3CN, 298 K).

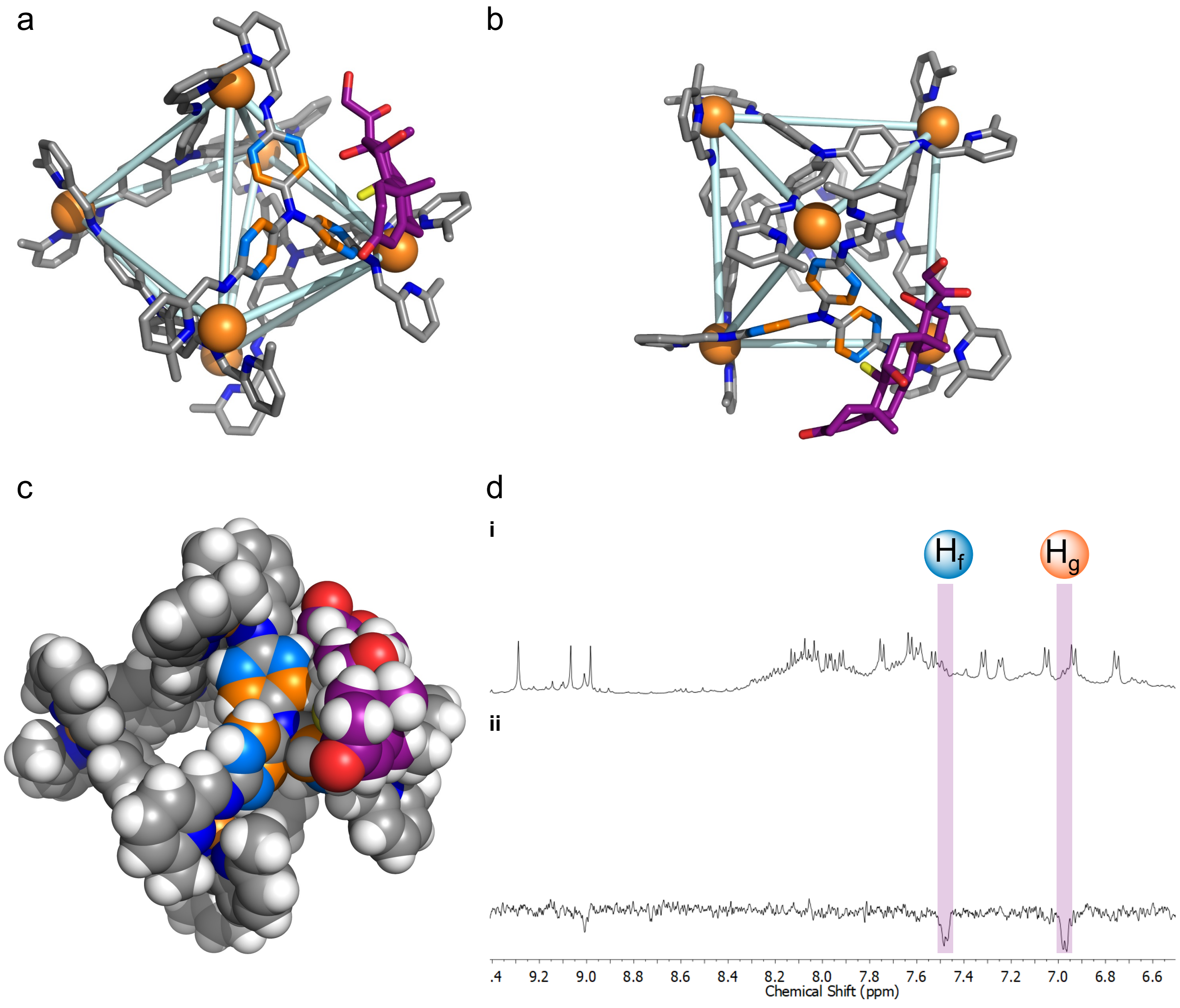

Figure S112: Illustration showing the potential binding mode of **G11**[14] with cage *T*-**1**. The fluorine atom in **G11** is highlighted in yellow, the carbon atoms of **G11** are purple, whilst the protons of **1** spatially close to the fluorine are highlighted in orange and blue. (a) Illustration of a potential binding mode of **G11**⊂*T*-**1**. (b) Illustration of a potential binding mode of **G11**⊂*T*-**1** shown down the S_4 axis. (c) Space-filling view of a potential binding mode of **G11**⊂*T*-**1**. (d) **i** ^{1}H NMR spectrum of **G11**⊂**1** **ii** 1D selective ^{1}H-^{19}F HOESY NMR spectrum of **G11**⊂**1**, with the selective excitation of the fluorine of **G11** (470 MHz, CD_3CN, 298 K). Due to the high symmetry of the *T*-**1** diastereomer, there are 6 equivalent H_f and H_g protons on each face of *T*-**1** that orient towards **G11**. The crystal structure of **G11** was reproduced with permission of the International Union of Crystallography, reference 14. Copyright 1972 *Acta Cryst. B*.

Due to the poor solubility of **G10** and **G11** in MeCN, it was not possible to determine association constants for **G10**⊂**1** and **G11**⊂**1**.

S7.4 ^{1}H NMR assignments of fluorinated steroids in CD_3CN

S7.4.1 **G10**

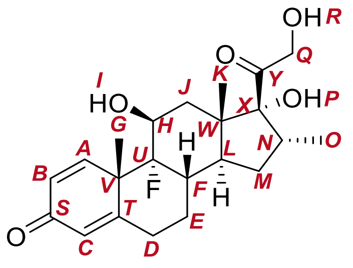

δ_H (700 MHz, CD_3CN, 298 K) 7.24 (d, *J* = 10.2 Hz, 1H, H_A), 6.22 (dd, *J* = 10.1 Hz, 1.9 Hz, 1H, H_B), 6.01 (s, 1H, H_C), 4.52 (dd, *J* = 19.6 Hz, 5.3 Hz, 1H, H_{Q1}), 4.28–4.25 (m, 1H, H_H), 4.18 (dd, *J* = 19.6 Hz, 5.5 Hz, 1H, H_{Q2}), 3.19 (s, 1H, H_P), 3.18 (m, 1H, H_I), 3.13 (t, *J* = 5.3 Hz, 1H, H_R), 3.04 (dqd, *J* = 11.1, 7.5, 4.3 Hz, 1H, H_N), 2.64 (tdd, *J* = 13.8 Hz, 6.2 Hz, 1.6 Hz, 1H , H_{D1}), 2.44 (dtd, *J* = 30.1 Hz, 11.9 Hz, Hz, 1H, H_F), 2.34 (ddd, *J* = 6.9 Hz, 2.6 Hz, 0.8 Hz, 1H, H_{D2}), 2.19–2.16 (m, 1H, H_{J1}), 2.13–2.09 (m, 1H, H_L), 1.85–1.82 (m, 1H, H_{E1}), 1.74 (q, *J* = 6.2 Hz, 1H, H_{M2}), 1.51 (s, 3H, H_G), 1.48–1.42 (m, 2H, H_{E2} and H_{J2}), 1.19 (dq, *J* = 6.1 Hz, 2.0 Hz, 1H, H_{M1}), 0.95 (s, 1H, H_K), 0.85 (d, *J* = 3.6 Hz, 3H, H_O).

δ_C (176 MHz, CD_3CN, 298 K) 212.7 (C_Y), 186.8 (C_S), 168.0 (C_T), 153.4 (C_A), 130.1 (C_B), 125.3 (C_C), 72.4 (d, *J* = 18.9 Hz, C_H), 68.1 (C_Q), 49.2 (C_X), 44.8 (C_L), 37.0 (C_J), 36.8 (C_N), 35.0 (d, *J* = 9.6 Hz, C_F), 33.0 (C_M), 31.6 (C_D), 28.4 (C_E), 23.7 (d, *J* = 2.9 Hz, C_G), 17.4 (C_K), 15.3 (C_O).

C_T, C_U and C_V could not be unambiguously assigned.

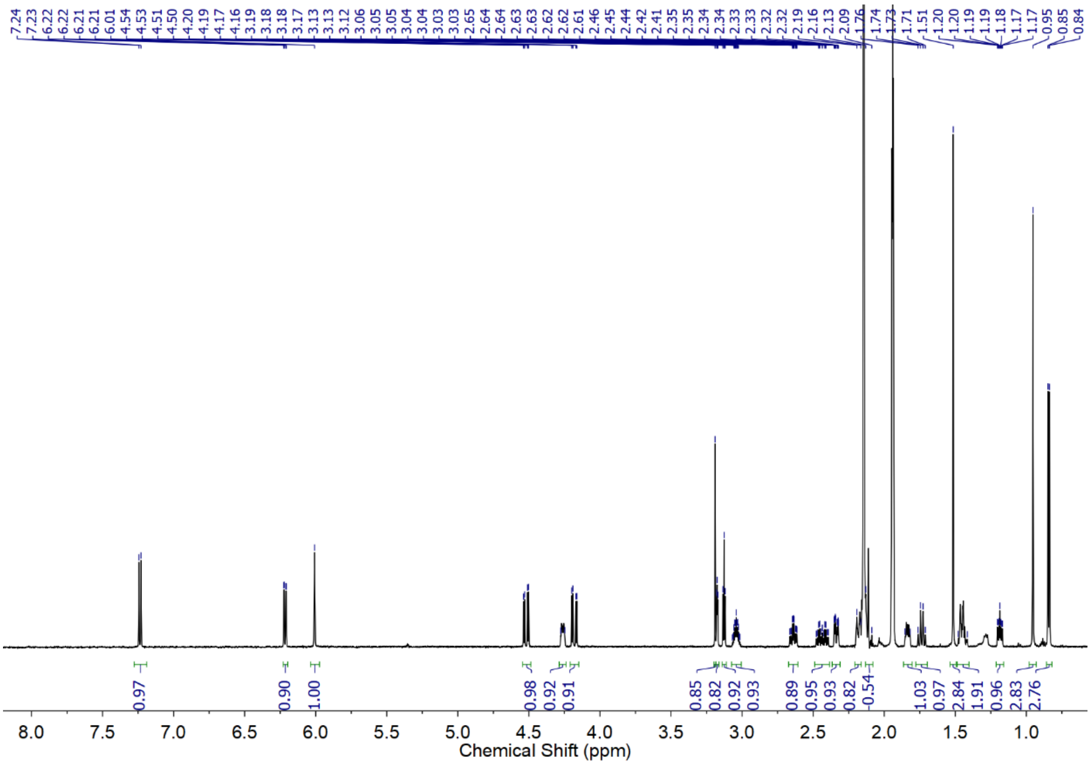

Figure S113: ^{1}H NMR spectrum of **G10** (700 MHz, CD_3CN, 298 K).

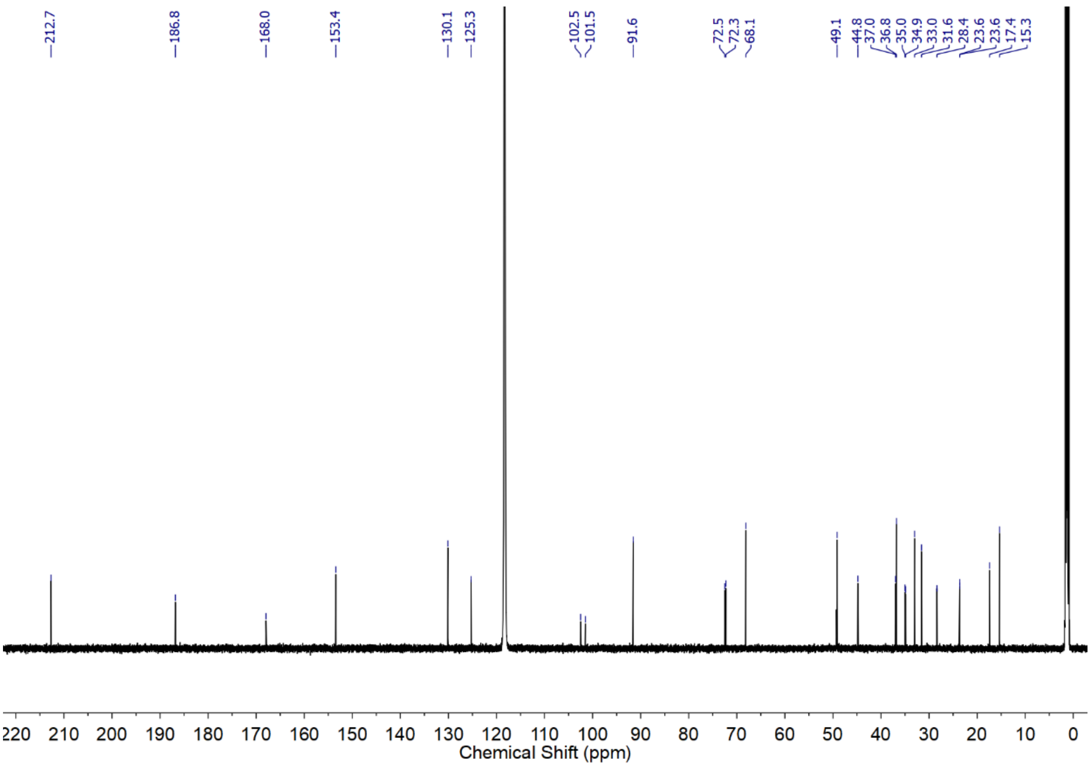

Figure S114: ^{13}C NMR spectrum of **G10** (176 MHz, CD_3CN, 298 K).

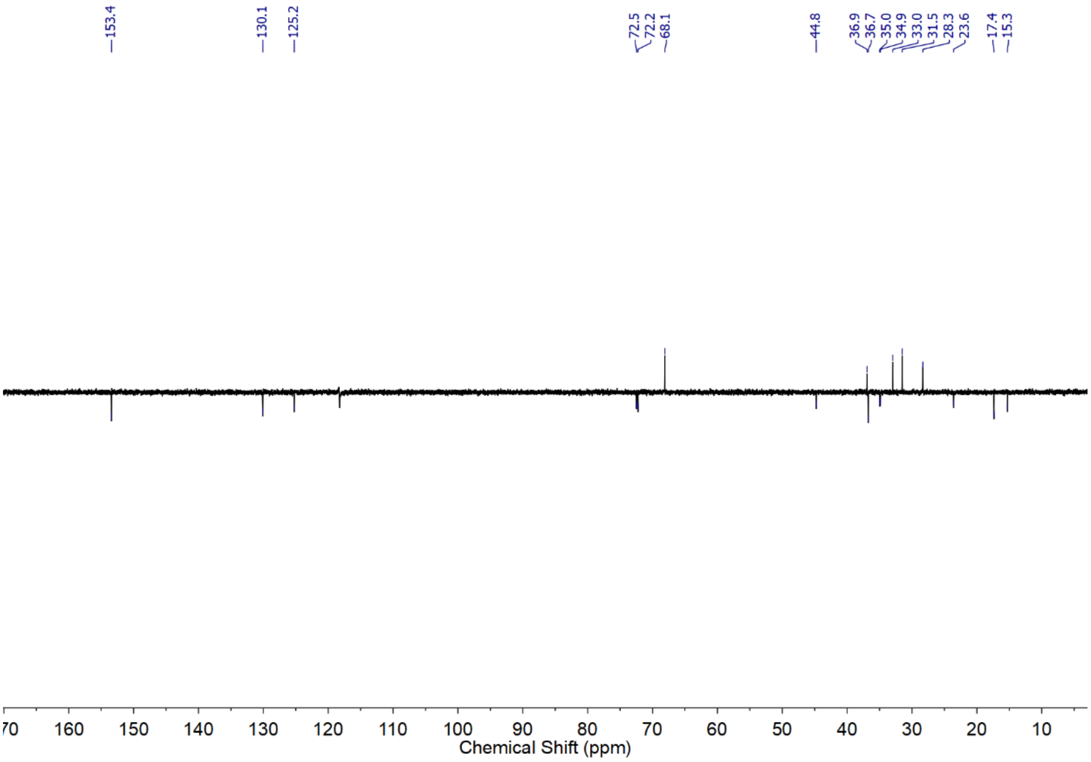

Figure S115: DEPT-135 NMR spectrum of **G10** (176 MHz, CD_3CN, 298 K).

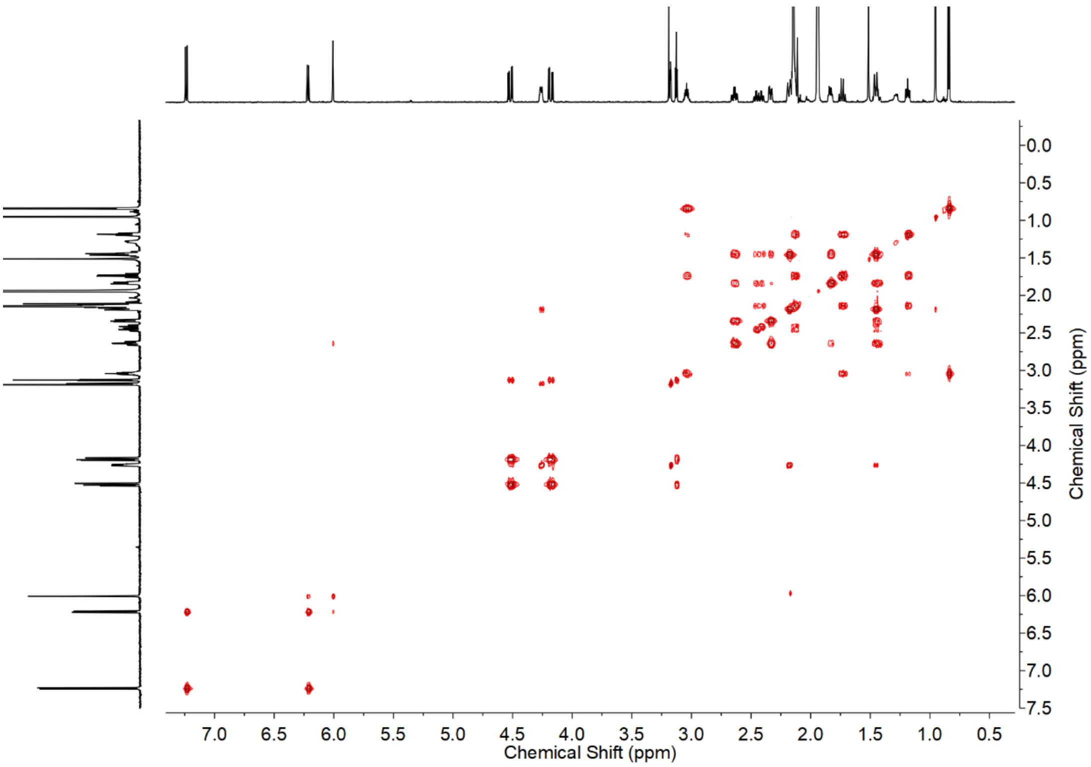

Figure S116: ^{1}H-^{1}H DQF-COSY NMR spectrum of **G10** (700 MHz, CD_3CN, 298 K).

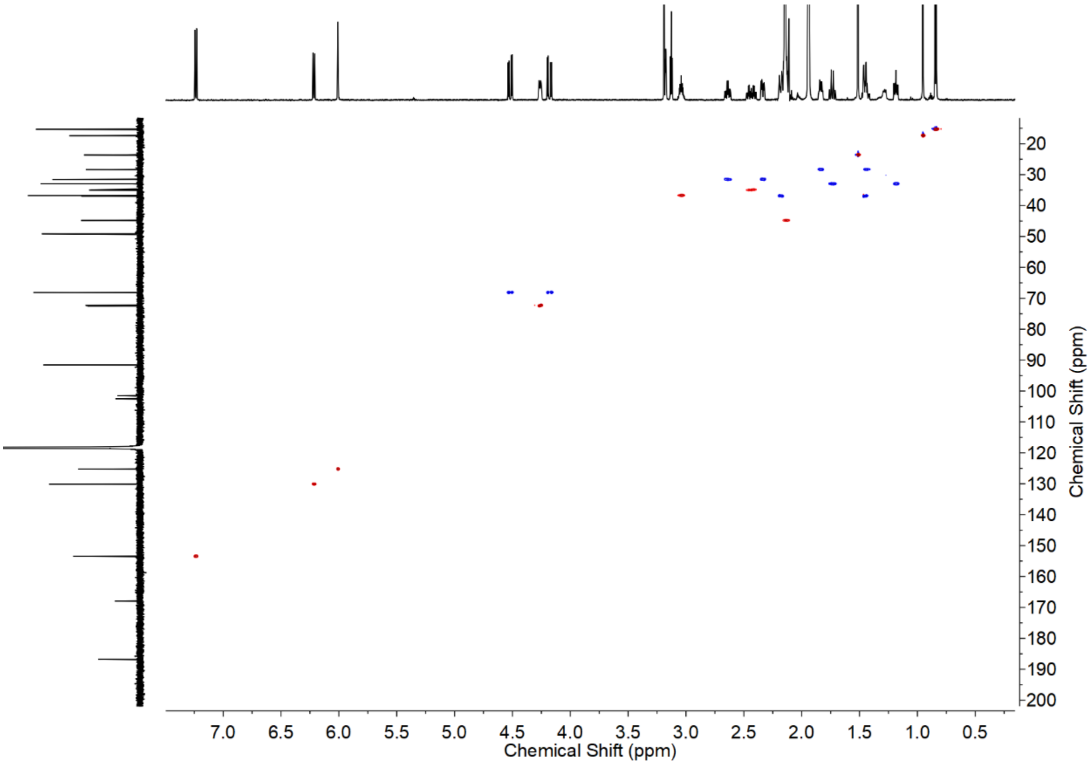

Figure S117: ^{1}H-^{13}C HSQC NMR spectrum of **G10** (700 MHz, CD_3CN, 298 K).

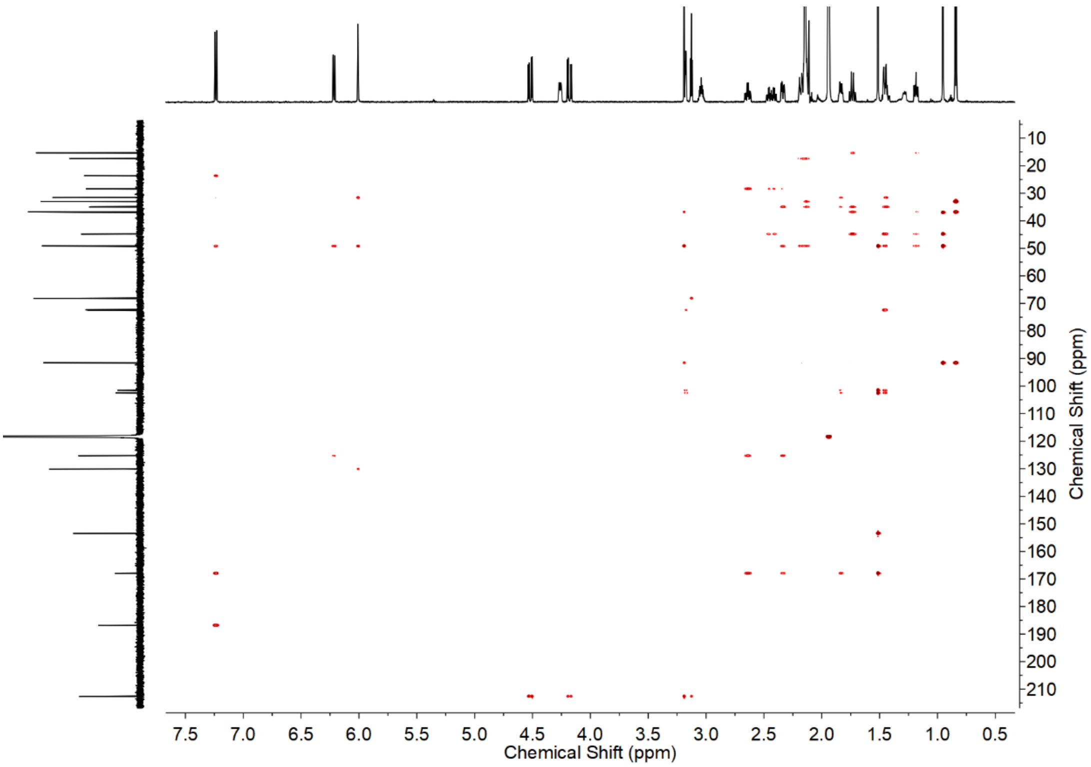

Figure S118: ^{1}H-^{13}C HMBC NMR spectrum of **G10** (700 MHz, CD_3CN, 298 K).

S7.4.2 **G11**

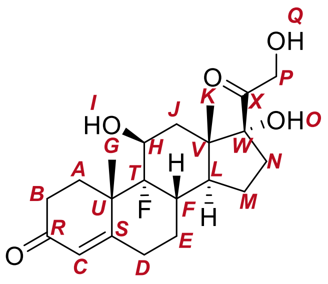

δ_H (700 MHz, CD_3CN, 298 K) 5.68 (s, 1H, H_C), 4.56 (dd, J = 19.5 Hz, 5.2 Hz, 1H, H_{P1}), 4.26 (ddd, J = 9.5, 6.2, 4.0 Hz, 1H, H_H), 4.20 (dd, J = 19.5 Hz, 5.5 Hz, 1H, H_{P2}), 3.36 (s, 1H, H_O), 3.11 (t, J = 5.3 Hz, 1H, H_Q), 2.92 (dd, J = 3.8, 2.1 Hz, 1H, H_I), 2.69 (ddd, J = 14.7, 11.5, 3.0 Hz, 1H, H_{N1}), 2.59 (td, J = 14.4 Hz, 6.2 Hz, 1H, H_{D2}), 2.48–2.33 (m, 3H, H_{A1}, H_{B1} and H_F), 2.29–2.24 (m, 2H, H_{B2} and H_{D1}), 2.20 (dt, J = 13.7 Hz, 2.9 Hz, 1H, H_{J1}), 2.17–2.11 (m, 1H, H_L), 1.98 (dt, J = 13.0 Hz, 4.1 Hz, 1H, H_{A2}), 1.80–1.77 (m, 1H, H_{E2}), 1.75–1.71 (m, 1H, H_{M2}), 1.52–1.46 (m, 6H, H_{N2}, H_{J2}, H_G and H_{E1}), 1.42–1.35 (m, 1H, H_{M1}), 0.83 (s, 3H, H_K).

δ_C (176 MHz, CD_3CN, 298 K) 212.7 (C_X), 199.1 (C_R), 170.3 (C_S), 150.0 (C_C), 101.1 (d, J = 170.7 Hz, C_T), 89.8 (C_W), 70.8 (d, J = 37.3 Hz, C_H), 67.7 (C_P), 47.5 (C_V), 46.3 (d, J = 1.7 Hz, C_L), 44.8 (d, J = 20.8 Hz, C_U), 36.5 (C_J), 35.0 (d, J = 19.7 Hz, C_F), 34.6 (C_N), 34.5 (C_B), 31.4 (C_D), 29.3 (C_A), 27.1 (C_E), 24.1 (C_M), 22.2 (d, J = Hz, C_G), 17.2 (C_K).

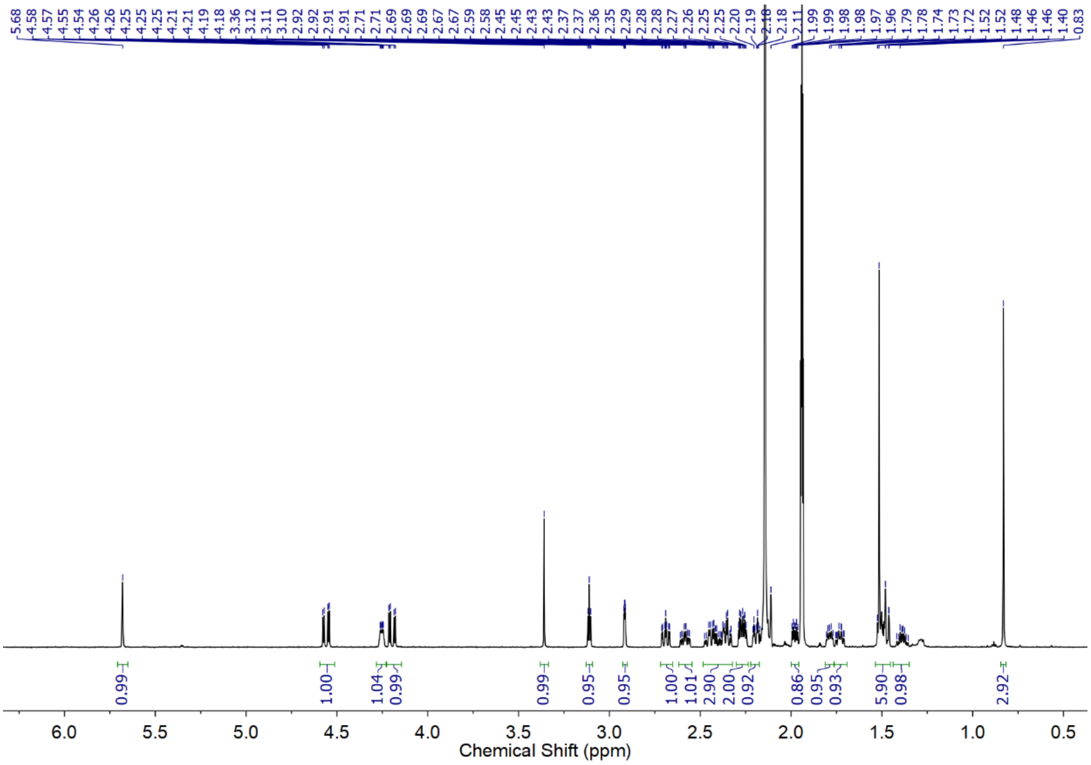

Figure S119: ^{1}H NMR spectrum of **G11** (700 MHz, CD_3CN, 298 K).

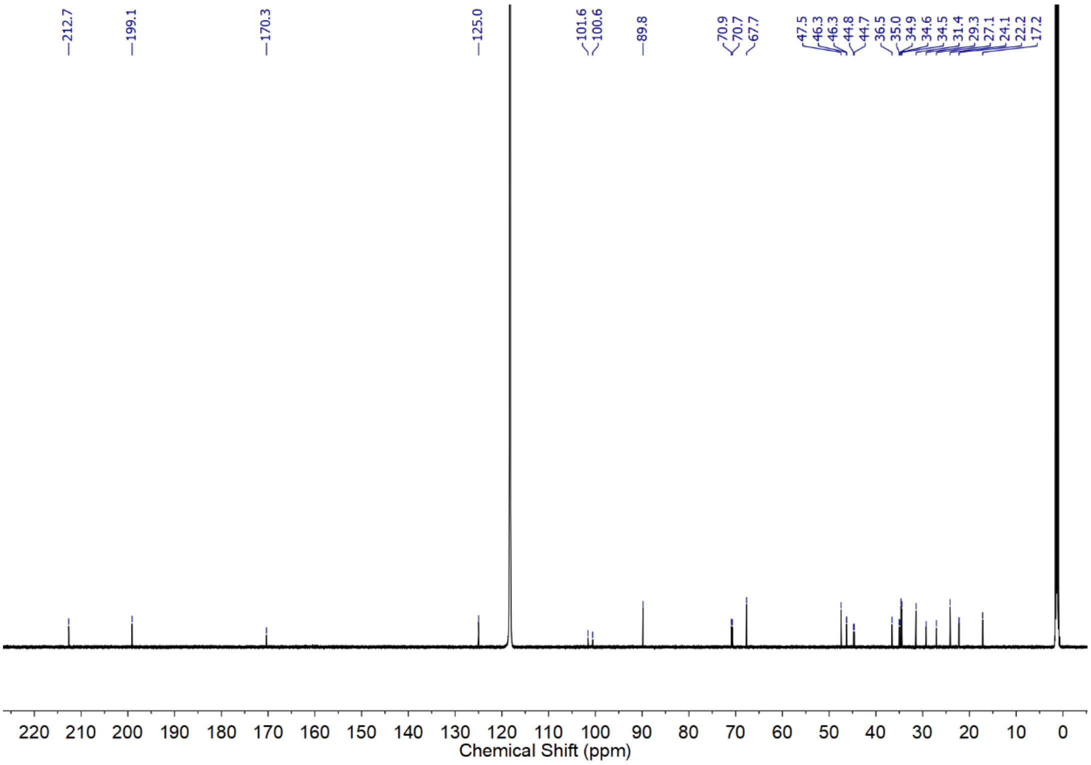

Figure S120: ^{13}C NMR spectrum of **G11** (176 MHz, CD_3CN, 298 K).

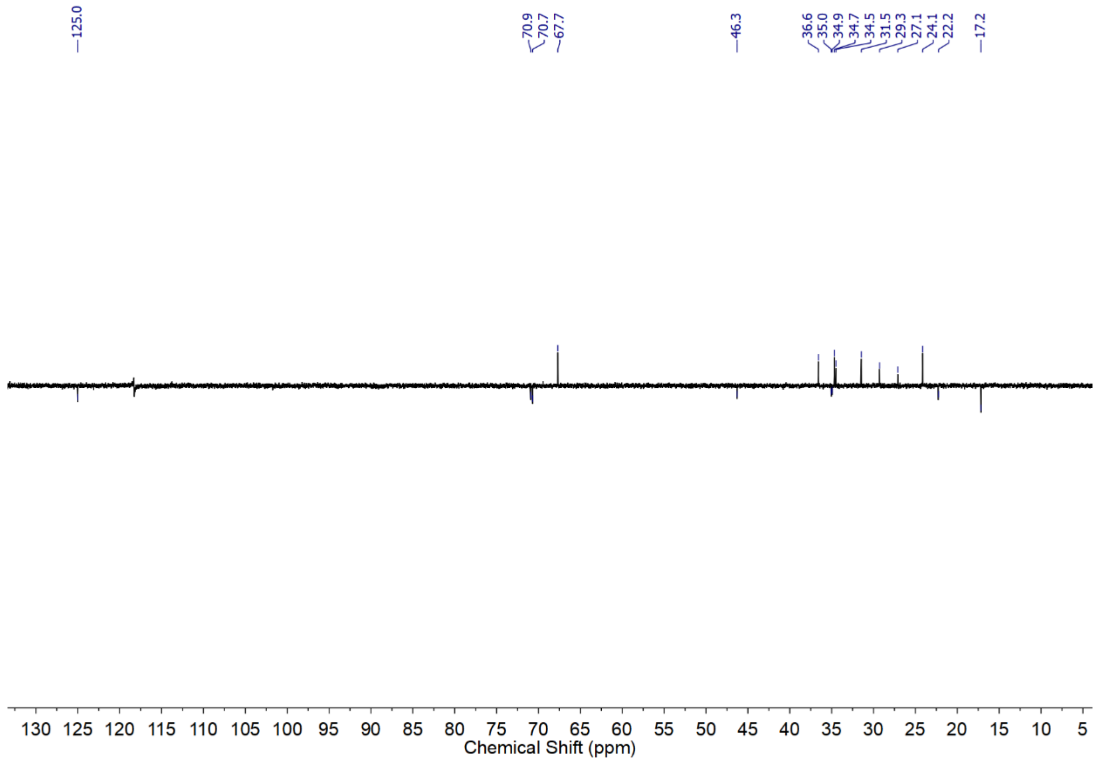

Figure S121: DEPT-135 NMR spectrum of **G11** (176 MHz, CD_3CN, 298 K).

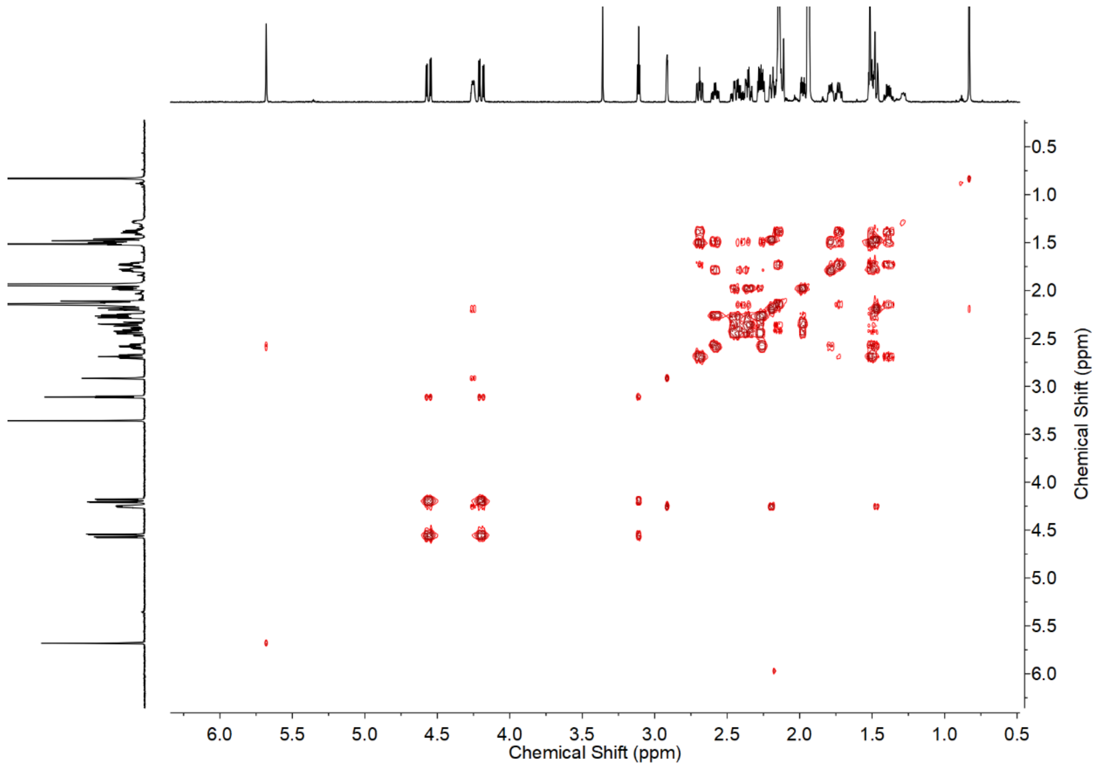

Figure S122: ^{1}H-^{1}H DQF-COSY NMR spectrum of **G11** (700 MHz, CD_3CN, 298 K).

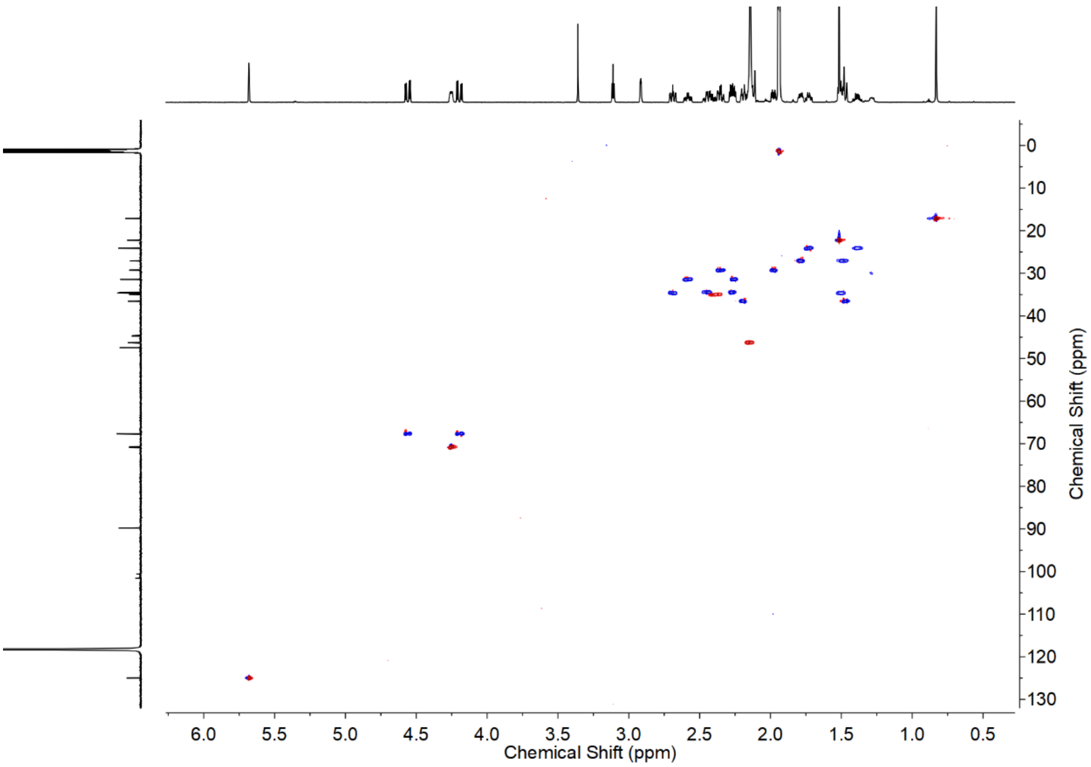

Figure S123: ^{1}H-^{13}C HSQC NMR spectrum of **G11** (700 MHz, CD_3CN, 298 K).

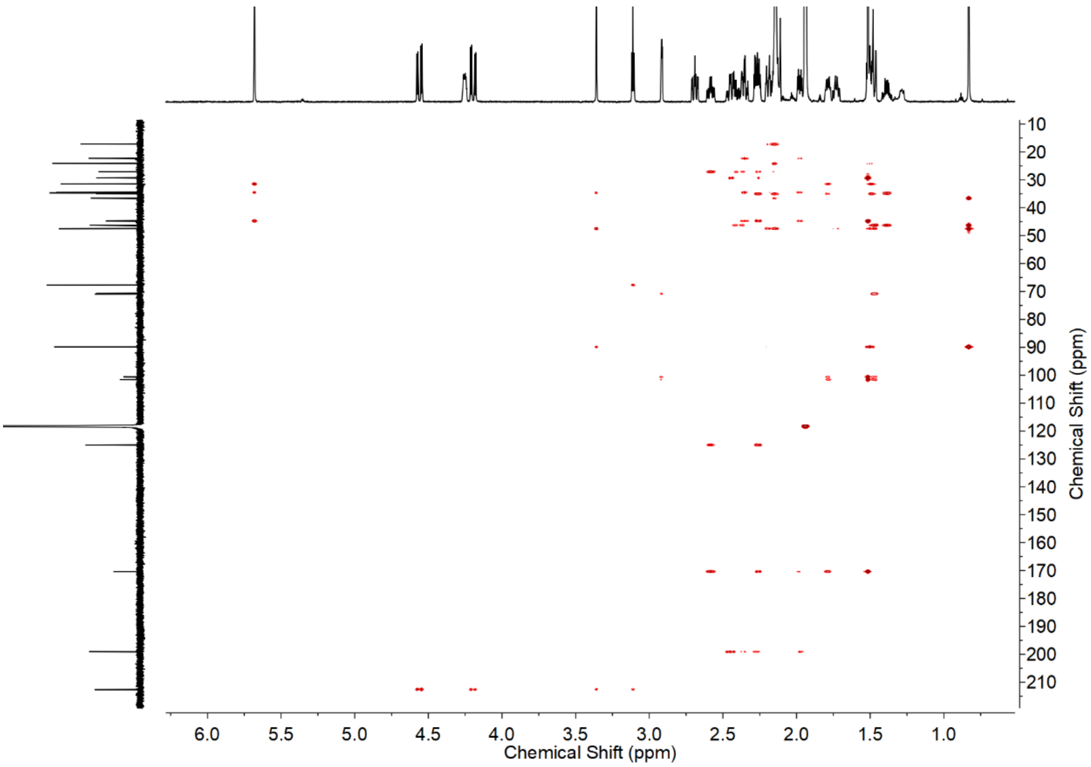

Figure S124: ^{1}H-^{13}C HMBC NMR spectrum of **G11** (700 MHz, CD_3CN, 298 K).

S8. References

(1) Allan, D. .; Nowell, H.; Barnett, S.; Warren, M.; Wilcox, A.; Christensen, J.; Saunders, L.; Peach, A.; Hooper, M.; Zaja, L.; Patel, S.; Cahill, L.; Marshall, R.; Trimnell, S.; Foster, A.; Bates, T.; Lay, S.; Williams, M.; Hathaway, P.; Winter, G.; Gerstel, M.; Wooley, R. A Novel Dual Air-Bearing Fixed-χ Diffractometer for Small-Molecule Single-Crystal X-Ray Diffraction on Beamline I19 at Diamond Light Source. *Crystals* **2017**, *7* (11), 336. https://doi.org/10.3390/cryst7110336.

(2) Gildea, R. J.; Winter, G. Determination of Patterson Group Symmetry from Sparse Multi-Crystal Data Sets in the Presence of an Indexing Ambiguity. *Acta Crystallogr. Sect. D Struct. Biol.* **2018**, *74* (5), 405–410. https://doi.org/10.1107/S2059798318002978.

(3) Evans, P. Scaling and Assessment of Data Quality. *Acta Crystallogr. Sect. D Biol. Crystallogr.* **2006**, *62* (1), 72–82. https://doi.org/10.1107/S0907444905036693.

(4) Winter, G. Xia2 : An Expert System for Macromolecular Crystallography Data Reduction. *J. Appl. Crystallogr.* **2010**, *43* (1), 186–190. https://doi.org/10.1107/S0021889809045701.

(5) Farrugia, L. J. WinGX and ORTEP for Windows : An Update. *J. Appl. Crystallogr.* **2012**, *45* (4), 849–854. https://doi.org/10.1107/S0021889812029111.

(6) Evans, P. R.; Murshudov, G. N. How Good Are My Data and What Is the Resolution? *Acta Crystallogr. Sect. D Biol. Crystallogr.* **2013**, *69* (7), 1204–1214. https://doi.org/10.1107/S0907444913000061.

(7) Winn, M. D.; Ballard, C. C.; Cowtan, K. D.; Dodson, E. J.; Emsley, P.; Evans, P. R.; Keegan, R. M.; Krissinel, E. B.; Leslie, A. G. W.; McCoy, A.; McNicholas, S. J.; Murshudov, G. N.; Pannu, N. S.; Potterton, E. A.; Powell, H. R.; Read, R. J.; Vagin, A.; Wilson, K. S. Overview of the CCP 4 Suite and Current Developments. *Acta Crystallogr. Sect. D Biol. Crystallogr.* **2011**, *67* (4), 235–242. https://doi.org/10.1107/S0907444910045749.

(8) Sheldrick, G. M. SHELXT – Integrated Space-Group and Crystal-Structure Determination. *Acta Crystallogr. Sect. A Found. Adv.* **2015**, *71* (1), 3–8. https://doi.org/10.1107/S2053273314026370.

(9) Sheldrick, G. M. Crystal Structure Refinement with SHELXL. *Acta Crystallogr. Sect. C Struct. Chem.* **2015**, *71* (1), 3–8. https://doi.org/10.1107/S2053229614024218.

(10) van der Sluis, P.; Spek, A. L. BYPASS: An Effective Method for the Refinement of

Crystal Structures Containing Disordered Solvent Regions. *Acta Crystallogr. Sect. A Found. Crystallogr.* **1990**, *46* (3), 194–201. https://doi.org/10.1107/S0108767389011189.

(11) Spek, A. L. PLATON: A Multipurpose Crystallographic Tool. University of Utrecht: Utrecht, The Netherlands 2008.

(12) Wilcox, C. S. *Frontiers in Supramolecular Organic Chemistry and Photochemistry*; Schneider, H. J., Dürr, H., Eds.; VCH: Weinheim, 1991.

(13) Moss, G. P. Nomenclature of Steroids (Recommendations 1989). *Pure Appl. Chem.* **1989**, *61* (10), 1783–1822.

(14) Dupont, L.; Dideberg, O.; Campsteyn, H. Structure Cristalline et Moléculaire Du 9α-Fluorocortisol, $C_{21}H_{29}O_5F$. *Acta Crystallogr. Sect. B Struct. Crystallogr. Cryst. Chem.* **1972**, *28* (10), 3023–3032. https://doi.org/10.1107/S0567740872007381.
